# AXL–SHC1 signaling axis mediates adaptive resistance to HER2-targeted tyrosine kinase inhibitors in HER2-aberrant lung and gastric cancers

**DOI:** 10.1038/s41698-026-01385-2

**Published:** 2026-04-02

**Authors:** Masaki Ishida, Tadaaki Yamada, Yuki Katayama, Hayato Kawachi, Ryo Sawada, Ryota Nakamura, Soichi Hirai, Yohei Matsui, Kenji Morimoto, Keisuke Onoi, Aosa Nakamura-Sasada, Mano Horinaka, Toshiyuki Sakai, Tomoki Sakakida, Toshifumi Doi, Kazuo Yasumoto, Yasuhiro Goto, Naoki Furuya, Hirokazu Taniguchi, Hirokazu Ogino, Nakano Takayuki, Yusuke Chihara, Koji Fukuda, Hiroaki Taniguchi, Hisanori Uehara, Seiji Yano, Shinsaku Tokuda, Koichi Takayama

**Affiliations:** 1 Department of Pulmonary Medicine, Graduate School of Medical Science, Kyoto Prefectural University of Medicine, 465, Kajii-cho, Kamigyo-ku, Kyoto, Japan; 2 Department of Drug Discovery Medicine, Graduate School of Medical Science, Kyoto Prefectural University of Medicine, 465, Kajii-cho, Kamigyo-ku, Kyoto, Japan; 3 Department of Molecular Gastroenterology and Hepatology, Graduate School of Medical Science, Kyoto Prefectural University of Medicine, Kyoto, Japan; 4 Department of Medical Oncology, Kanazawa Medical University, Uchinada, Ishikawa, Japan; 5 Department of Respiratory Medicine, Fujita Health University School of Medicine, Toyoake, Aichi Japan; 6 Department of Respiratory Medicine, St. Marianna University School of Medicine, Kawasaki, Japan; 7 Department of Respiratory Medicine, Nagasaki University Graduate School of Biomedical Sciences, Nagasaki, Japan; 8 Department of Respiratory Medicine and Rheumatology, Graduate School of Biomedical Sciences, Tokushima University, Tokushima, Japan; 9 Department of Respiratory Medicine, Fukuchiyama City Hospital, Kyoto, Japan; 10 Department of Respiratory Medicine, Uji Tokushukai Medical Center, Uji, Kyoto, Japan; 11 Division of Innovative Cancer Control Research, Cancer Research Institute, Kanazawa University, Kanazawa, Japan; 12 Department of Diagnostic Pathology, Tokushima University Hospital, Tokushima, Japan; 13 Department of Respiratory medicine, Kanazawa Graduate School of Medical Sciences, Kanazawa, Japan

**Keywords:** Predictive markers, Non-small-cell lung cancer, Gastric cancer, Molecular medicine, Translational research

## Abstract

Human epidermal growth factor receptor 2 (HER2)-tyrosine kinase inhibitors (TKIs) are being developed for the treatment of patients with HER2-aberrant lung and gastric cancers. However, achieving complete tumor remission remains challenging. Here, we investigated the molecular mechanisms underlying adaptive resistance to HER2-TKIs in HER2-aberrant tumor cells in order to devise strategies to prevent the emergence of drug-tolerant cells. Our findings showed that the AXL receptor was activated by HER2-TKIs and maintained cell survival by interacting with epidermal growth factor receptor, HER2, and HER3. This process, mediated by the SHC1BP–SHC1 axis, contributed to adaptive resistance to HER2-TKIs in a subset of HER2-aberrant lung and gastric cancers. AXL inhibition significantly delayed tumor regrowth of AXL-overexpressing cells by enhancing HER2-TKI-induced apoptosis in xenograft models. These results suggest that patients with HER2-aberrant lung and gastric cancers exhibiting high AXL expression may benefit from an initial combination therapy with an AXL inhibitor.

## Introduction

In recent years, various driver gene alterations have been identified across different cancer types. This has spurred significant efforts to develop molecularly targeted therapies that inhibit these alterations, which result in substantial clinical benefits^1–4^. Among these targets, human epidermal growth factor receptor 2 (HER2), a member of the ErbB family of receptor tyrosine kinases, plays a pivotal role in regulating complex signaling networks that govern tissue development, growth, and homeostasis in key organs such as the heart, nervous system, and breasts^5^. HER2 overexpression is strongly linked to the growth, proliferation, and survival of cancer cells primarily through the activation of signaling pathways such as PI3K/AKT and MAPK pathways. HER2 aberrations are observed in approximately 20%–25% of breast cancer, 10–20% of gastric cancer, and 2–5% of lung cancer cases 2, 6–9.

The activation of HER2 signaling represents a critical therapeutic target for HER2-aberrant cancer. Several HER2-targeted therapies, including tyrosine kinase inhibitors (TKIs) and antibody–drug conjugates, have shown efficacy in controlling these tumors. HER2-TKIs, such as mobocertinib, poziotinib, and tucatinib, have been developed for clinical use in HER2-aberrant cancer. Mobocertinib and poziotinib, potent irreversible TKIs targeting the epidermal growth factor receptor (EGFR) and HER2 exon 20 insertions, have demonstrated efficacy against HER2-altered non-small cell lung cancer (NSCLC) in preclinical and early-phase clinical trials^10–13^. Tucatinib, in combination with trastuzumab and capecitabine, was approved by the FDA following the HER2CLIMB trial (NCT02614794), which reported an objective response rate of 40.6%, a median progression-free survival of 7.8 months, and an overall survival of 21.9 months in 410 patients with HER2-aberrant breast cancer^14^. Furthermore, a phase 2 basket trial is investigating the combination of tucatinib and trastuzumab in patients with HER2-aberrant solid tumors (NCT04579380). In addition to these agents, zongertinib (BI 1810631), a highly selective HER2-TKI that spares wild-type EGFR, has shown promising efficacy in HER2-aberrant NSCLC, with a confirmed objective response rate of 71 percent and a median progression-free survival of 12.4 months. These findings led to its breakthrough therapy designation by the U.S. FDA^15^. Sevabertinib (BAY 2927088), a reversible HER2-TKI targeting HER2 exon 20 insertion mutations, also demonstrated substantial antitumor activity in advanced HER2-mutant NSCLC in the SOHO-01 trial and similarly received U.S. FDA breakthrough therapy designation^16^. These next-generation TKIs further expand the therapeutic landscape for patients with HER2-aberrant cancers. However, despite advancements in molecularly targeted therapies for HER2-aberrant cancer, complete responses to HER2-TKIs remain rare. Resistance to HER2-TKIs, as with other targeted therapies, is an inevitable challenge. The mechanism of resistance to HER2-TKIs has been reported mainly in breast cancer^17,18^.

In addition, the mechanisms of drug resistance in HER2-targeted therapies generally include aberrant activation of HER2 and downstream signaling pathways, such as amplification, upregulation, or mutations in HER2, PIK3CA, and AKT, which undermine the inhibition of downstream signaling and tumor growth^19,20^. However, the resistance mechanisms in other HER2-aberrant cancers, such as gastric cancer and NSCLC, remain unclear. In patients with NSCLC, anti-HER2 antibody–drug conjugates, such as trastuzumab deruxtecan, have demonstrated promising efficacy^21^. However, acquired resistance to these agents poses a growing challenge. Reported resistance mechanisms include HER2 protein downregulation, drug efflux, alterations in intracellular trafficking, and mutations affecting the payload sensitivity^22^. These mechanisms may differ from those observed with HER2-TKIs, highlighting the complexity of resistance landscapes in HER2-aberrant NSCLC. Therefore, evaluating treatment resistance specific to these cancer types and developing targeted strategies to address these challenges is necessary. Recent studies have highlighted that small subsets of tumor cells can survive therapeutic pressure by entering drug-tolerant states^23,24^. These persistent cells can serve as a reservoir for subsequent resistance, often relying on alternative signaling pathways to maintain survival. Understanding the molecular basis of such early tolerance states is essential to effectively prevent or delay resistance acquisition. In addition, molecular targeted therapies drive tumor evolution and intratumoral heterogeneity, which ultimately results in acquired resistance^25^. Consequently, overcoming this resistance requires early and effective therapeutic interventions to improve the survival rates for patients with HER2-aberrant cancer. Preclinical studies have demonstrated that effectively inhibiting tolerance signals can significantly increase cell death in tumor cells with driver oncogene alterations^26–28^. Given this context, combination therapies involving HER2-TKIs may play a critical role in improving clinical outcomes. However, the molecular mechanisms underlying adaptive resistance to HER2-TKIs and effective strategies to counteract this resistance remain unclear. This study aimed to uncover the molecular mechanisms driving adaptive resistance to HER2-TKIs in HER2-aberrant lung and gastric cancer cells and develop novel therapeutic strategies to overcome this challenge.

## Results

### AXL played a crucial role in the survival of HER2-aberrant NSCLC and gastric cancer cells treated with HER2-TKIs

**Fig. 1:**
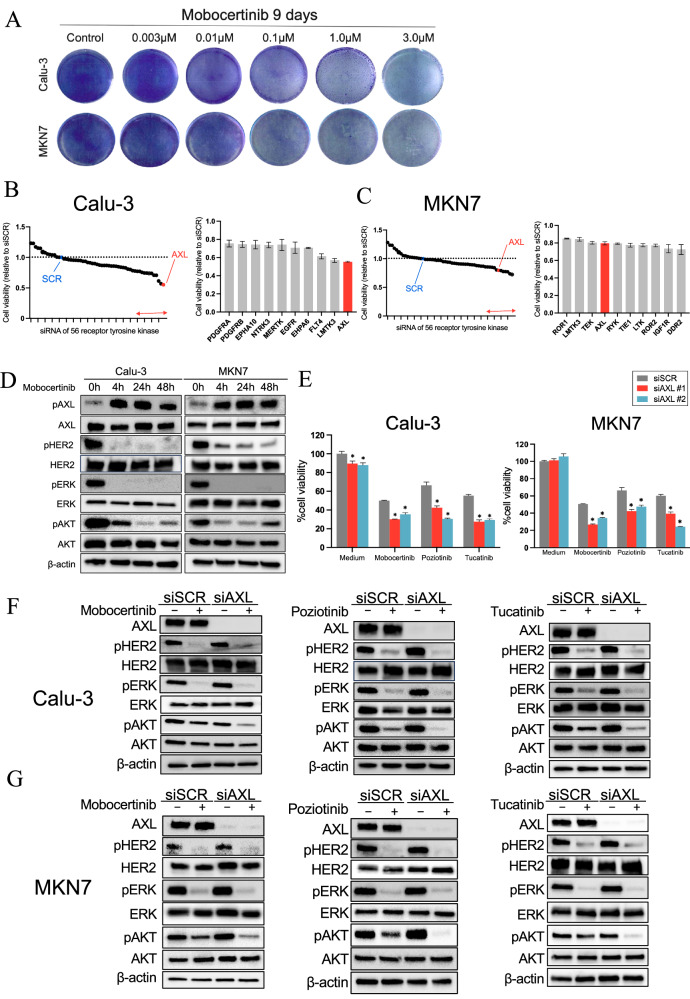
AXL plays a pivotal role in the survival of HER2-aberrant tumor cancers. **A** Calu-3 and MKN7 cell viability after 9 days of treatment with the indicated concentrations of mobocertinib, replenished every 72 h. MTT assays evaluating the effect of a combination of mobocertinib (0.1 or 0.01 μmol/L) and knockdown of 56 receptor tyrosine kinases on **B** Calu-3 and **C** MKN7 cell viability in comparison to cells treated with nonspecific control siRNA and incubated with mobocertinib. MTT assays were conducted to evaluate the impact of a combination of mobocertinib for 72 h and the knockdown of 56 receptor tyrosine kinases sourced from the Silencer® Select human kinase siRNA library. The effects of the top 10 genes indicated by a red line in the panel are shown. **D** Western blotting of Calu-3 and MKN7 cells treated with mobocertinib (0.01 or 0.1 μmol/L) for the indicated durations. **E** MTT assays assessing cell viability were assessed in Calu-3 and MKN7 treated with nonspecific control siRNA or AXL-specific siRNAs, and incubated with or without mobocertinib *P < *0.05* (one-way ANOVA). Western blotting of **F** Calu-3 and **G** MKN7 cells treated with nonspecific control siRNA or AXL-specific siRNA, and incubated with or without mobocertinib for 4 h.

We first evaluated the sensitivity of eight HER2-aberrant cancer cell lines to the HER2-TKIs mobocertinib, poziotinib, and tucatinib. The cancer cells displayed varying levels of sensitivity to these HER2-TKIs (Supplementary Fig. 1). Notably, Calu-3 cells (NSCLC) exhibited moderate sensitivity, while MKN7 cells (gastric cancer) exhibited low sensitivity to HER2-TKIs. Both cell lines were able to survive prolonged exposure to high concentrations of mobocertinib (up to 3 µmol/L) for 9 days (Fig. 1A). To explore the molecular mechanism underlying adaptive resistance to mobocertinib, synthetic lethality screening was conducted using an siRNA library to identify the factors associated with adaptive resistance to mobocertinib treatment. Among the 56 receptor-type tyrosine kinase siRNAs, AXL knockdown consistently improved mobocertinib efficacy in both Calu-3 and MKN7 cells (Fig. 1B, C). Other genes that demonstrated stronger inhibitory activity than AXL in MKN7 cells were further evaluated in Calu-3 cells but did not show significant effects (Supplementary Fig. 2). Therefore, AXL was selected for further investigation due to its additional impact on reducing cell viability and its potential clinical relevance. Western blotting revealed that mobocertinib treatment induced the phosphorylation of AXL at 4 h, which persisted at 48 h. Phosphorylation of ERK, a downstream effector of HER2, was suppressed by mobocertinib at 4 h, and this suppression was maintained at 48 h. In contrast, AKT, another downstream effector of HER2 signaling, was only partially inhibited by mobocertinib at 4 h and reactivated at 48 h (Fig. 1D). Knockdown of AXL using two independent siRNAs enhanced the efficacy of HER2-TKIs, including mobocertinib, poziotinib, and tucatinib, in reducing the viability of Calu-3 and MKN7 cells (Fig. 1E). Western blotting showed that combined AXL knockdown and HER2-TKI treatment also inhibited AKT phosphorylation (Ser473) more effectively than HER2-TKIs alone in both cell lines at 4 h (Fig. 1F, G). Densitometric analysis of p-AKT normalized to the loading control (β-actin) using ImageJ showed that AXL knockdown combined with HER2-TKIs reduced p-AKT/β-actin ratios compared with HER2-TKIs treatment alone in both Calu-3 and MKN7 cells (Supplementary Fig. 3A, B). In addition, analysis of other AKT phosphorylation sites (Thr308 and Tyr326) and downstream effectors, including mTOR (Ser2448) and p70S6K (Ser424 and Thr389), revealed that the combination of AXL knockdown and mobocertinib at 4 h selectively reduced phosphorylation of mTOR (Ser2448) and p70S6K (Ser424). In contrast, phosphorylation at the remaining sites was unchanged (Supplementary Fig. 4A).

These results demonstrate that AXL plays a critical role in the adaptive resistance of a subset of HER2-aberrant NSCLC and gastric cancer cells to HER2-TKIs, with its effects mediated via the AKT signaling pathway.

### Inhibition of AXL-sensitized HER2-aberrant cancer cells to HER2-TKIs

**Fig. 2:**
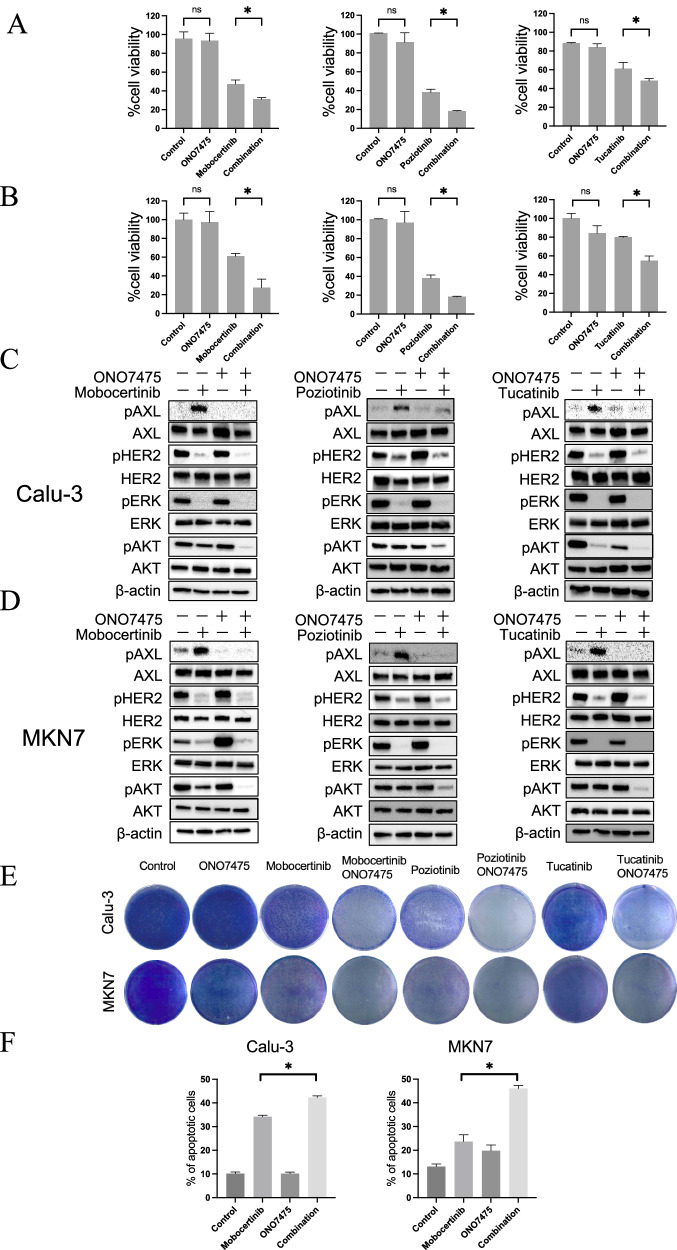
Combination therapy with HER2-TKIs and ONO7475 inhibits cell proliferation by inducing apoptosis. Cell viability of **A** Calu-3 and **B** MKN7 cells was assessed after incubation with mobocertinib (0.01 or 0.1 μmol/L) in the presence or absence of the AXL inhibitor ONO7475 (0.3 μmol/L) for 72 h using MTT assays. Western blotting analysis of **C** Calu-3 and **D** MKN7 cells treated with mobocertinib (0.01 or 0.1 μmol/L) with or without ONO7475 (0.3 μmol/L) for 4 h. **E** Visualization of Calu-3 and MKN7 cells using crystal violet staining after 14 days of treatment with HER2-TKIs, ONO-7475 (0.3 µmol/L), or a combination of HER2-TKIs and ONO7475. Calu3 cell was treated with 0.01 µmol/L mobocertinib, 0.01 nmol/L poziotinib, 0.1 µmol/L tucatinib, 0.3 μmol/L ONO7475, or a combination of each HER2-TKI and 0.3 μmol/L ONO-7475 for 14 days. MKN7 cells were treated with 0.1 µmol/L mobocertinib, 0.1 nmol/L poziotinib, 1.0 µmol/L tucatinib, 0.3 μmol/L ONO7475, or a combination of each HER2-TKI and 0.3 μmol/L ONO-7475 for 14 days. **F** Flow cytometry analysis of apoptotic cell percentages in Calu-3 and MKN7 cells treated with mobocertinib (0.01 or 0.1 μmol/L), ONO7475 (0.3 µmol/L), or a combination of mobocertinib and ONO7475 for 48 h. *P < 0.05 (one-way ANOVA).

To evaluate the therapeutic potential of targeting HER2 and AXL in HER2-aberrant cancer cells, we examined the effects of combining AXL inhibitors with HER2-TKIs on cell viability. The combination of the AXL inhibitor ONO7475 with HER2-TKIs for 72 h significantly increased cell sensitivity to HER2-TKI monotherapy and reduced the viability of Calu-3 and MKN7 cells (Fig. 2A, B). Synergy analysis using the Bliss independence model revealed a clear synergistic interaction between HER2-TKIs and ONO7475 at these concentrations. In addition, the combination treatment reduced the IC₅₀ values to a greater extent than did the mobocertinib monotherapy (Supplementary Fig. 5A–C). Similarly, treatment with another AXL inhibitor, NPS1034, in combination with HER2-TKIs for 72 h significantly reduced the cell viability of these cell lines (Supplementary Fig. 6A–C). However, ONO7475, in combination with HER2-TKIs, exhibited only marginal effects on the viability of other HER2-aberrant cancer cell lines, including NUGC4, SKBR3, MDA-MB-453, H2170, H1781, and HCC1954 (Supplementary Fig. 7). Next, western blotting analysis was performed to investigate protein expression and elucidate the signaling pathways involved in the combined effect of ONO7475 and HER2-TKIs. Treatment with the combination of HER2-TKIs and ONO7475 for 4 h markedly inhibited the phosphorylation of AXL and AKT compared to HER2-TKI monotherapy in both Calu-3 and MKN7 cells (Fig. 2C, D). Continuous co-treatment with HER2-TKIs and ONO7475 for 14 days reduced the cell viability of Calu-3 and MKN7 cells (Fig. 2E). To further evaluate the efficacy of AXL inhibitors, cell cycle and apoptosis assays were performed. In MKN7 cells, the combination of mobocertinib and ONO7475 induced greater G1-phase arrest than mobocertinib monotherapy, whereas this effect was not observed in Calu-3 cells (Supplementary Fig. 8A, B). Apoptosis analysis revealed that mobocertinib alone induced apoptosis in both Calu-3 and KN7 cells; however, this effect was significantly enhanced when combined with ONO7475, thereby demonstrating a stronger apoptotic response (Fig. 2F and Supplementary Fig. 9A, B).

These results indicate that the combination of HER2-TKIs with the AXL inhibitor ONO7475 sensitized HER2-aberrant cancer cells to HER2-TKIs, which leads to reduced cell viability and increased apoptosis.

### GAS6-AXL signaling axis in adaptive resistance to HER2-TKIs

**Fig. 3:**
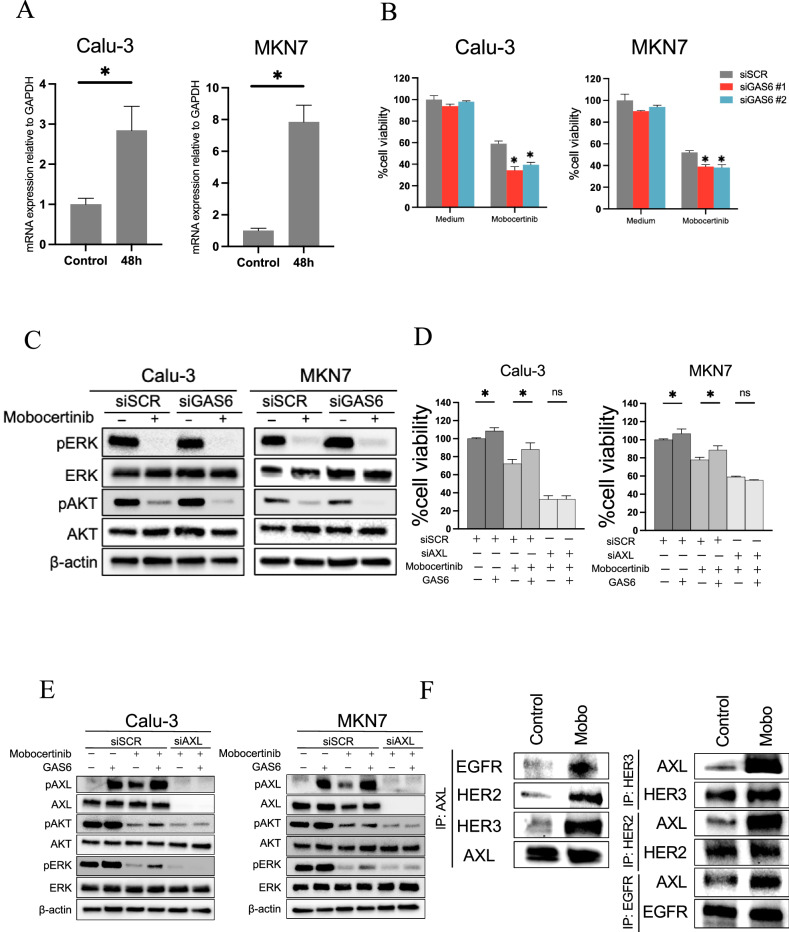
GAS6–AXL axis-induced drug-tolerant cells with HER2-TKIs. **A** qPCR analysis of GAS6 expression in Calu-3 and MKN7 parent cells treated with nonspecific (control) or GAS6-specific siRNAs for 48 h. *P < 0.05 (unpaired t-tests). **B** MTT assays evaluating cell viability of Calu-3 and MKN7 cells treated with nonspecific or GAS6-specific siRNAs were incubated with or without mobocertinib (0.01 or 0.1 μmol/L) for 72 h. These cell lines were incubated in an RPMI 1640 medium with 0.1% fetal bovine serum, penicillin (100 U/mL), and streptomycin (100 μg/mL) in a humidified 5% CO2 incubator at 37 °C for 72 h (*P < 0.05, one-way ANOVA). **C** Western blotting analysis of Calu-3 and MKN7 cells transfected with nonspecific or GAS6-specific siRNAs for 48 h, followed by treatment with or without mobocertinib (0.01 or 0.1 μmol/L) for 4 h. **D** Calu-3 and MKN7 cells with nonspecific siRNA or AXL-specific siRNAs were treated for 72 h with or without mobocertinib (0.01 or 0.1 μmol/L) and/or GAS6 (50 ng/mL). Cell growth was determined using MTT assays. *P < 0.05 (two-way ANOVA). **E** Western blotting of Calu-3 and MKN7 cells transfected with nonspecific or AXL-specific siRNAs and treated with or without mobocertinib (0.01 or 0.1 μmol/L) for 4 h and GAS6 (50 ng/mL) for 15 min. **F** MKN7 cells treated with or without mobocertinib (Mobo, 0.1 μmol/L) for 24 h were detected by western blotting with immunoprecipitation of the indicated proteins.

To investigate the mechanisms underlying AXL activation during HER2-TKI treatment, we examined the production of the AXL ligand GAS6. The GAS6 mRNA levels were significantly increased by mobocertinib treatment in both Calu-3 and MKN7 cells at 48 h compared to those in the controls (Fig. 3A). GAS6 knockdown using two different siRNAs enhanced the efficacy of mobocertinib in reducing the viability of HER2-aberrant cancer cells (Fig. 3B). GAS6 knockdown was confirmed using quantitative real-time PCR (Supplementary Fig. 10). Western blotting showed that the combination of GAS6 knockdown and mobocertinib suppressed AKT phosphorylation more effectively than mobocertinib alone (Fig. 3C). Conversely, exogenous GAS6 stimulation reduced the efficacy of mobocertinib in both Calu-3 and MKN7 cells. This inhibitory effect was reversed by AXL knockdown (Fig. 3D). Further, GAS6 stimulation induced mobocertinib-mediated phosphorylation of AXL, AKT, and ERK, while AXL knockdown abrogated these effects (Fig. 3E). Immunoprecipitation experiments were performed to further investigate the mechanism underlying AXL activation. These revealed that mobocertinib treatment for 24 h enhanced the binding of AXL to EGFR, HER2, and HER3 (Fig. 3F).

These findings suggest that AXL activation by mobocertinib, through its interaction with EGFR, HER2, and HER3, sustains cell survival and contributes to AKT signaling in HER2-aberrant cancer cells. The GAS6-AXL signaling axis, activated via GAS6 ligand binding and receptor dimerization, plays a key role in the adaptive resistance to HER2-TKIs.

### SHC1–SHCBP1 complex contributed to HER2-TKIs sensitivity

**Fig. 4:**
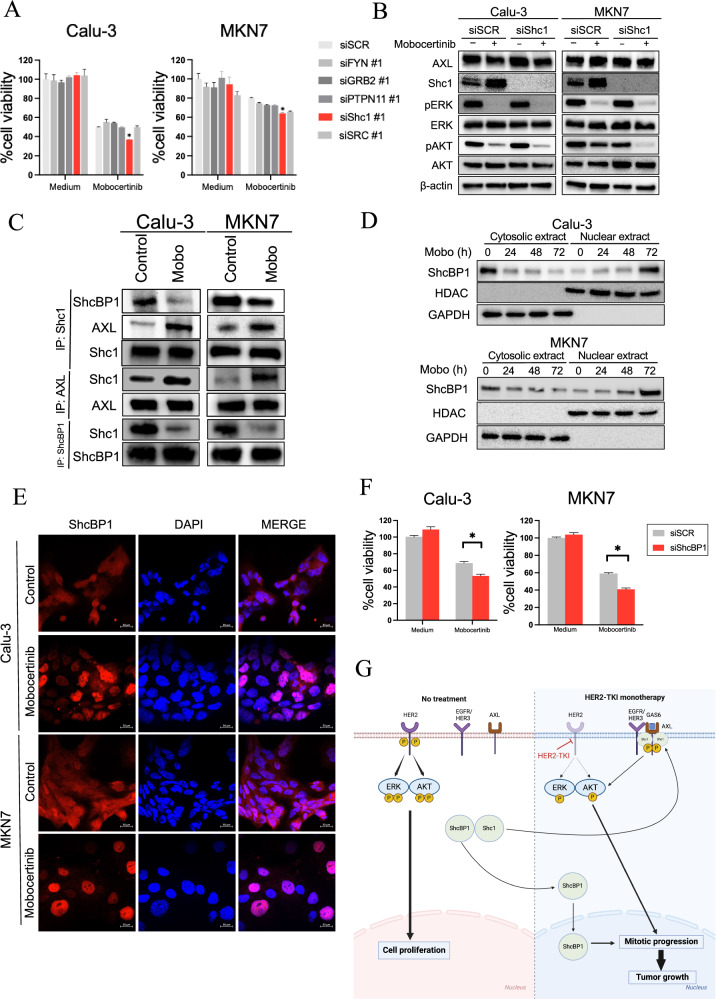
Shc1–ShcBP1 axis activates AXL signaling and induces adaptive resistance to HER2-TKIs. **A** Combination of mobocertinib and knockdown of several adapter proteins in Calu-3. and MKN7 cells. Calu-3 and MKN7 cells treated with nonspecific control, FYN, GRB2, PTPN11, Shc1, or SRC-specific siRNAs were incubated with or without mobocertinib (0.01 or 0.1 μmol/L) for 72 h, and cell viability was detected using MTT assays. *P < 0.05 compared with nonspecific control siRNA (two-way ANOVA). Data are represented as the mean ± S.D. **B** Western blotting of Calu-3 and MKN7 cells treated with nonspecific control siRNA or Shc1-specific siRNA and incubated with or without mobocertinib (0.01 or 0.1 μmol/L) for 24 h. **C** Calu-3 and MKN7 cells treated with or without mobocertinib (Mobo; 0.01 or 0.1 μmol/L) for 24 h were detected by western blotting with immunoprecipitation of the indicated proteins. **D** Western blotting showing nuclear localization of ShcBP1 in Calu-3 and MKN7 cells treated with mobocertinib (0.01 or 0.1 μmol/L) for 72 h. **E** Immunofluorescence showing nuclear localization of ShcBP1 in Calu-3 and MKN7 cells treated with mobocertinib (0.01 or 0.1 μmol/L) for 72 h. **F** MTT assays assessing cell viability of Calu-3 and MKN7 treated with nonspecific siRNA or ShcBP1-specific siRNAs, and incubated with or without mobocertinib *P < 0.05 (one-way ANOVA). **G** Schematic diagram illustrating the drug tolerance mechanisms, including GAS6–AXL activation through the SHC1BP–SHC1 complex, in HER2-aberrant cancer cells.

To further elucidate the molecular mechanisms underlying the adaptive resistance to mobocertinib, we investigated several adapter proteins and downstream signaling regulators associated with receptor tyrosine kinases, which recruit targets to the cytoplasm and amplify downstream signals, activating the MAPK and PI3K/AKT pathways. As signaling hubs, they dynamically interact with multiple binding partners to regulate the temporal flow of signaling following growth factor stimulation^29^. Among the adapter proteins analyzed, knockdown was performed using two independent siRNAs for each target. The knockdown of SHC1 was the most effective in reducing the viability of mobocertinib-treated Calu-3 and MKN7 cells (Fig. 4A and Supplementary Fig. 11). Western blotting showed that combining SHC1 knockdown with mobocertinib treatment suppressed AKT phosphorylation more effectively than mobocertinib monotherapy in both cell lines. A slight decrease in ERK phosphorylation was also observed (Fig. 4B). Densitometric analysis of p-AKT normalized to the loading control (β-actin) using ImageJ showed that SHC1 knockdown combined with mobocertinib reduced p-AKT/β-actin ratios to a greater extent than did mobocertinib monotherapy, in both Calu-3 and MKN7 cells (Supplementary Fig. 12). In addition, analysis of other AKT phosphorylation sites (Thr308 and Tyr326) and downstream effectors, including mTOR (Ser2448) and p70S6K (Ser424 and Thr389), revealed that the combination of SHC1 knockdown and mobocertinib at 4 h selectively reduced phosphorylation of mTOR (Ser2448) and p70S6K (Ser424). In contrast, phosphorylation at the remaining sites was unchanged (Supplementary Fig. 4B). Immunoprecipitation experiments revealed that SHC1 bound to AXL and dissociated from Shc SH2-domain binding protein 1 (SHCBP1) following 24 h of mobocertinib treatment (Fig. 4C). In addition, mobocertinib treatment at 24 h induced phosphorylation of SHC1, suggesting that the interaction between SHC1 and AXL is phosphorylation dependent (Supplementary Fig. 13). Next, to assess the impact of mobocertinib on SHCBP1 activity, we examined its intracellular localization. Both western blotting and immunofluorescence analyses revealed that mobocertinib treatment induced the translocation of SHCBP1 from the cytoplasm to the nucleus after 72 h (Fig. 4D, E). Knockdown of SHCBP1 further enhanced mobocertinib efficacy in Calu-3 and MKN7 cells (Fig. 4F). Moreover, flow cytometry analysis showed that mobocertinib combined with SHCBP1 knockdown induced significant G2/M arrest in MKN7 cells compared to that induced by mobocertinib monotherapy (Supplementary Fig. 14A). SHCBP1 knockdown was confirmed by western blotting (Supplementary Fig. 14B). These results suggest that the Shc1-ShcBP1 complex contributes to AXL activation, thereby promoting adaptive resistance to HER2-TKIs. To examine whether EGFR–HER3 bypass signaling contributes to this resistance mechanism, we investigated the addition of afatinib, a pan-ErbB inhibitor targeting EGFR and HER3, to the combination of mobocertinib and ONO7475. However, afatinib did not further decrease cell viability in Calu-3 and MKN7 cells compared with the dual combination therapy, suggesting that EGFR–HER3 signaling is unlikely to have a major bypass effect on AXL-mediated resistance under these conditions (Supplementary Fig. 15). Therefore, targeting the AXL–SHC1–SHCBP1 axis remains a promising strategy to overcome resistance to HER2-TKI therapy (Fig. 4G).

### AXL inhibition prevented the emergence and maintenance of HER2-inhibitor-tolerant cells

**Fig. 5:**
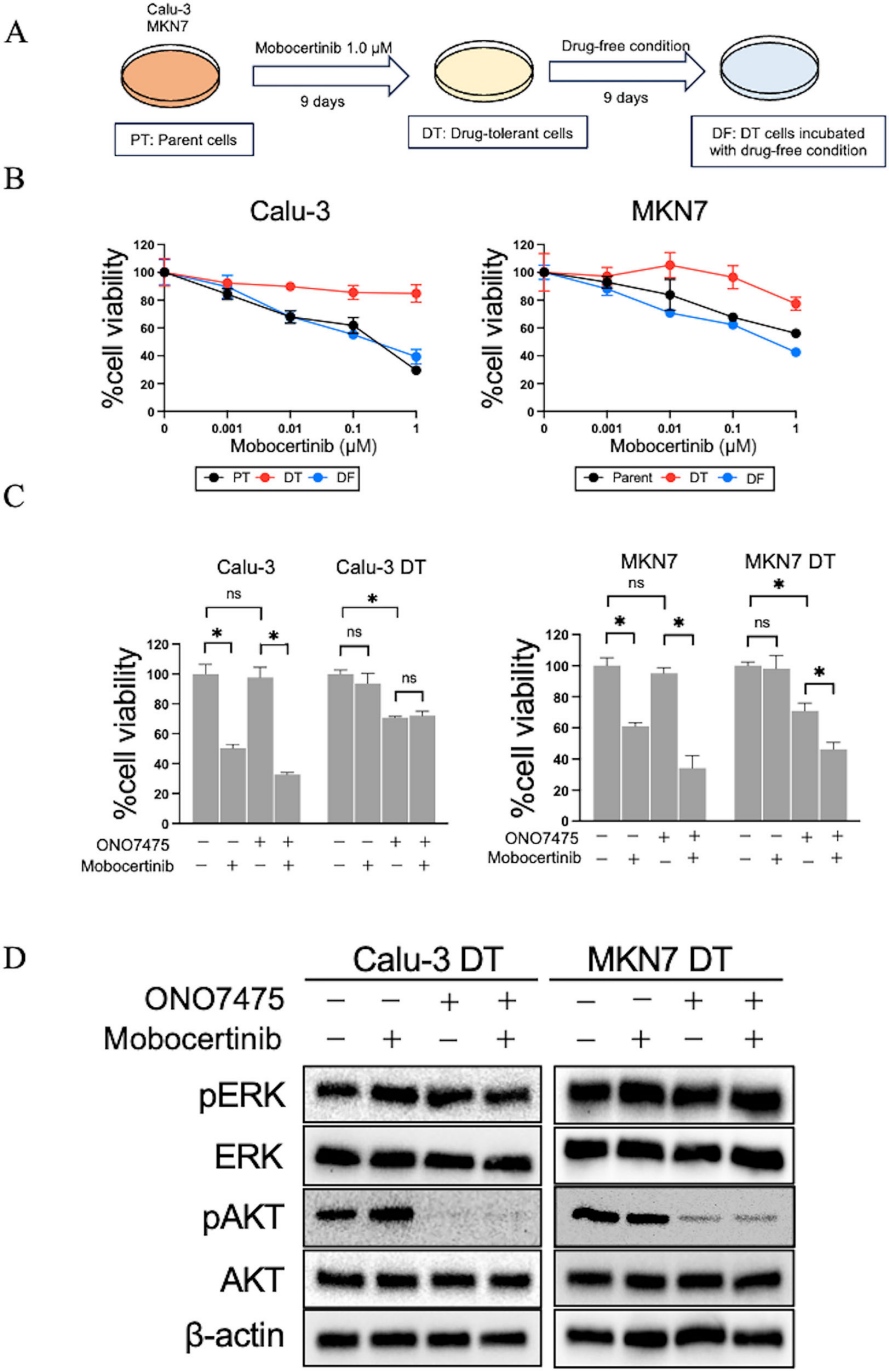
AXL inhibitor sensitized HER2-aberrant cancer cells with high AXL expression and prevented the emergence of drug tolerance. **A** Calu-3 and MKN7 cells were left untreated (left) or treated with mobocertinib (1.0 μmol/L) for 9 d (drug-tolerant (DT) cells; middle), and DT cells were incubated with a drug-free medium for 9 d (drug-free (DF) cells; right). **B** Calu-3 and MKN7 parental, DT, and DF cells were treated with the indicated concentration of mobocertinib for 72 h and assessed for cell viability using the MTT assay. **C** Calu-3 and MKN7 DT cells were incubated with mobocertinib (0.01 or 0.1 μmol/L), ONO7475 (0.3 μmol/L), or a combination of mobocertinib and ONO7475 for 72 h. Cell growth was determined using MTT assays. **D** Western blotting of Calu-3 and MKN7 DT cells treated with mobocertinib (0.01 or 0.1 μmol/L), ONO7475 (0.3 μmol/L), or a combination of mobocertinib and ONO7475 for 4 h.

To explore the role of AXL in drug tolerance, we investigated its effects on drug-tolerant (DT) cells, which are a small subpopulation of cells with significantly reduced sensitivity to targeted therapy^23^. DT cells were isolated from Calu-3 and MKN7 cell populations after 9 days of mobocertinib exposure (1.0 µM). Drug tolerance was confirmed to be reversible, as DT cells regained sensitivity after 9 days of drug-free culture, and are referred to as DF cells (Fig. 5A). Compared to parental or DF cells, DT cells exhibited slower growth (Supplementary Fig. 16A, B) and a larger proportion of Calu-3 and MKN7 DT cells were in the G1 phase of the cell cycle (Supplementary Fig. 16C, D). DT cells were more insensitive to mobocertinib than were parental or DF cells (Fig. 5B). Compared to mobocertinib monotherapy, treatment with the AXL inhibitor ONO7475 significantly decreased the viability of DT cells (Fig. 5C). Western blotting showed that while mobocertinib did not inhibit ERK or AKT phosphorylation in DT cells, ONO7475 suppressed AKT phosphorylation (Fig. 5D).

These findings indicate that AXL activation plays a critical role in both the emergence and maintenance of DT cells exposed to mobocertinib. Combining AXL inhibitors with HER2-TKIs may effectively suppress the formation of DT cells and enhance mobocertinib treatment efficacy.

### Baseline AXL expression was correlated with sensitivity to combined AXL and HER2-TKI Inhibition

To identify the factors contributing to HER2-TKI insensitivity, we analyzed the baseline expression of AXL, SHC1, and SHCBP1 in eight HER2-aberrant cancer cell lines. Among these, only Calu-3 (NSCLC) and MKN7 (gastric cancer) cells exhibited AXL expression, while the other six cell lines did not (Supplementary Fig. 17A). The AXL inhibitor ONO7475 significantly enhanced the efficacy of multiple HER2-TKIs, including mobocertinib, poziotinib, tucatinib, zongertinib, and sevabertinib, in Calu-3 and MKN7 cells. However, only marginal effects on the viability of the six cell lines lacking AXL expression were observed. These findings suggest that baseline AXL expression serves as a predictive biomarker for the insensitivity to HER2-TKIs (Supplementary Fig. 17B). To confirm the importance of baseline AXL expression, AXL-low-expressing NSCLC cell lines H2170 and H1781 were transfected with the AXL-WT plasmid to generate AXL-overexpressing cell lines. The overexpression of AXL induced the phosphorylation of AKT and ERK, a phenomenon which was not observed in the parental cells (Supplementary Fig. 18). These results indicate that baseline AXL expression is a predictive biomarker for the responsiveness to the combined inhibition of AXL and HER2-TKIs in HER2-aberrant cancer cells.

To investigate the clinical relevance of AXL expression, we analyzed tumor specimens obtained from patients with HER2-aberrant gastric and NSCLC cancers using immunohistochemistry. Among the five HER2-aberrant gastric tumor specimens, strong (3 + ), intermediate (2 + ), weak (1 + ), and negative (0) AXL expression was confirmed in 2 (40.0%), 2 (40.0%), and 1 (20.0%) cases, respectively, with no cases showing negative (0) expression. Among the 46 HER2-aberrant NSCLC tumor specimens, strong (3 + ), intermediate (2 + ), weak (1 + ), and negative (0) AXL expression was confirmed in 11 (23.9%), 20 (43.5%), 13 (28.3%), and 2 (4.3%) patients, respectively (Supplementary Fig. 19A, B). Overall, AXL 3+ was confirmed in 25.5% of the overall cohort of HER2-aberrant tumors, representing a relatively high prevalence for a biomarker.

This supports the potential clinical utility of targeting AXL in a substantial subset of patients, especially given the strong preclinical response to AXL-targeted combination therapy observed in AXL-positive models.

### Initial co-administration of ONO7475 and mobocertinib delayed tumor growth in cell-line-derived xenograft (CDX) models

**Fig. 6:**
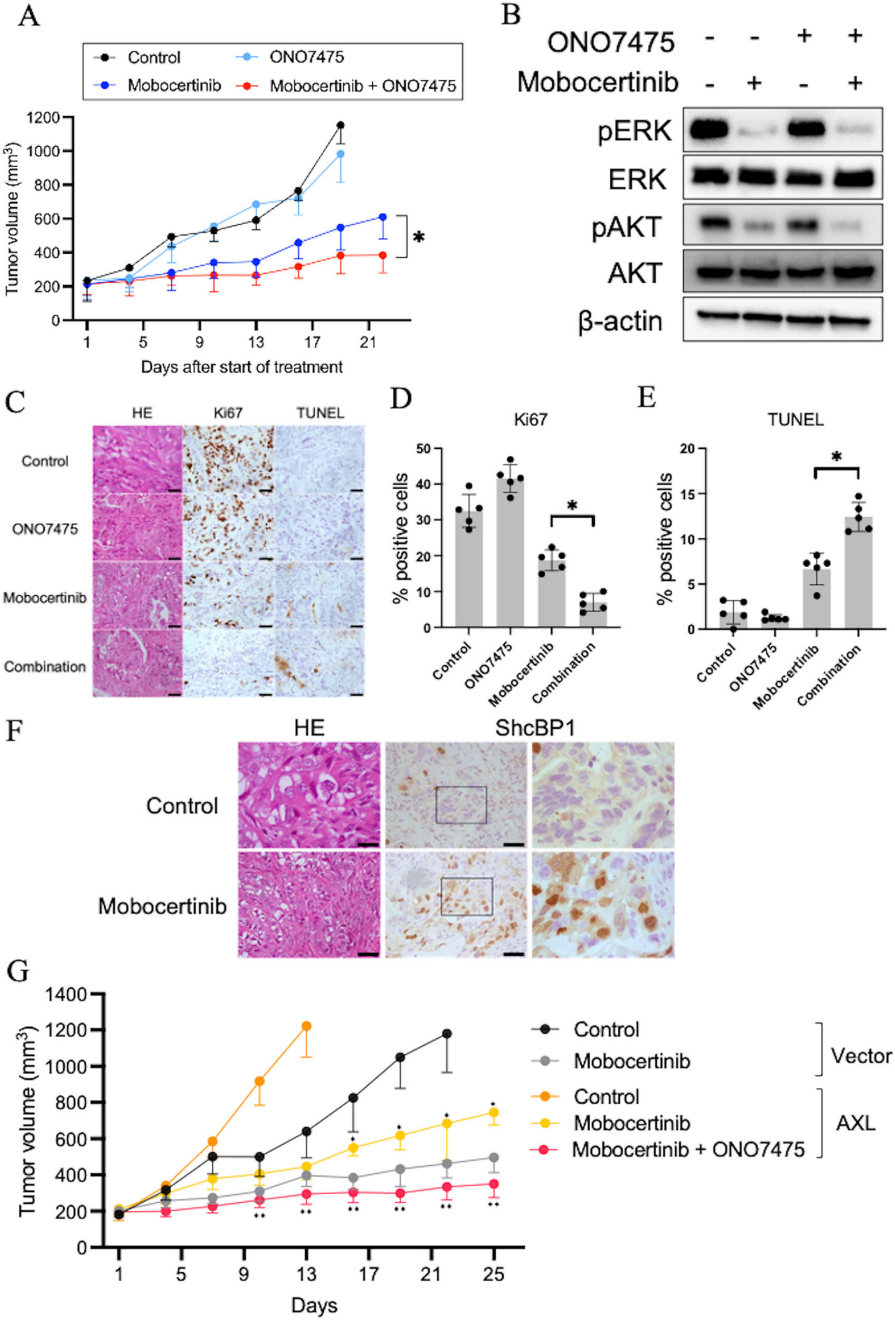
Initial combination therapy of ONO7475 and HER2-TKI delayed the regrowth of CDX tumors. **A** Calu-3 CDX tumors were treated with vehicle (control), ONO7475 10 mg/kg, mobocertinib 15 mg/kg, or ONO7475 10 mg/kg plus mobocertinib 15 mg/kg (*n* = 6, per group). Tumor volumes were measured over time, and the results are shown as the mean ± SEM. Statistical comparisons were conducted using two-way ANOVA. *P < 0.05. **B** Western blotting analysis of Calu-3 CDX tumors treated with mobocertinib (15 mg/kg), ONO7475 (10 mg/kg), or a combination thereof for 4 days. **C** Representative immunohistochemistry images of Calu-3 CDX tumors stained with hematoxylin and eosin (HE), specific human Ki-67 and TUNEL. Scale bar, 50 μm. Quantification of proliferating cells determined by **D** Ki67-positive proliferation index (percentage of Ki67-positive cells) and **E** TUNEL-positive proliferation index (percentage of TUNEL-positive cells) in Calu-3 CDX tumors. The columns represent the mean of five evaluated areas, and bars represent the standard deviation (SD). Statistical comparisons were conducted using one-way ANOVA. *P < 0.05. **F** Representative immunohistochemistry images of Calu-3 CDX tumors treated with vehicle (control) or mobocertinib 15 mg/kg for 4 days and stained with specific human ShcBP1 antibodies. Scale bar, 50 μm. **G** H2170 CDX tumors containing the transfected vector or overexpressing AXL were treated with vehicle (control) or mobocertinib 10 mg/kg (*n* = 6, per group) or ONO7475 10 mg/kg plus mobocertinib 10 mg/kg. Tumor volumes were measured over time, and the results are shown as mean ± SEM. Statistical comparisons were conducted using two-way ANOVA. *comparison of therapeutic efficacy of mobocertinib comparing tumors derived from vector-transfected or AXL-overexpressing H2170 cells; **comparison of therapeutic efficacy of the combination of mobocertinib and ONO7475 to mobocertinib monotherapy in AXL-overexpressing H2170 tumors.

We evaluated the therapeutic efficacy of combining ONO7475 with mobocertinib in a cell-line-derived xenograft (CDX) model using Calu-3 NSCLC cells. ONO7475 monotherapy did not affect tumor growth; however, the combination of ONO7475 with mobocertinib significantly reduced tumor size compared to mobocertinib monotherapy (Fig. 6A). Furthermore, analysis of tumor tissues collected on day 4 of treatment showed that combination treatment inhibited AKT phosphorylation more effectively than mobocertinib monotherapy (Fig. 6B). No adverse events, including weight loss, were observed during treatment (Supplementary Fig. 20A). Next, we assessed the pharmacological efficacy of therapy in CDX models established using Calu-3 cells. After 4 days of treatment, the number of Ki-67-positive proliferating tumor cells was significantly reduced in tumors treated with the combination therapy than that in those treated with either monotherapy (Fig. 6C, D). Additionally, TUNEL staining showed a higher apoptosis level in combination-treated tumors than that in those treated with monotherapy (Fig. 6C, E). Immunohistochemistry assays revealed that nuclear localization of SHCBP1 was enhanced in xenograft tumors treated with mobocertinib for day 4, compared to that in the vehicle-treated group. This finding is consistent with results from the cell-line-based analysis (Fig. 6F). Furthermore, to examine whether AXL expression levels influence the sensitivity of HER2-aberrant cancer cells to mobocertinib in vivo, we used H2170 NSCLC cells with sustained AXL expression induced by transfection with the AXL-WT plasmid. Sustained AXL protein expression was confirmed using western blotting in tumors derived from AXL-WT-transfected cells at 24 d post-inoculation (Supplementary Fig. 21). Tumors derived from AXL-overexpressing cells exhibited faster growth than those derived from vector-transfected cells. Although mobocertinib monotherapy demonstrated antitumor efficacy in vector-transfected tumors, its effect was significantly reduced in AXL-overexpressing tumors. However, the combination of mobocertinib and ONO7475 suppressed the growth of AXL-overexpressing tumors more effectively than mobocertinib monotherapy (Fig. 6G). No adverse events were observed in either vector-transfected or AXL-overexpressing H2170 xenografts during treatment (Supplementary Fig. 20B). These findings suggest that AXL activation reduces the therapeutic responsiveness to mobocertinib, contributes to drug tolerance, and supports tumor growth.

## Discussion

Previous studies have demonstrated that the development of molecularly targeted agents has significantly improved clinical outcomes for patients with advanced solid tumors harboring driver gene alterations. HER2-TKIs have emerged as pivotal therapeutic agents for the treatment of HER2-aberrant cancer. Recently, the FDA approved trastuzumab deruxtecan, an antibody–drug conjugate, for patients with unresectable or metastatic HER2-positive solid tumors^30^. Moreover, zongertinib, a novel HER2-TKI, has also shown promising clinical outcomes^31^.

However, the antitumor efficacy of HER2-TKIs remains limited due to insufficient cell sensitivity and the development of drug resistance^32,33^. The mechanisms of resistance to HER2-TKIs have been attributed to HER2 amplification or mutation and activation of downstream MAPK and PI3K/AKT pathways^20,34^. Residual tumor cells that survive molecularly targeted therapies may accumulate diverse resistance mechanisms, including minor resistant subpopulations, changes in the tumor microenvironment, and reversible drug-tolerant states. These factors contribute to tumor heterogeneity and drug resistance. Despite these findings, strategies to prevent acquired resistance to HER2-TKIs and tumor progression due to epigenetic changes remain unclear. Furthermore, it is uncertain which tumor characteristics and resistance signals are most critical for cell survival. To our knowledge, this is the first study to reveal that adaptive resistance to HER2-TKIs in HER2-aberrant cancer cells, particularly in NSCLC and gastric cancer, is mediated by AXL activation. Additionally, we demonstrated that the combination of an AXL inhibitor with HER2-TKIs was highly effective during the initial treatment phase. Importantly, baseline AXL expression was identified as a predictive biomarker for the efficacy of this combination therapy. In our clinical cohort, strong AXL expression was observed in 25.5% of *HER2*-aberrant tumor specimens, representing a significant prevalence for a therapeutic target. These findings underscore the clinical relevance of targeting AXL in a substantial subset of patients and support the potential utility of AXL-directed combination therapy in *HER2*-aberrant cancers.

AXL, a tyrosine kinase receptor belonging to the TAM family, plays a critical role in adaptive and acquired resistance to various molecularly targeted therapies and chemotherapeutic agents^27,35–37^. Targeting the AXL pathway has shown promise as a therapeutic strategy to counteract tumor progression in several cancer types. Our preclinical studies demonstrated that the EGFR-TKI osimertinib activates AXL signaling by binding to EGFR and HER3 in EGFR-mutant NSCLC cells, thereby contributing to adaptive resistance^27^. Moreover, combining an AXL inhibitor with osimertinib or KRAS inhibitors has proven highly effective during the initial treatment phase in AXL-overexpressing EGFR- or KRAS-mutant NSCLC cells^28,38^. In this study, we showed that AXL was activated by mobocertinib and maintained cell survival by interacting with EGFR, HER2, and HER3 in HER2-aberrant cells. Notably, inhibition of AXL signaling preferentially suppressed phosphorylation of AKT at Ser473, mTOR at Ser2448, and p70S6K at Ser424, whereas phosphorylation at other AKT sites and downstream effectors remained unaffected. This suggests that AXL signaling selectively maintains specific nodes of the AKT/mTOR survival pathway rather than broadly activating downstream HER2 signaling.

In addition, we showed via CDX mouse models and cell-based analyses that the combination of HER2-TKIs with an AXL inhibitor was more effective than HER2-TKI monotherapy in the initial treatment phase of AXL-expressing HER2-aberrant tumors. These results suggest that the efficacy of HER2-TKIs can be further enhanced by targeting AXL-dependent pathways in AXL-expressing tumors during the initial phase of treatment. Based on our results, future clinical trials are warranted to explore novel therapeutic strategies and assess the effectiveness of combining HER2-TKIs with AXL inhibitors during the initial treatment phase for patients with HER2-aberrant cancers exhibiting high AXL expression.

Recently, research on adapter proteins as novel therapeutic targets has been progressing and gaining attention. Of them, SHCBP1, an SHC1-binding protein, modulates SHCBP1-mediated signaling pathways and plays a key role in tumorigenesis and progression in multiple cancer types^39–42^. SHCBP1 expression has been linked to poor survival in patients with NSCLC and breast cancer^43,44^. SHCBP1 is also highly expressed in gastric and esophageal squamous cell cancers, where it promotes tumor growth and invasion^39,45,46^. Moreover, SHCBP1 expression is strongly associated with HER2 expression in breast and gastric cancer and correlates with a poor prognosis in HER2-positive patients. A previous preclinical study reported that SHCBP1 expression is associated with poor therapeutic responses to the anti-HER2 antibody trastuzumab in patients with HER2-positive gastric cancer^39^.

This is the first report to show that HER2-TKI treatment in HER2-aberrant cancer triggers the dissociation of SHCBP1 from SHC1, thereby facilitating the binding of SHC1 to AXL and the nuclear translocation of SHCBP1.

We found that HER2-TKI treatment induces SHC1 phosphorylation, suggesting that this modification plays a pivotal role in mediating these protein–protein interactions. Furthermore, this AXL-driven nuclear translocation of SHCBP1 modulates cell cycle progression, thereby contributing to adaptive resistance. These findings highlight the importance of targeting the AXL–SHC1 pathway as a novel therapeutic strategy to overcome adaptive resistance to HER2-TKIs.

In summary, our study revealed that adaptive resistance to HER2-TKIs in HER2-aberrant lung and gastric cancers was driven by AXL signaling via the SHCBP1–SHC1 axis. Importantly, the initial combination of HER2-TKIs with AXL inhibitors effectively reduced adaptive tumor cell survival by enhancing apoptosis. Given that strong AXL expression was identified in 25.5% of clinical specimens, this strategy holds significant promise for a substantial subset of patients. These findings suggest that the combination of HER2-TKIs and ONO7475 during the initial treatment phase could improve clinical outcomes for patients with HER2-aberrant cancer exhibiting high AXL expression. Future clinical trials are essential to validate the safety and efficacy of this combination therapy in HER2-aberrant lung and gastric cancers.

## Methods

### Cell culture and reagents

Eight human HER2-aberrant cancer cell lines were used in this study, with their characteristics summarized in Supplementary Table 1. Three human NSCLC cell lines (H2170 [RRID: CVCL_1535], H1781 [RRID: CVCL_1494], and Calu-3 [RRID: CVCL_0609]) and three human breast cancer cell lines (HCC1954 [RRID: CVCL_1259], SKBR3 [RRID: CVCL_0033], and MDA-MB-453 [RRID: CVCL_0418]) were purchased from the American Type Culture Collection (ATCC, Manassas, VA, USA). Two gastric cancer cell lines, MKN7 (RRID: CVCL_1417) and NUGC4 (RRID: CVCL_3082), were obtained from the RIKEN Cell Bank (Tokyo, Japan). H2170, H1781, Calu-3, HCC1954, MKN7, and NUGC4 cell lines were cultured in RPMI 1640 medium. SKBR3 cells were cultured in McCoy’s 5 A medium. MDA-MB-453 cells were cultured in Leibovitz’s L-15 medium. All media were supplemented with 10% fetal bovine serum, penicillin (100 U/mL), and streptomycin (100 µg/mL). Cells were maintained in a humidified incubator at 37 °C with 5% CO2. All cell lines were passaged for less than 3 months before being replaced with frozen early-passage stocks. Cells were regularly tested for mycoplasma contamination using a MycoAlert Mycoplasma Detection Kit (Lonza, Basel, Switzerland). The HER2-TKIs mobocertinib, poziotinib, tucatinib, afatinib and sevabertinib, as well as the AXL inhibitors ONO7475 and NPS1034, were purchased from Selleck Chemicals (Houston, TX, USA). Zongertinib was purchased from MedChemExpress (Monmouth Junction, NJ, USA). Recombinant human GAS6 was obtained from R&D Systems (Minneapolis, MN, USA).

### Cell viability assay

Tumor cells (2–4 × 10^3^ cells/100 μL/well) were seeded in 96-well plates and treated with the indicated compounds for 72 h. Cell viability was assessed using MTT assays (Sigma-Aldrich, St. Louis, MO, USA), following the manufacturer’s instructions. The percentage of growth was determined relative to untreated controls. Experiments were repeated at least three times with quadruplicate samples. Absorbance was measured at 570 nm (test wavelength) and 630 nm (reference wavelength) using a microplate reader. Percentage growth was calculated relative to that of untreated controls. For long-term viability studies, cells were treated with the indicated agents for 2 weeks, with media and drugs replenished every 72 h.

### Drug combination assay and synergy analysis

To evaluate the combinatorial effect of HER2-TKI and the AXL inhibitor ONO7475, we conducted a two-dimensional drug combination assay using the MTT assay. Cells were seeded into 96-well plates and treated with mobocertinib and ONO7475 in an 8 × 8 dose matrix format, where each drug was serially diluted across the following concentrations: 0, 0.001, 0.003, 0.01, 0.03, 0.1, 0.3, and 1.0 μM. After 72 h of drug exposure, cell viability was assessed using the MTT assay according to the manufacturer’s protocol. The resulting viability data were used to generate synergy maps. Synergy scores were calculated using the SynergyFinder web application (https://synergyfinder.org/#!/dashboard), based on the Bliss independence model.

### siRNA transfection

Duplexed Silencer® Select siRNAs targeting AXL (s1845 and s1846), GAS6 (s5587 and s5588), Src homology 2 domain-containing transforming protein 1 (Shc1; s12811 and s12813), and SH2-domain binding protein 1 (ShcBP1; s36361), FYN (s5434 and s5436), SRC (s13414), PTPN11 (s11524 and s11526) and GRB2 (s6119 and s226232) were purchased from Invitrogen (Carlsbad, CA, USA). An additional SRC-targeting siRNA (sc-5266) was purchased from Santa Cruz Biotechnology (Dallas, TX, USA). Cells were transfected with these siRNAs using Lipofectamine RNAi-MAX (Invitrogen) according to the manufacturer’s instructions. Silencer® Select siRNA for negative control #1 (Invitrogen) was used as the scrambled control. AXL, Shc1, and ShcBP1 knockdown was confirmed using western blotting analysis. GAS6 knockdown was verified using real-time PCR. For siRNA screening, the Silencer® Select human kinase siRNA library V4 (Ambion, #4397918) was used following the manufacturer’s instructions.

### Western blotting

Protein samples (25 μg per lane) were separated using SDS-PAGE (Bio-Rad, Hercules, CA, USA) and transferred onto polyvinylidene difluoride membranes (Bio-Rad). Membranes were washed three times with Tris-buffered saline, blocked using a blotting-grade blocker (Bio-Rad) for 1 h at 25 °C, and incubated overnight at 4 °C with primary antibodies (listed in Supplementary Table 2). After three washes with Tris-buffered saline, membranes were incubated with HRP-conjugated species-specific secondary antibodies for 1 h at 25 °C. Immunoreactive bands were visualized using SuperSignal West Dura Extended Duration substrate (Thermo Fisher Scientific, Waltham, MA, USA). Densitometric analysis of the bands was performed using ImageJ software (National Institutes of Health, Bethesda, Maryland, USA). Band intensities of p-AKT were normalized to the loading control (β-actin) to calculate the p-AKT/β-actin ratio. All of the uncropped western blots with molecular weight indicated were presented in Supplementary Fig. 22. All experiments were independently repeated at least three times.

### Cell cycle and apoptosis analysis

For cell cycle analysis, 1 × 10^5^ cells were harvested 48 h after treatment with mobocertinib (0.01 or 0.1 µmol/L) and/or ONO7475 (0.3 µmol/L), or 72 h after treatment with mobocertinib (0.01 or 0.1 µmol/L) and/or ShcBP1 knockdown. Cells were washed twice with ice-cold phosphate-buffered saline (PBS), pelleted via centrifugation, and resuspended at 1 × 10^6^ cells/mL in propidium iodide staining buffer (0.1% Triton X-100 and 50 µg/mL propidium iodide in PBS). For apoptosis analysis, 1 × 10^5^ cells were treated with or without mobocertinib (0.01 or 0.1 µmol/L) and/or ONO7475 (0.3 µmol/L) for 48 h. Cells were washed twice with ice-cold PBS and stained with annexin V-fluorescein isothiocyanate (Annexin V-FITC) and propidium iodide for 15 min at 25 °C in the dark. Both floating and adherent cells were collected, and a minimum of 1 × 10^4^ events per culture were analyzed using a BD Accuri C6 Plus flow cytometer (Becton, Dickinson & Company, Franklin Lakes, NJ, USA). Data were analyzed using FlowJo® software (RRID: SCR_008520, FlowJo LLC, Ashland, OR, USA), as reported previously^47^.

### Real-time PCR analysis

Total RNA was extracted using NucleoSpin® RNA Plus (Takara Bio, Shiga, Japan), and cDNA was synthesized using the PrimeScript™ RT master mix (Perfect Real Time; Takara Bio) following the manufacturer’s instructions. Real-time PCR was performed using a TaKaRa PCR thermal cycler Dice® (Takara Bio) and SYBR Fast qPCR kit (Kapa Biosystems, Cape Town, South Africa) under the following conditions: initial incubation at 95 °C for 10 min, 40 cycles at 95 °C for 15 s, and 60 °C for 1 min. Melting curve analysis was performed to confirm primer specificity. Gene expression levels were calculated from relative standard curves, normalized to GAPDH levels, and analyzed using the 2^−ΔΔCT^ method. Primer sequences used in this study are listed in Supplementary Table 3.

### Immunoprecipitation

Cells were lysed in immunoprecipitation buffer (50 mM Tris-HCl, pH 7.4, 150 mM NaCl, 1% NP-40, 1 mM EDTA) supplemented with protease and phosphatase inhibitors (Roche). Lysates were incubated with primary antibodies overnight at 4 °C, followed by incubation with Protein G magnetic beads (Thermo Fisher Scientific) for 2 h. The beads were washed, and bound proteins were eluted using SDS sample buffer and analyzed by western blotting.

### Nuclear and cellular protein fractions

Nuclear and cytoplasmic protein fractions were extracted using NE-PER™ Nuclear and Cytoplasmic Extraction Reagents (Thermo Fisher Scientific) according to the manufacturer’s protocol. Cells were harvested, washed with PBS, and lysed to separate cytoplasmic and nuclear components. Protein concentrations were measured using the BCA assay (Thermo Fisher Scientific) and analyzed by western blotting.

### Plasmid construction

pWPXL plasmids encoding either an empty vector (Vector) (RRID: Addgene_12257) or wild-type human AXL (AXL-WT) (RRID: Addgene_65627) were purchased from Addgene (Watertown, MA, USA). H1781 and H2170 cells were transfected with the Vector or AXL-WT plasmids using the X-tremeGene HP DNA Transfection Reagent (Roche) following the manufacturer’s instructions. Whole-cell lysates were analyzed using western blotting to confirm successful transfection.

### Immunofluorescence microscopy

Cells were cultured on 12-mm diameter Transwell filter inserts with a 0.4-μm pore size (Corning, Munich, Germany) for 6 days. Cells were fixated with 3.7% formaldehyde at room temperature for 20 min, permeabilized using a solution of 0.2% (w/v) Triton X-100 (Nacalai, Kyoto, Japan) for 60 min, and blocked with 2% bovine serum albumin in PBS for 60 min. Then, cells were incubated with a primary antibody for 60 min, followed by incubation with a fluorescently labeled secondary antibody. Antibodies and their dilutions are listed in Supplementary Table 2. Fluorescent images were captured using a confocal microscope (LSM900, Zeiss).

### Cell-line-derived xenograft (CDX) models

Calu-3 cells (4 × 10⁶ cells), H2170 cells (5 × 10⁶ cells), vector-transfected H2170 cells, and AXL-overexpressing H2170 cells (5 × 10⁶ cells each) were suspended in PBS and subcutaneously injected into the flanks of 5-week-old male CB-17/Icr-scid/scidJcl mice with severe combined immunodeficiency (Clea Japan, Tokyo, Japan). When the mean tumor volume reached approximately 150–250 mm3, mice were treated with the indicated drugs via daily oral gavage. Body weight was monitored 2–3 times weekly, and the general condition of the mice was assessed daily. Tumors were measured twice weekly using calipers, and tumor volume was calculated as: (width^2^ × length)/2. All animal experiments were conducted in accordance with protocols approved by the Institutional Review Board of the Kyoto Prefectural University of Medicine (Kyoto, Japan; approval no. M29–529). Animal surgeries were performed under sodium pentobarbital anesthesia according to institutional guidelines, with all efforts made to minimize animal suffering.

### Human tumor specimens and immunohistochemistry

Tumor specimens were obtained from patients with HER2-aberrant NSCLC or gastric cancer at Kyoto Prefectural University of Medicine (Kyoto, Japan), Fujita Health University School of Medicine (Aichi, Japan), St. Marianna University School of Medicine (Kawasaki, Japan), Nagasaki University Graduate School of Biomedical Sciences (Nagasaki, Japan), Tokushima University (Tokushima, Japan), Fukuchiyama City Hospital (Kyoto, Japan), and Uji Tokushukai Medical Center (Kyoto, Japan). AXL expression was assessed by immunohistochemistry using formalin-fixed, paraffin-embedded tumor sections. AXL expression levels were evaluated independently by a pathologist and scored as strong (3 + ), intermediate (2 + ), weak (1 + ), or negative (0), with vascular endothelial cells as internal positive controls, as previously described^27^. This study was conducted in accordance with the Declaration of Helsinki. An opt-out method was utilized at each hospital. This study was approved by the institutional review board of the University Hospital, Kyoto Prefectural University of Medicine (approval No. ERB-C-3617) and those of each hospital.

### Tumor histological analyses

Formalin-fixed, paraffin-embedded tissue sections (4 μm thick) were deparaffinized and underwent antigen retrieval by microwaving in 10 mmol/L citrate buffer (pH 6.0). Tumor proliferation was evaluated using a Ki-67 antibody (RRID: AB_2687921, Clone MIB-1; Dako, Agilent, Santa Clara, CA, USA) with the EnVision+ System-HRP (DAB) kit (Agilent) according to the manufacturer’s protocol. After antigen retrieval, sections were incubated with the Ki-67 antibody at 4 °C overnight in a humidified chamber. Apoptosis was quantified using the terminal deoxynucleotidyl transferase-mediated dUTP-biotin nick-end labeling (TUNEL) method with the In Situ Cell Death Detection Kit, POD (Merck KGaA, Darmstadt, Germany), following the manufacturer’s instructions. For histological quantification, five fields (0.1 mm²/section) containing the most positively stained cells were analyzed at 400× magnification using light microscopy. Both the intensity and percentage of positively stained cells were recorded.

### Statistical analysis

Data from MTT assays and tumor progression in xenografts are expressed as mean ± standard deviation (SD) and means ± standard error (SE), respectively. Statistical significance was assessed using one analysis of variance (ANOVA), two-way ANOVA, or unpaired t-tests as appropriate. All statistical analyses were performed using GraphPad Prism Ver. 8.0 (GraphPad Software, Inc., San Diego, CA, USA). A two-sided *P*-value < 0.05 was considered statistically significant.

## Supplementary information

41698_2026_1385_MOESM1_ESM

## Data Availability

Qualified researchers can apply for access to our data by signing a data usage agreement. Other data supporting the findings of this study are available upon reasonable request from the corresponding author.
