# Supplementary material for: AXL–SHC1 signaling axis mediates adaptive resistance to HER2-targeted tyrosine kinase inhibitors in HER2-aberrant lung and gastric cancers
Source: NPJ Precis Oncol. 2026 Apr 2;10:142. doi: 10.1038/s41698-026-01385-2 (PMC13046926; doi:10.1038/s41698-026-01385-2)

Supplementary Table 1.
Characteristics of cell lines utilized in this study.

Cell lines	Type of cancers	Status of HER2 aberration
H2170	Lung	HER2 amplification
H1781	Lung	HER2 mutation
Calu-3	Lung	HER2 amplification
HCC1954	Breast	HER2 amplification
SKBR3	Breast	HER2 amplification
MDA-MB-453	Breast	HER2 mutation
MKN7	Stomach	HER2 amplification
NUGC4	Stomach	HER2 amplification

Supplementary Table 2.
Details of the antibodies utilized in this study.

Antibodies	Dilution	Company	Catalog#
p-AXL (Tyr702)	1 : 1000	Cell Signaling Technology	5724
AXL (C44G1)	1 : 1000	Cell Signaling Technology	4566
t-EGFR	1 : 1000	R&D Systems	AF231
HER2	1 : 1000	Cell Signaling Technology	2165
pHER2	1 : 1000	Cell Signaling Technology	2243
HER3	1 : 1000	Cell Signaling Technology	12708
p-AKT (Ser473)	1 : 1000	Cell Signaling Technology	4060
p-AKT (Thr308)	1 : 1000	Cell Signaling Technology	13038
p-AKT (Tyr326)	1 : 1000	Cell Signaling Technology	2968
t-AKT	1 : 1000	Cell Signaling Technology	9272
p-ERK1/2 (Thr202/Tyr204)	1 : 1000	Cell Signaling Technology	4370
t-ERK1/2	1 : 1000	Cell Signaling Technology	4695
p-SHC1 (Tyr239/240)	1 : 1000	Cell Signaling Technology	2434
SHC1	1 : 1000	Santa Cruz Biotechnology	Sc-967
SHCBP1	1 : 1000	Proteintech	12672-1-AP
p-mTOR (Ser2448)	1 : 1000	Cell Signaling Technology	5536
mTOR	1 : 1000	Cell Signaling Technology	2972
Phospho-p70 S6 Kinase (Ser424)	1 : 1000	Cell Signaling Technology	9204
Phospho-p70 S6 Kinase (Thr389)	1 : 1000	Cell Signaling Technology	9205
p70 S6 Kinase	1 : 1000	Cell Signaling Technology	9202
HDAC2	1 : 1000	Santa Cruz Biotechnology	sc-9959
GAPDH	1 : 1000	Cell Signaling Technology	2118
β-actin	1 : 1000	Cell Signaling Technology	4970

Supplementary Table 3.

Primer sequences for PCR utilized in this study.

Gene	Primer sequence
GAS6	Forward: 5′- ACATCTTGCCGTGCGTGCCCTTCA-3′
	Reverse: 5′- ATTCCGCGCCAGCTCCTCAACAGA-3′
GAPDH	Forward: 5′- GTCTCCTCTGACTTCAACAGCG-3′
	Reverse: 5′- ACCACCCTGTTGCTGTAGCCAA-3′

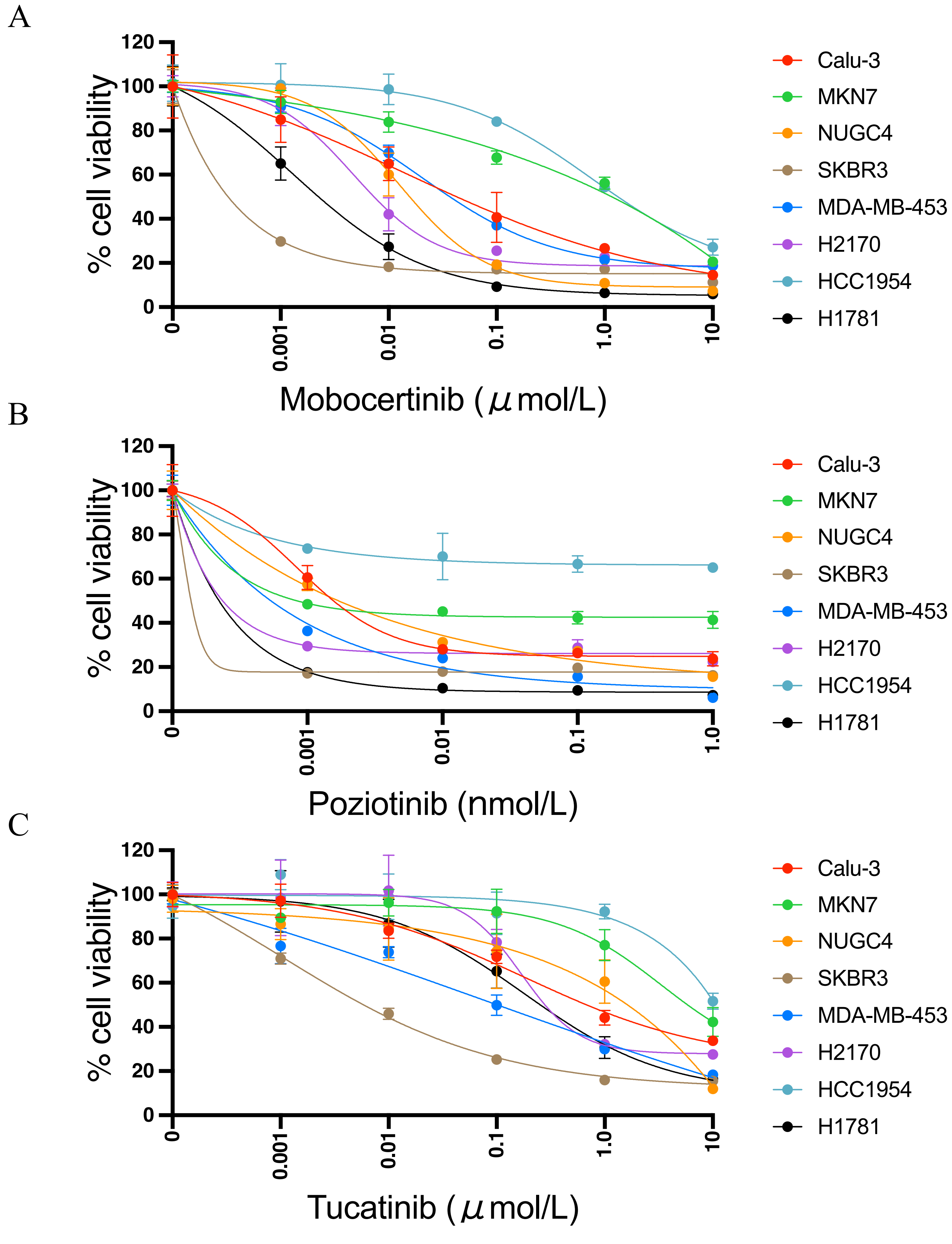

Supplementary Figure 1. **Cell viability after treatment with HER2-TKIs.**
Cell lines were treated for 72 h with varying doses of (A) mobocertinib, (B) poziotinib, or (C) tucatinib. Cell viability was measured using the MTT assay.

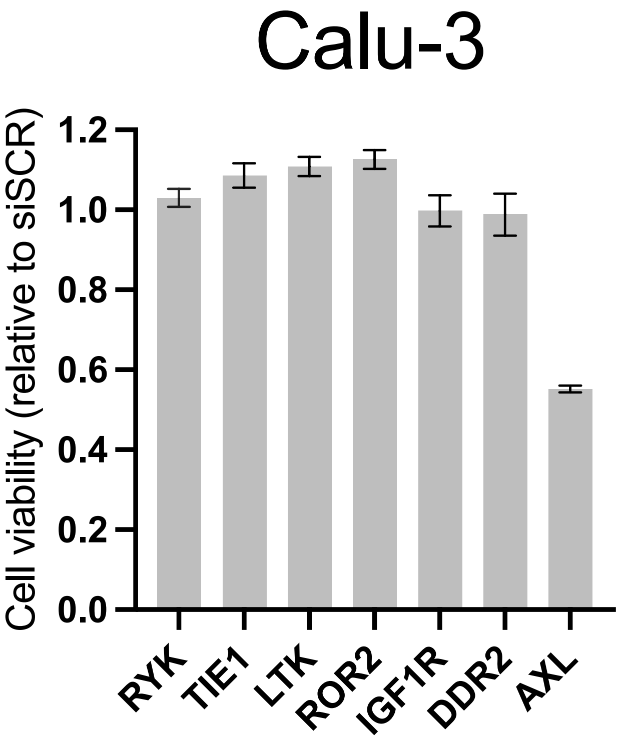

Supplementary Figure 2. Combination of knockdown of other genes that showed stronger inhibitory activity than AXL gene in mobocertinib in MKN7 cells was also evaluated in Calu-3 cells.
Calu-3 cells treated with RYK, TIE1, LTK, ROR2, IGF1R, DDR2, or AXL specific siRNAs were incubated with mobocertinib (0.01 μmol/L) for 72 h and cell viability was measured using the MTT assay.

A

Calu-3

Type of drugs	siSCR		siAXL	
	-	+	-	+
Mobocertinib	1.14	0.53	0.98	0.20
Poziotinib	1.25	0.41	0.88	0.09
Tucatinib	1.00	0.54	0.95	0.26

B

MKN7

Type of drugs	siSCR		siAXL	
	-	+	-	+
Mobocertinib	1.83	1.18	1.99	0.70
Poziotinib	1.29	0.72	1.11	0.10
Tucatinib	1.11	0.66	0.75	0.13

Supplementary Figure 3. Quantification of pAKT/β-actin ratio in Calu-3 and MKN7 cells following AXL knockdown and HER2-TKI treatment.

Densitometric quantification of p-AKT normalized to β-actin in **(A)** Calu-3 and **(B)** MKN7 cells transfected with nonspecific control or AXL-specific siRNAs. p-AKT/β-actin ratios were calculated using Image J .

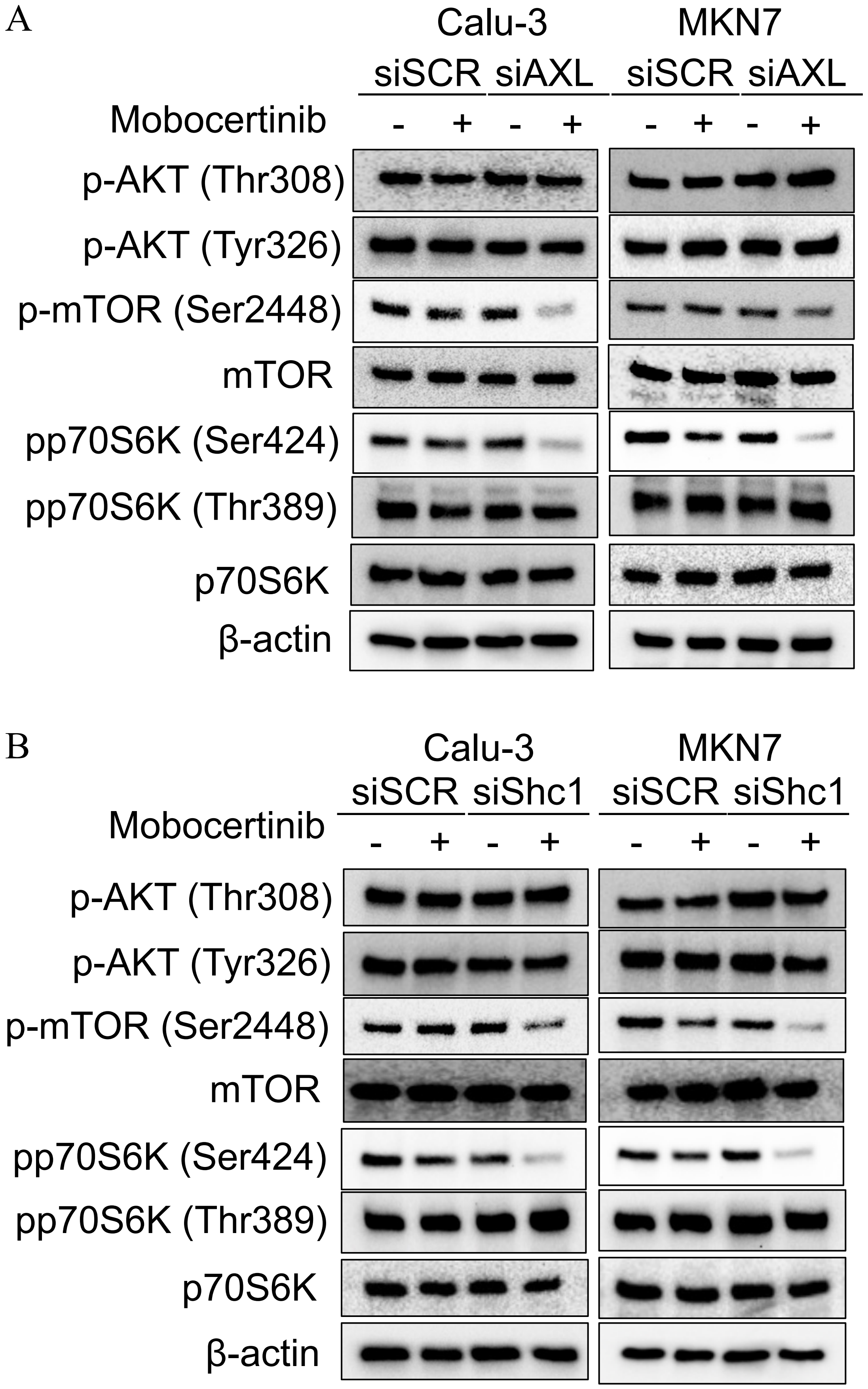

Supplementary Figure 4. Effects of AXL or Shc1 knockdown on AKT/mTOR/p70S6K signaling in HER2-aberrant cancer cells treated with mobocertinib at 4h.
Calu-3 and MKN7 cells were transfected with nonspecific control or **(A)** AXL or **(B)** Shc1-specific and treated with or without mobocertinib (0.01 or 0.1 μmol/L). Phosphorylation levels of AKT (Thr308, Tyr326), mTOR (Ser2448), and p70S6K (Ser424, Thr389) were evaluated by western blotting.

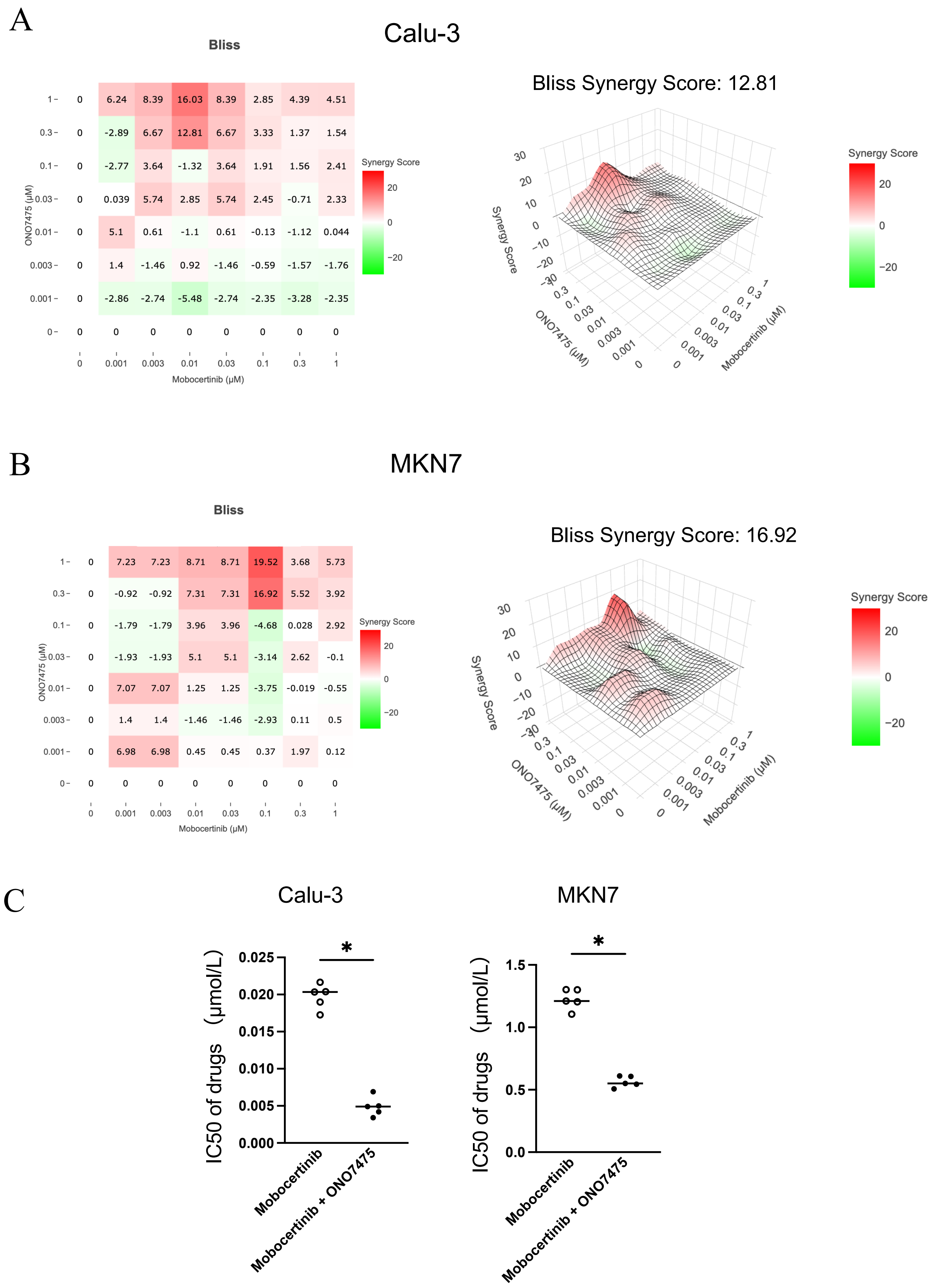

Supplementary Figure 5. Synergistic effect of mobocertinib and ONO7475 in Calu-3 and MKN7 cells. Heatmaps and 3D surface plots showing Bliss synergy scores for mobocertinib and ONO7475 across an 8×8 dose matrix (0–1.0 μM) in **(A)** Calu-3 and **(B)** MKN7 cells.

(C) IC_{50} values of mobocertinib (0.01 or 0.1 μmol/L) with or without ONO7475 (0.3 μmol/L) were determined using an MTT assay.

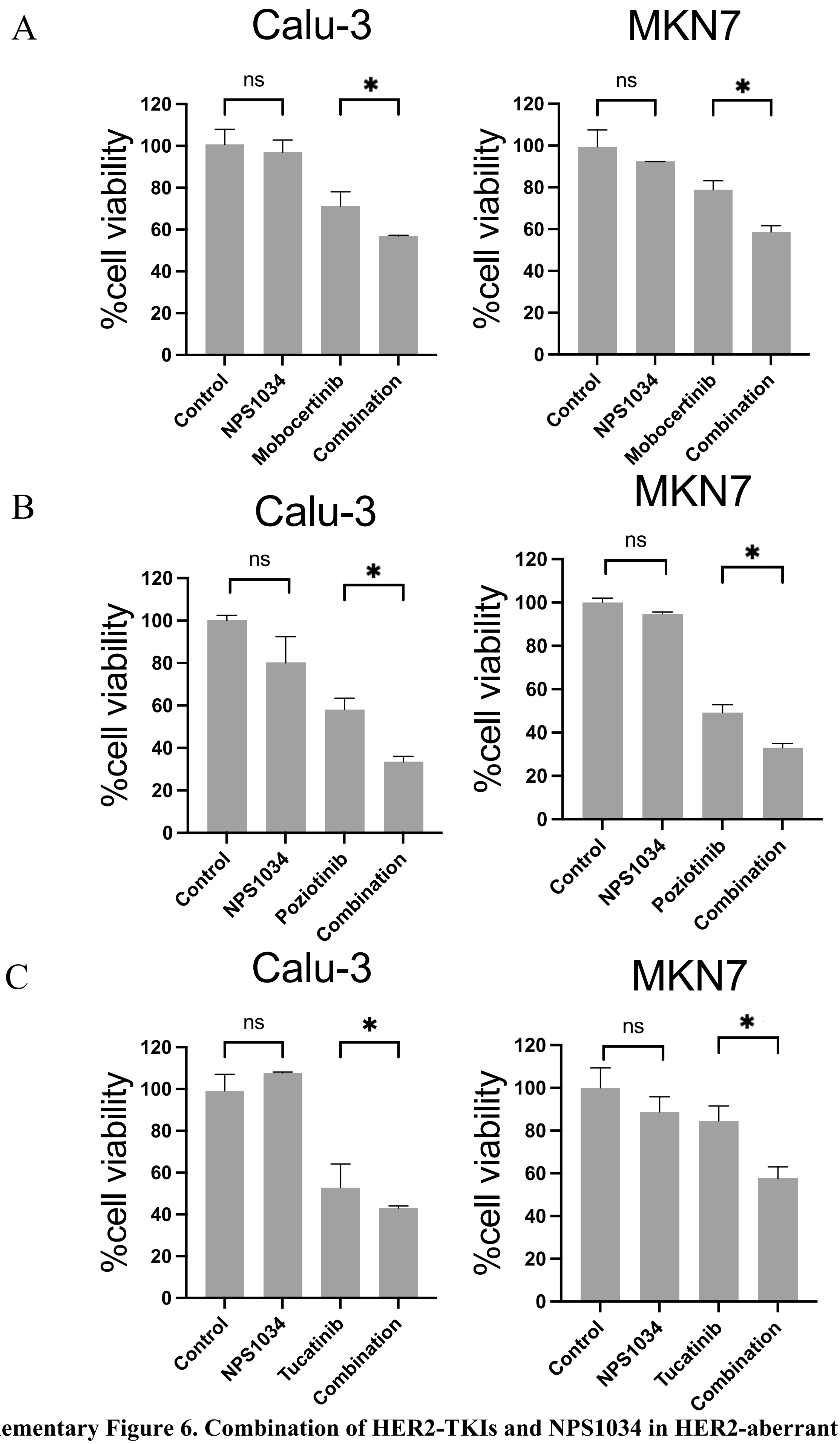

Supplementary Figure 6. Combination of HER2-TKIs and NPS1034 in HER2-aberrant tumor cells. The two cell lines were treated with NPS1034 (0.3 μmol/L) or (A) mobocertinib (0.01 or 0.1 μmol/L), (B) poziotinib (0.01 or 0.1 nmol/L), (C) tucatinib (0.1 or 1.0 μmol/L), or a combination of all these agents for 72 h, and the cell viability was assessed using an MTT assay (n = 4, mean ± SD, *$P < 0.05$) .

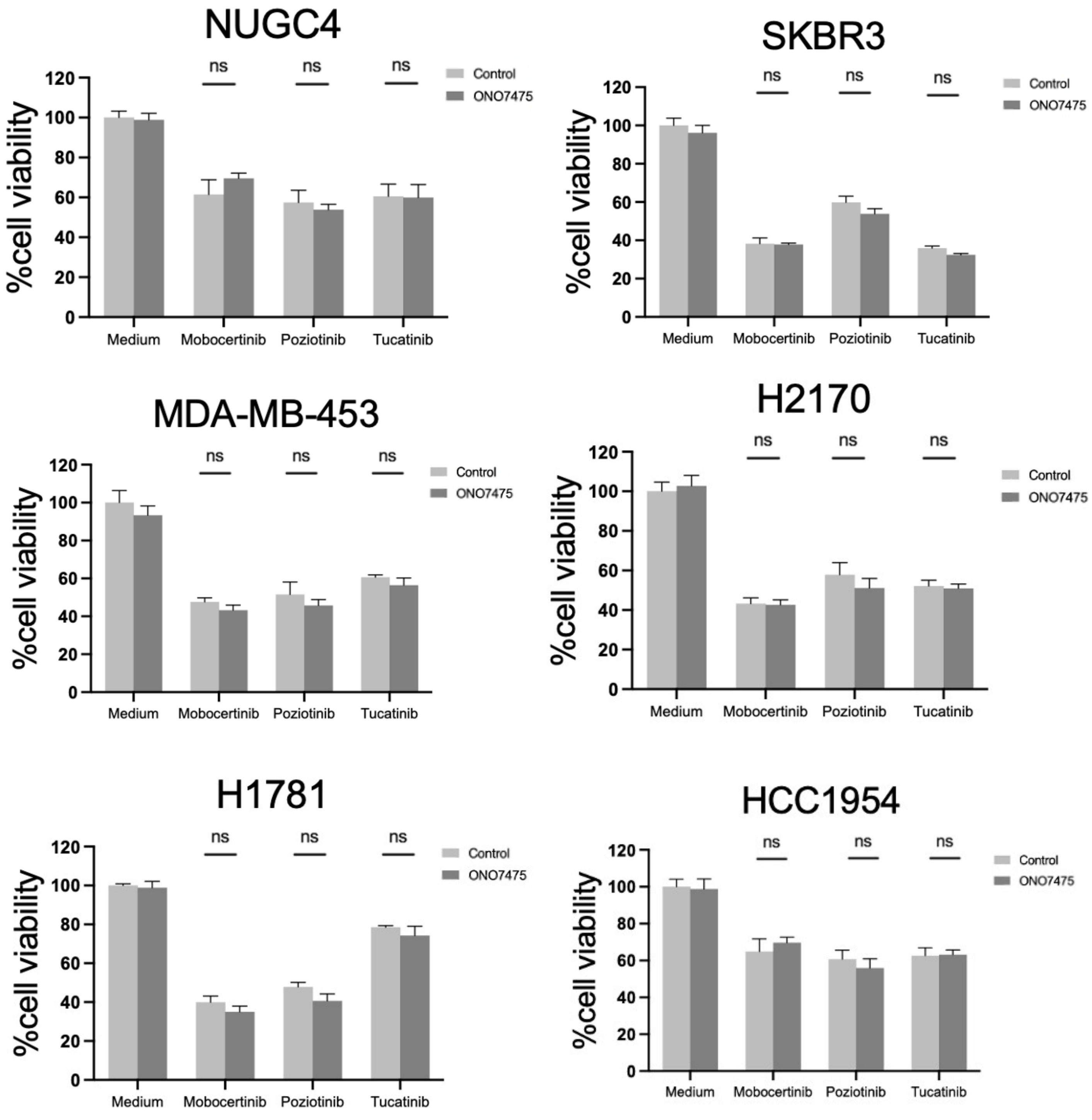

Supplementary Figure 7. Combination of HER2-TKIs and ONO7475 in HER2-aberrant tumor cells. The six cell lines were treated with ONO7475 (0.3 μmol/L) or mobocertinib (0.01 or 0.1 μmol/L) or poziotinib (0.01 or 0.1 nmol/L) or tucatinib (0.1 or 1.0 μmol/L), or a combination of all these agents for 72 h, and the cell viability was assessed using an MTT assay (n = 4, mean ± SD, *****$P < 0.05$).

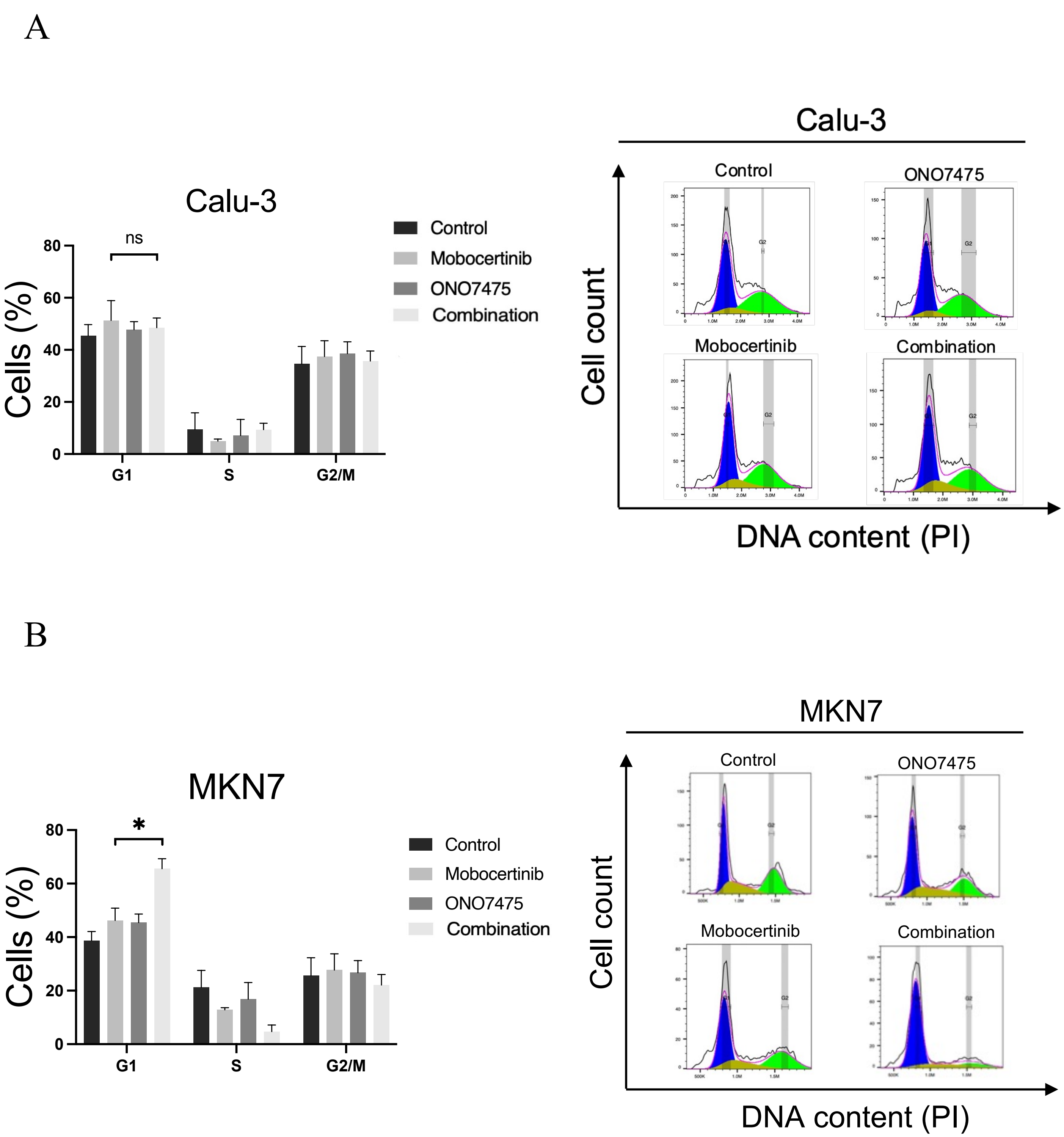

Supplementary Figure 8. Cell cycle analysis in HER2-aberrant tumor cells.
The cell cycle was analyzed via flow cytometry with propidium iodide (PI) following treatment with mobocertinib (0.01 or 0.1 μmol/L) with or without ONO7475 (0.3 μmol/L) in (A) Calu-3 and (B) MKN7 cells.

A

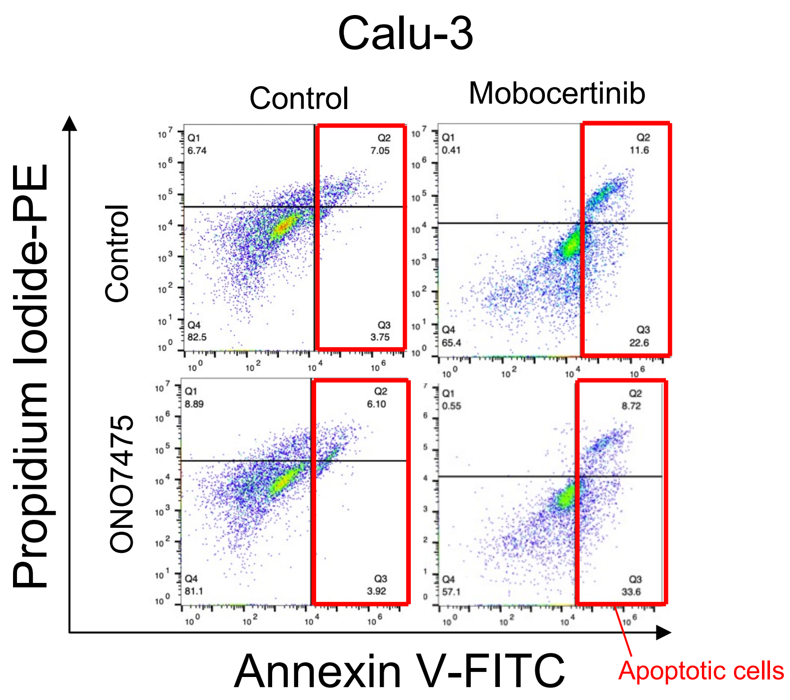

B

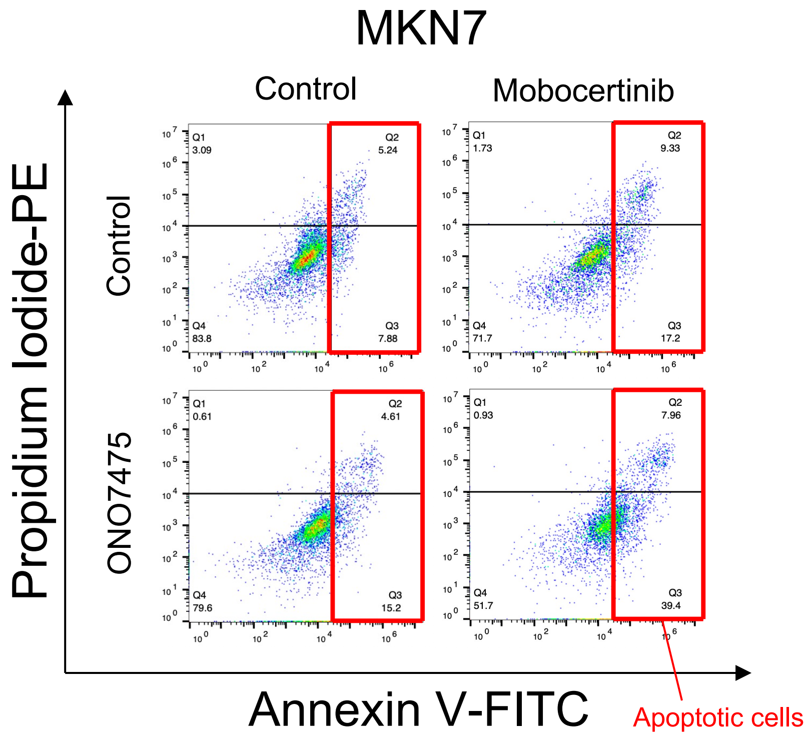

Supplementary Figure 9. Compared to ONO7475 alone, the combination of mobocertinib with ONO7475 enhances apoptosis in HER2-aberrant tumor cells.
Apoptotic cells in (A) Calu-3 and (B) MKN7 were detected by flow cytometry following treatment with medium, mobocertinib (0.01 or 0.1 μmol/L), ONO7475 (0.3 μmol/L), or the combination of mobocertinib with ONO7475 (0.3 μmol/L) for 48 h. Representative figures showing the population of live cells (annexin V–/ PI–), early apoptotic (live) cells (annexin V+/ PI-), late apoptotic (dead) cells (annexin V+/PI+), and necrotic (annexin V–/PI+) cells.

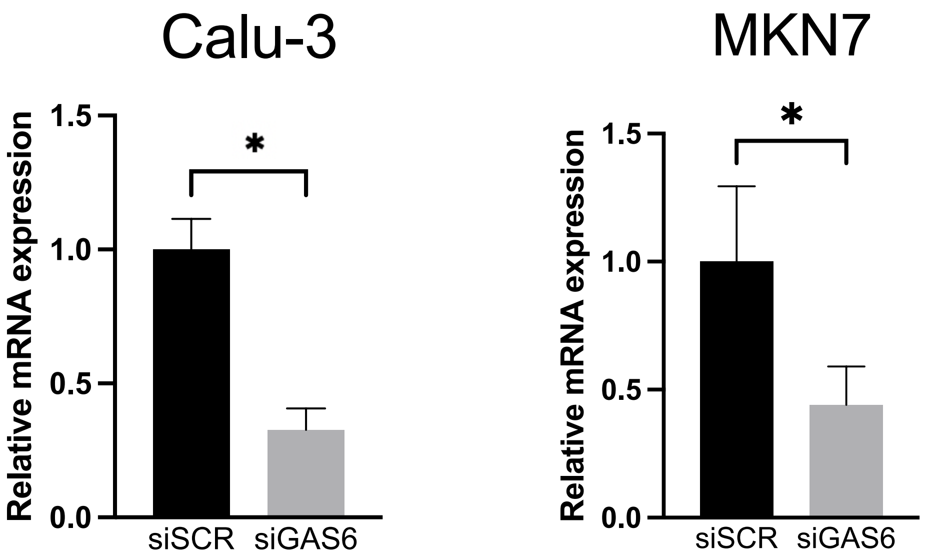

Supplementary Figure 10. qPCR analysis of GAS6 expression in Calu-3 and MKN7 parent cells. Cells were treated with nonspecific control or GAS6-specific siRNAs for 24 h. *$P < 0.05$ (unpaired *t*-test).

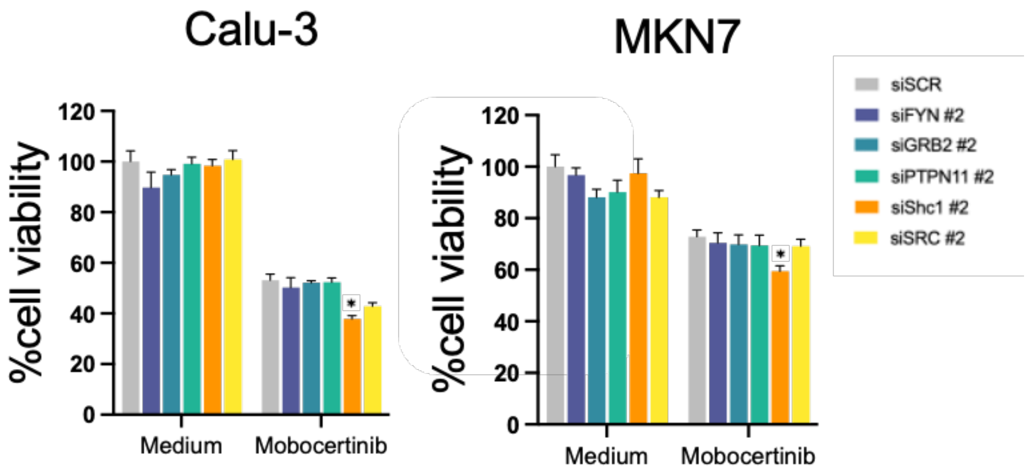

Supplementary Figure 11. Effect of adaptor protein knockdown on mobocertinib-induced growth inhibition in Calu-3 and MKN7 cells.

The combination of mobocertinib and knockdown of several adaptor proteins in Calu-3 and MKN7 cells. Calu-3 and MKN7 cells treated with nonspecific control, FYN, GRB2, PTPN11, Shc1, or SRC-specific siRNAs were incubated with or without mobocertinib (0.01 or 0.1 μmol/L) for 72 h and cell viability was assessed using an MTT assay. *$P < 0.05$ compared with nonspecific control siRNA (two-way ANOVA). Data are represented as the mean ± SD.

A

Calu-3

Mobocertinib	siSCR		siShc1	
	-	+	-	+
pAKT	1.11	0.36	1.02	0.19
pERK	1.49	0.09	1.19	0.06

B

MKN7

Mobocertinib	siSCR		siShc1	
	-	+	-	+
pAKT	1.63	0.67	1.61	0.33
pERK	1.26	0.25	1.96	0.27

Supplementary Figure 12. Quantification of pAKT/β-actin and pERK/β-actin ratios in Calu-3 and MKN7 cells following Shc1 knockdown and HER2-TKI treatment.
Densitometric quantification of pAKT and pERK levels normalized to β-actin in **(A)** Calu-3 and **(B)** MKN7 cells transfected with either nonspecific control siRNA or Shc1-specific siRNAs. The pAKT/β-actin and pERK/β-actin ratios were calculated using ImageJ software.

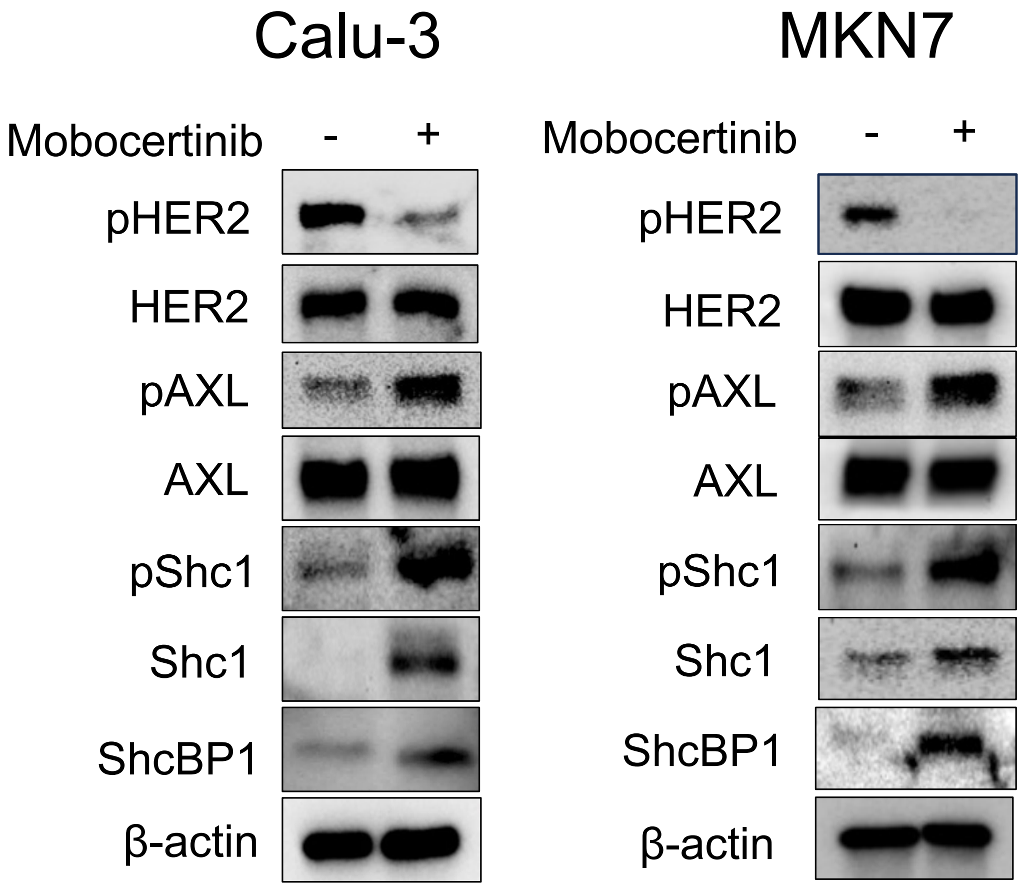

Supplementary Figure 13. Expression of phosphorylated and HER2, AXL, Shc1 in Calu-3 and MKN7 cells treated with or without mobocertinib.
Calu-3 and MKN7 cells were treated with or without mobocertinib (0.01 or 0.1 μmol/L) for 24 h. Phosphorylated and of HER2, AXL, and Shc1 were detected using western blotting.

A

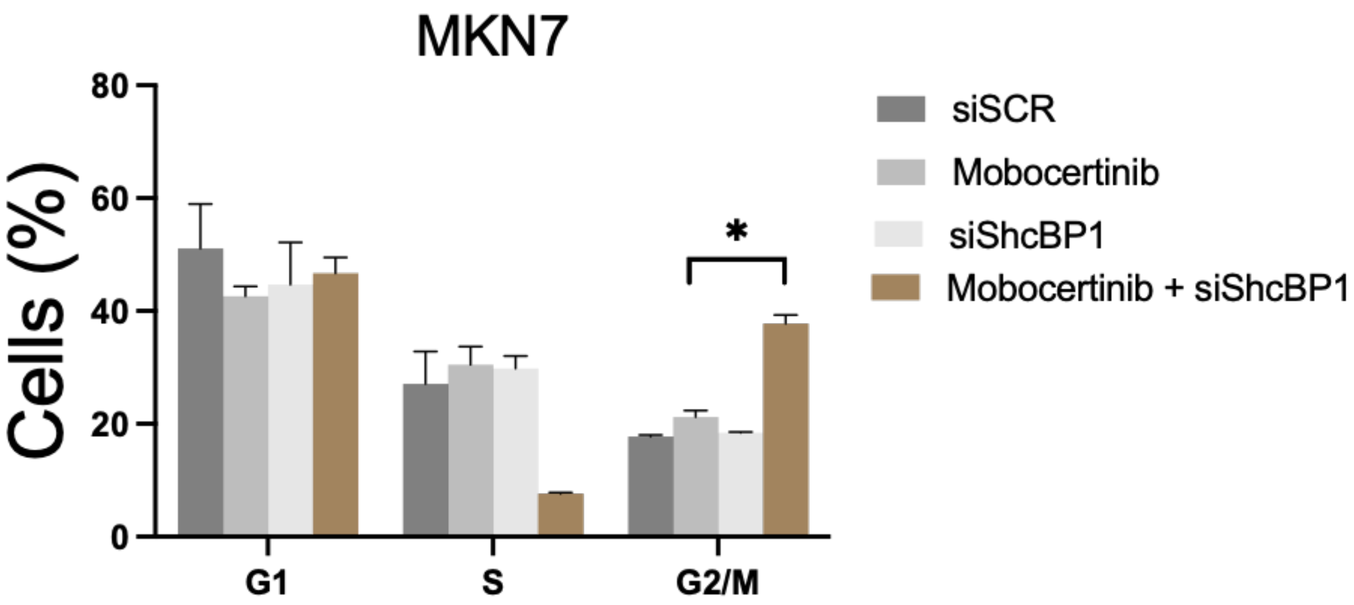

B

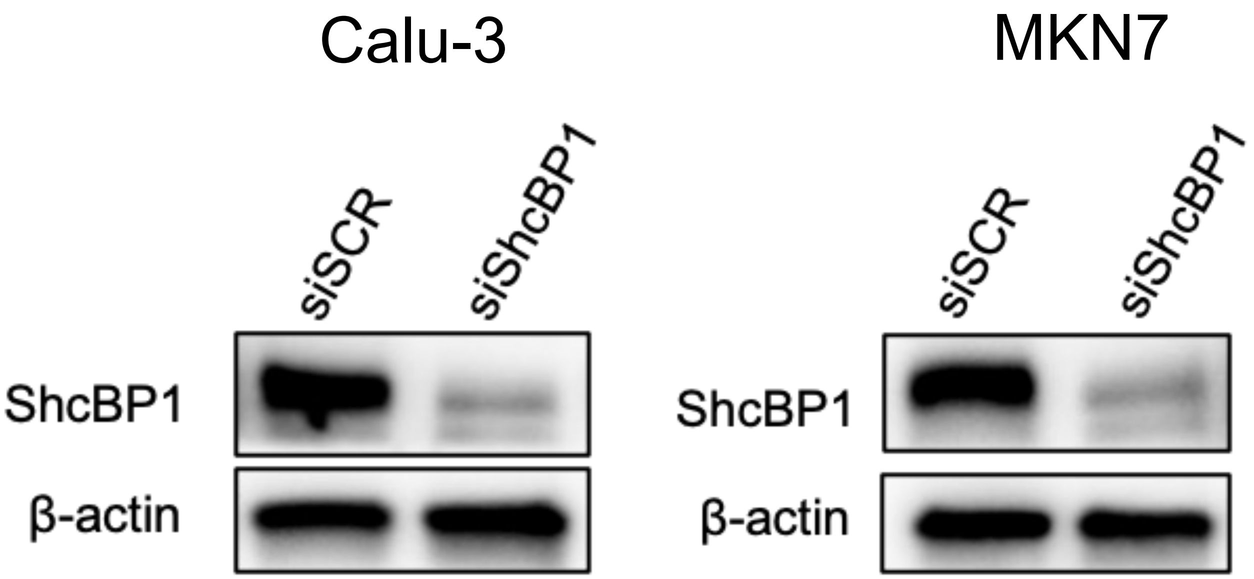

Supplementary Figure 14. Cell cycle analysis and knockdown effect of a ShcBP1-specific siRNA. (A) The cell cycle was analyzed via flow cytometry with propidium iodide (PI) following treatment with nonspecific control or ShcBP1-specific siRNAs with or without mobocertinib (0.1 μmol/L) in MKN7 cells. (B) Calu-3 and MKN7 cells were incubated with nonspecific or ShcBP1-specific siRNAs, lysed, and the indicated proteins were detected using western blotting.

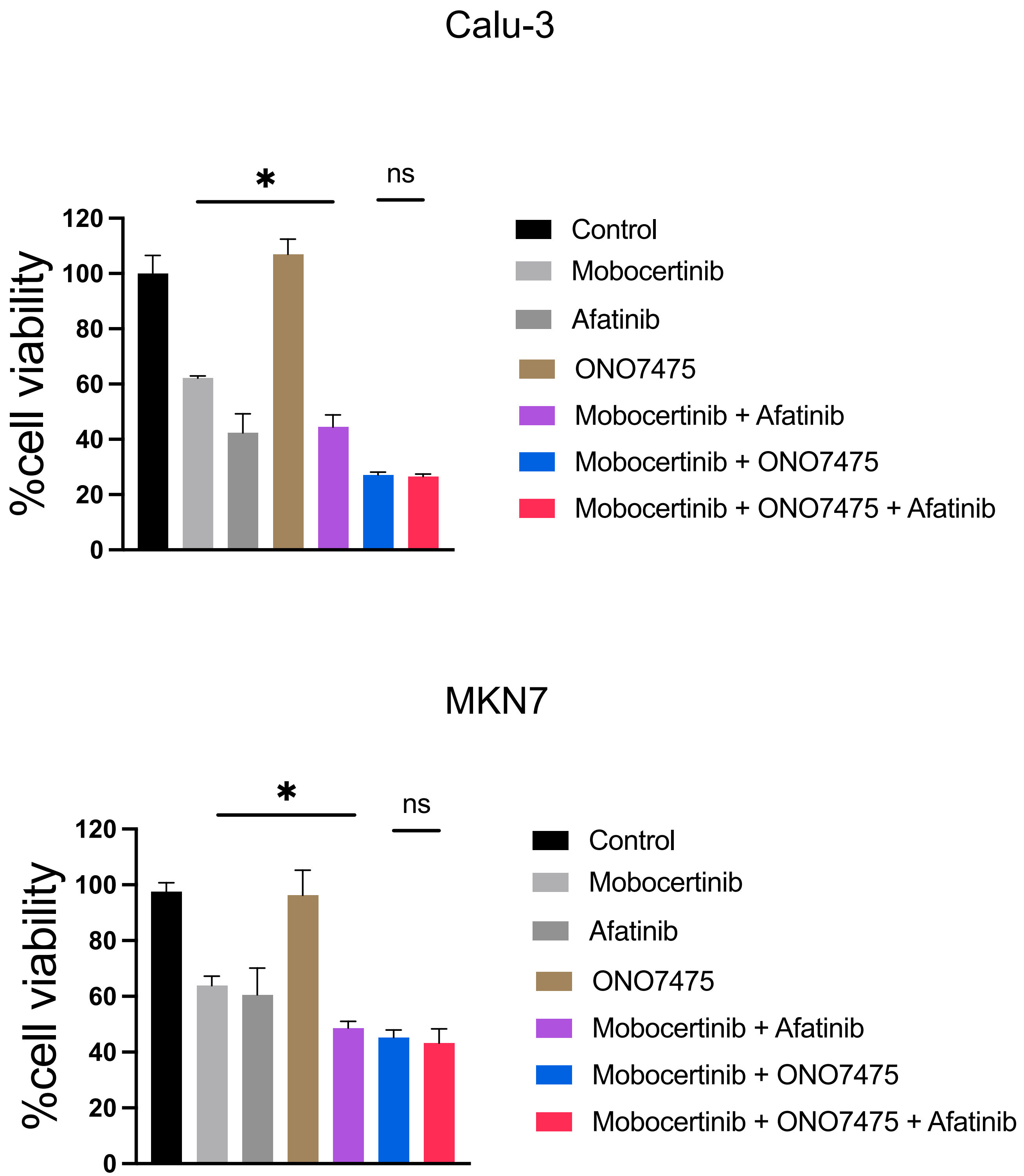

Supplementary Figure 15. Evaluation of the effect of afatinib on AXL-mediated resistance to HER2-TKI therapy.

Calu-3 and MKN7 cells were treated with mobocertinib (0.01 or 0.1 μmol/L), ONO7475 (0.3 μmol/L), and/or afatinib (0.01 or 0.1 μmol/L) for 72 h. Cell viability was assessed using an MTT assay.

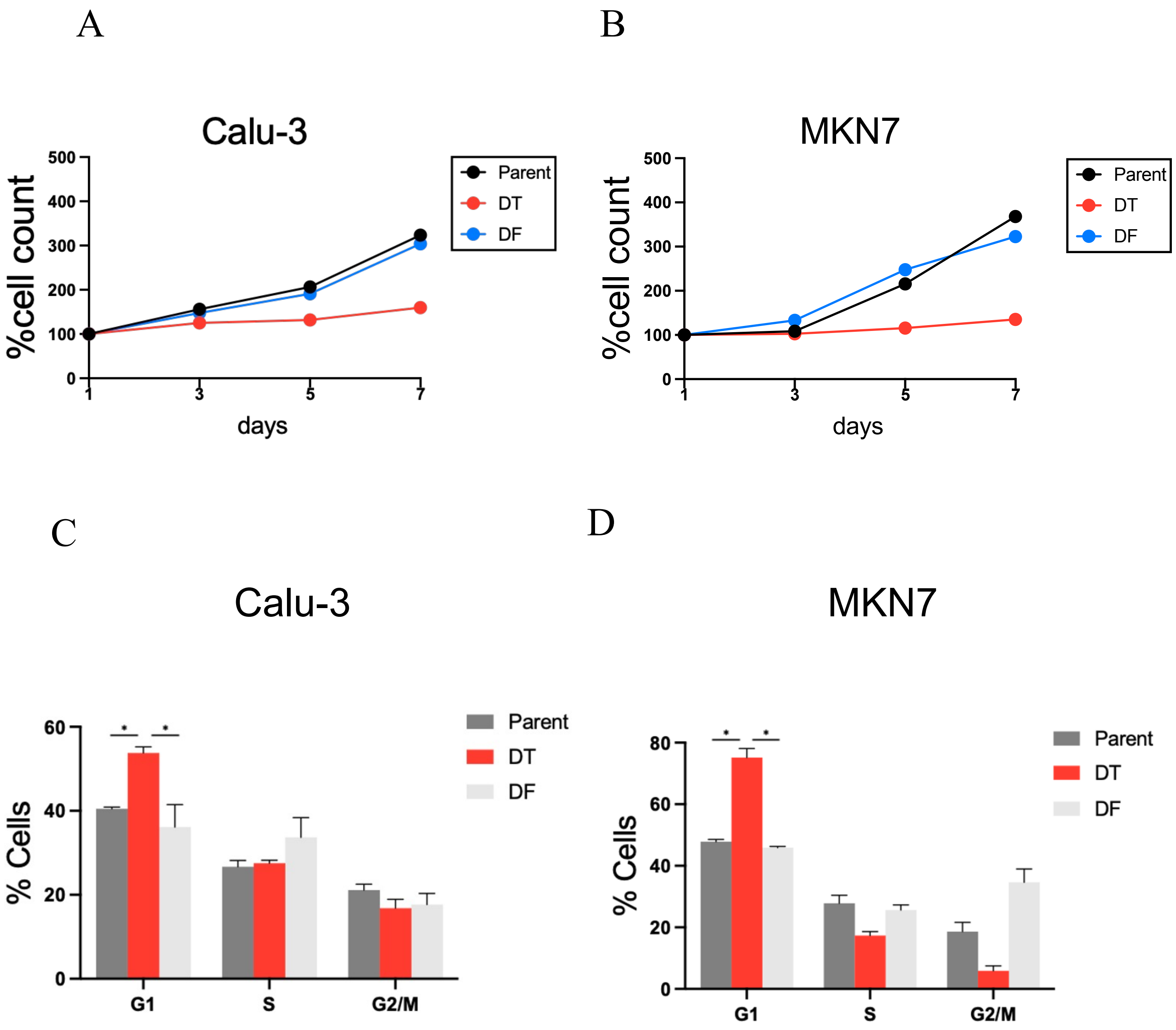

Supplementary Figure 16. Growth rate and cell cycle analysis of parent, drug-tolerant (DT) and drug-free (DF) cells.
Parent, DT, and DF cells of (A) Calu-3 and (B) MKN7 cell lines were treated with vehicle for the indicated duration. Cell counts were assessed using MTT assays. The cell cycle of parent, DT, and DF cells was analyzed using flow cytometry in (C) Calu-3 and (D) MKN7 cells. The proportion of the cell populations in the various cell-cycle phases are shown. *$P < 0.05$.

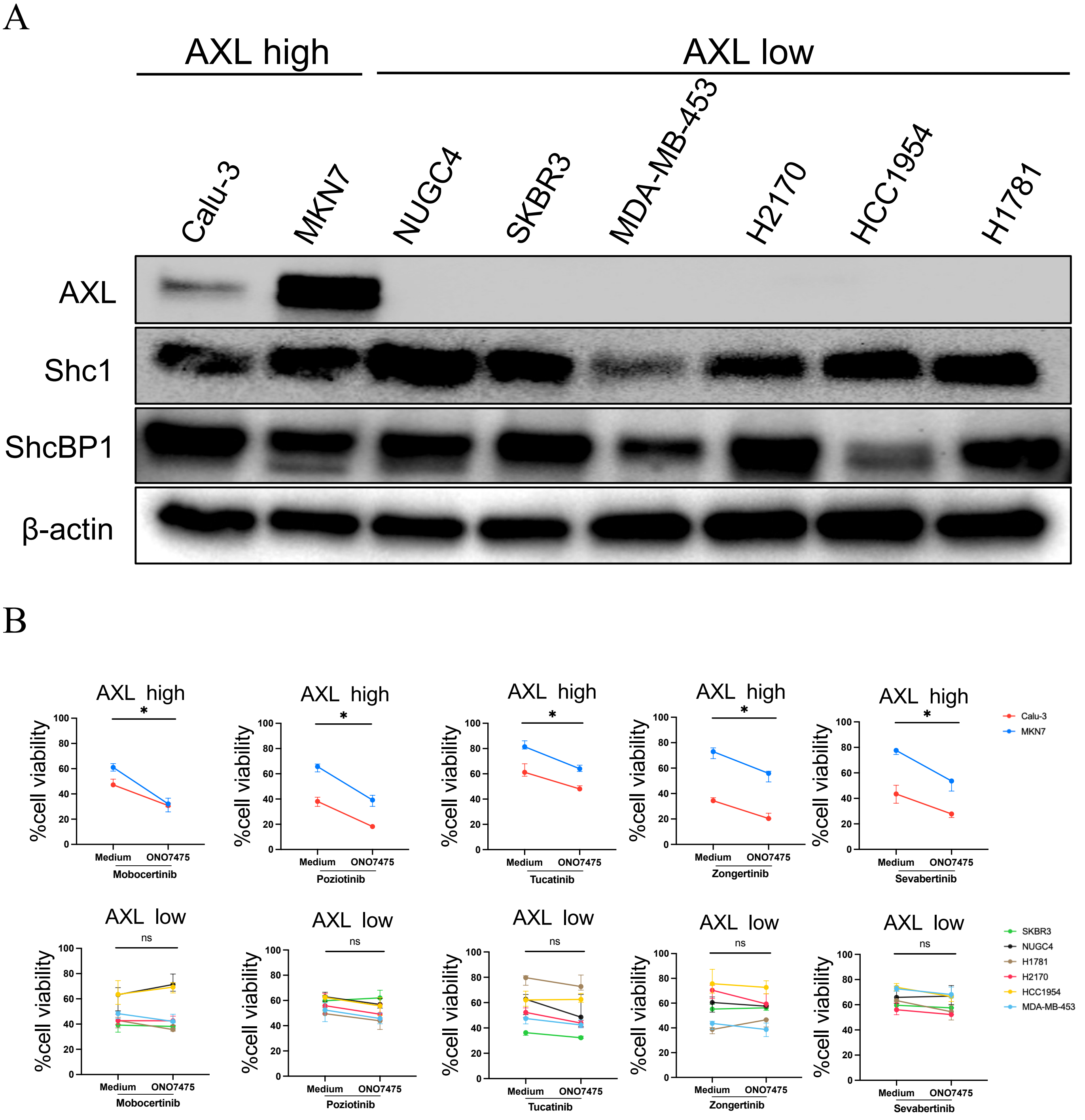

Supplementary Figure 17. Role of pre-treatment AXL expression on sensitivity to HER2-TKIs. (A) Western blotting analysis for the indicated proteins was performed on the HER2 aberrant cancer cell lines Calu-3, MKN7, NUGC4, SKBR3, MDA-MB-453, H2170, HCC1954, and H1781. (B) Quantification of the inhibition of cell viability in high-AXL-expressing and low-AXL-expressing HER2-aberrant cancer cells after treatment with mobocertinib (0.01 or 0.1 μmol/L), poziotinib (0.01 nmol/L or 0.1 nmol/L), tucatinib (0.1 or 1.0 μmol/L), zongertinib (0.1 or 1.0 μmol/L), or sevabertinib (0.1 or 1.0 μmol/L), alone or in combination with the AXL inhibitor ONO-7475 (0.3 μmol/L) for 72 h. Cell viability was assessed using MTT assays. Data are the mean ± SD of three independent experiments. *$P < 0.05$ (two-way ANOVA).

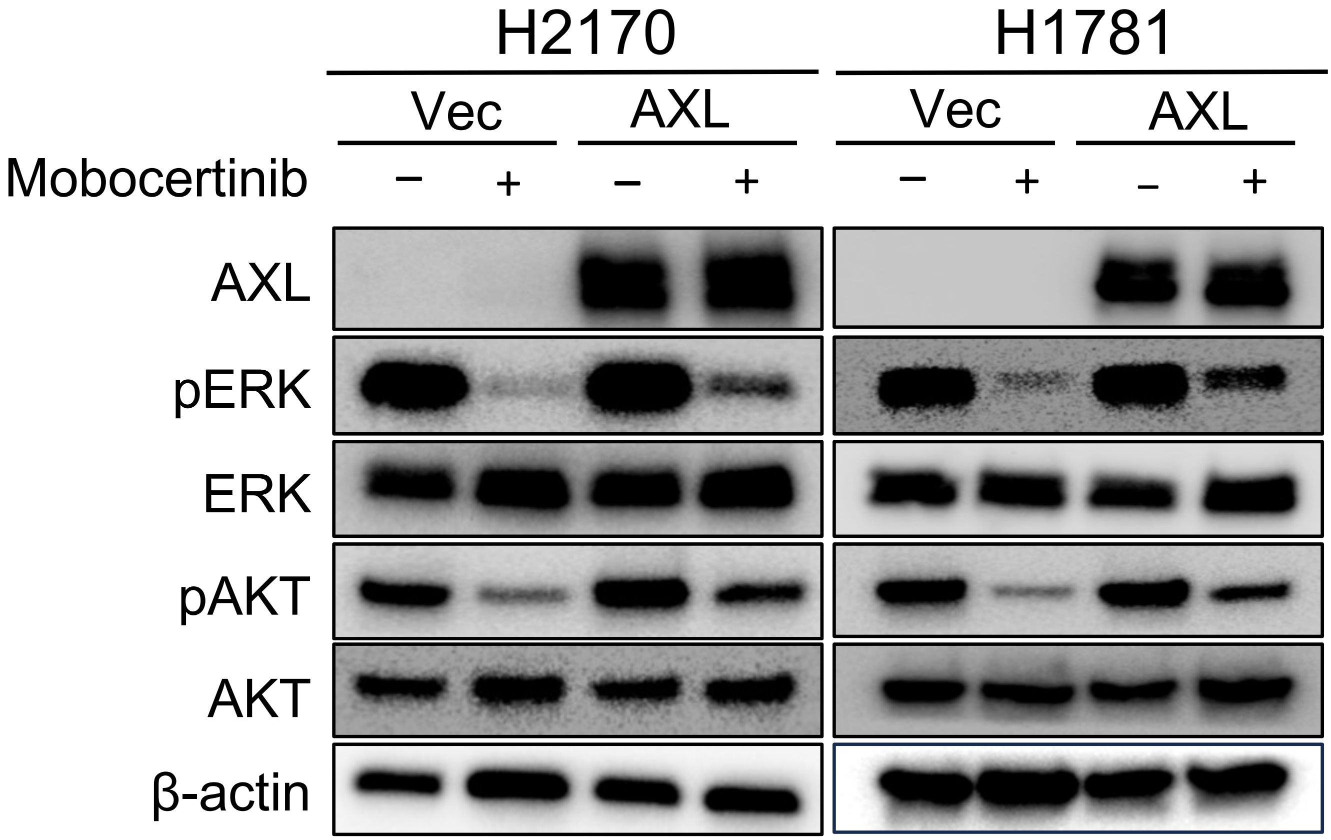

Supplementary Figure 18. AXL activation promoted mobocertinib resistance in HER2-aberrant tumor cells.

H2170 and H1781 cells were transfected with either an empty vector or human AXL. AXL protein expression was confirmed by western blotting. Cells were subsequently treated with 100 nM mobocertinib for 72 h.

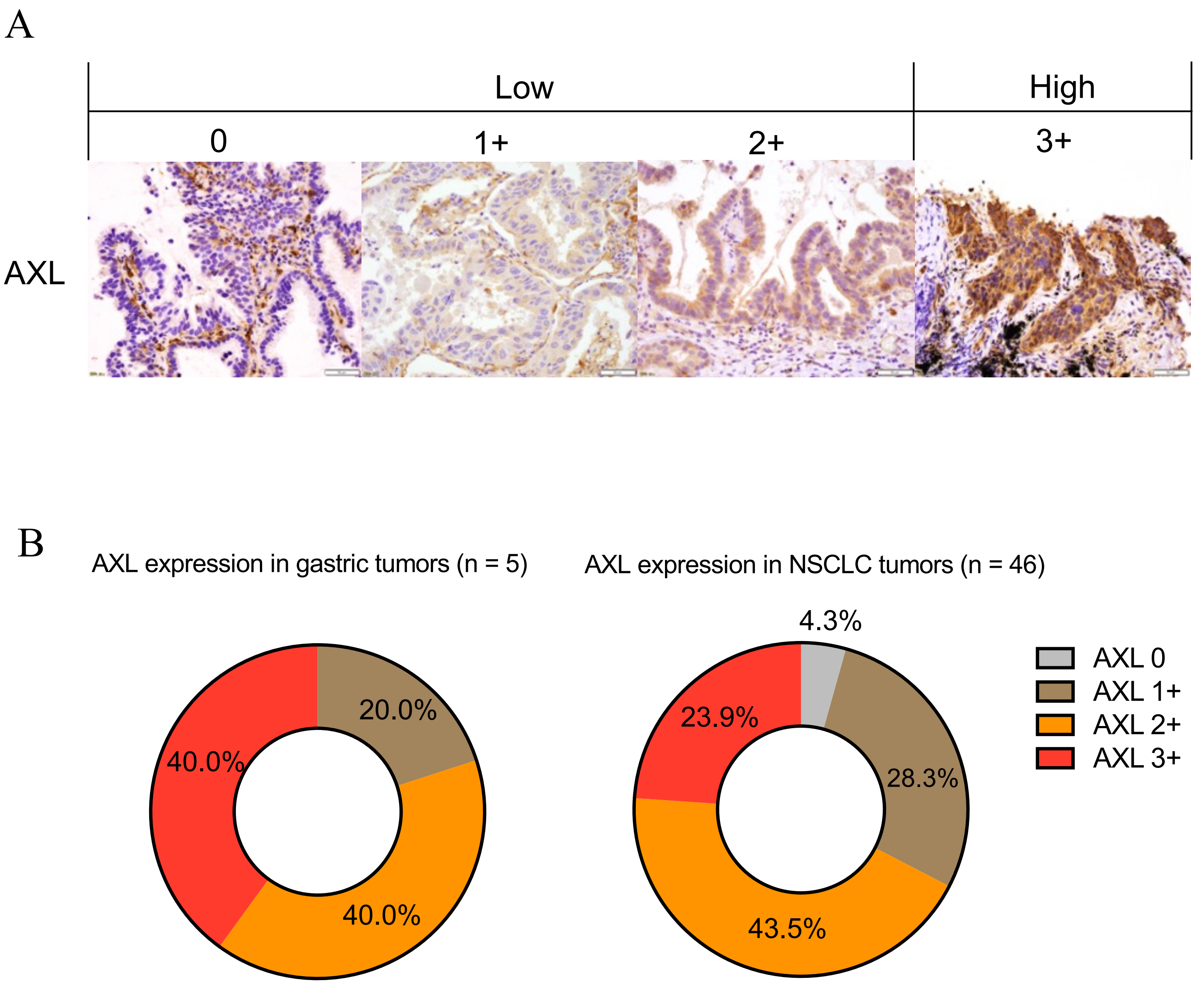

Supplementary Figure 19. Immunohistochemical analysis of AXL protein expression in HER2-aberrant NSCLC and gastric cancer specimens.

(A) AXL protein expression in immunohistochemical staining. Representative results of immunohistochemical staining for AXL. Bar, 50 μm. High AXL expression group, defined as tumors with AXL expression at 3+, and low AXL expression group, comprising tumors with AXL expression at 2+, 1+, or 0. AXL expression levels in tumor specimens from **(B)** five patients with gastric cancer, and forty-six HER2-aberrant patients with NSCLC.

A

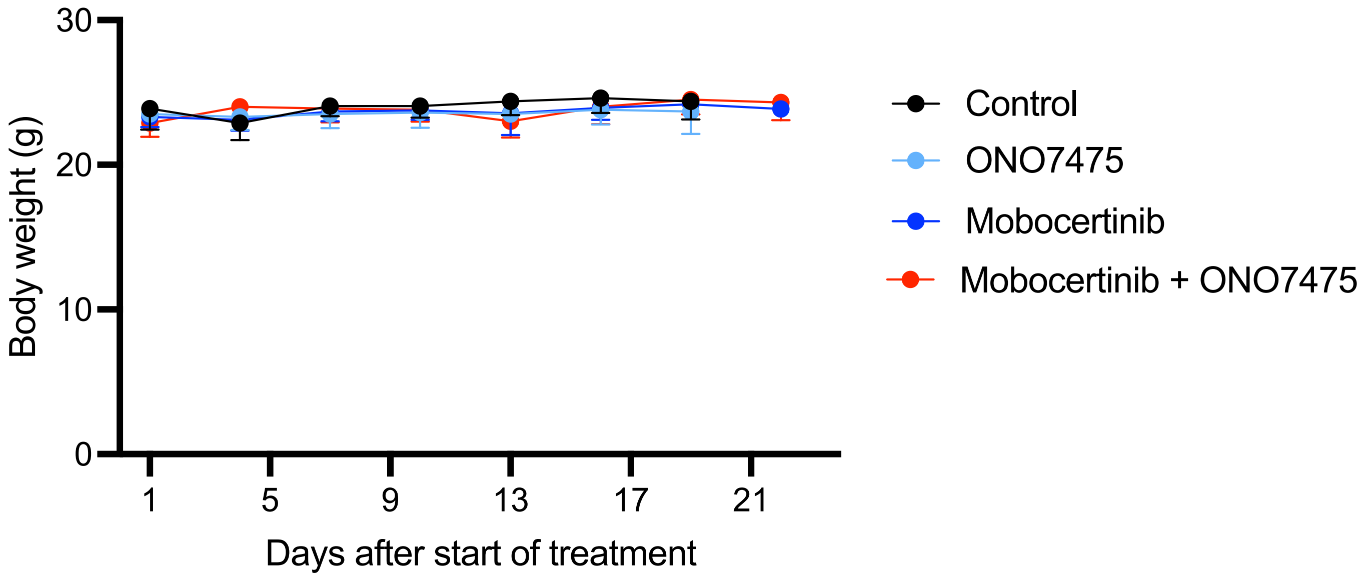

B

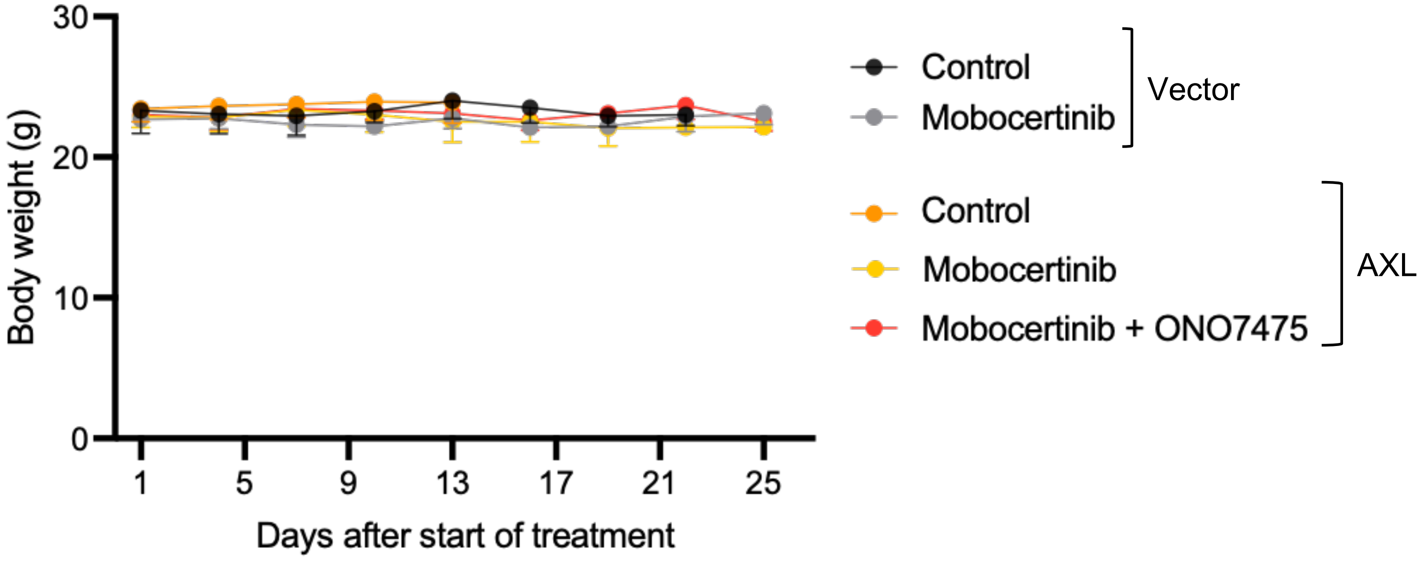

Supplementary Figure 20. Therapeutic tolerance in xenograft models of human HER2-aberrant tumor cells. Mouse weight was evaluated twice weekly.
(A) Calu-3 CDX tumors were treated with vehicle (control), mobocertinib (15 mg/kg), ONO7475 (10 mg/kg), or mobocertinib (15 mg/kg) plus ONO7475 (10 mg/kg) via daily oral gavage. (B) Transfected vector or AXL-overexpressing H2170 CDX tumors were treated with vehicle (control) or mobocertinib (10 mg/kg) via daily oral gavage.

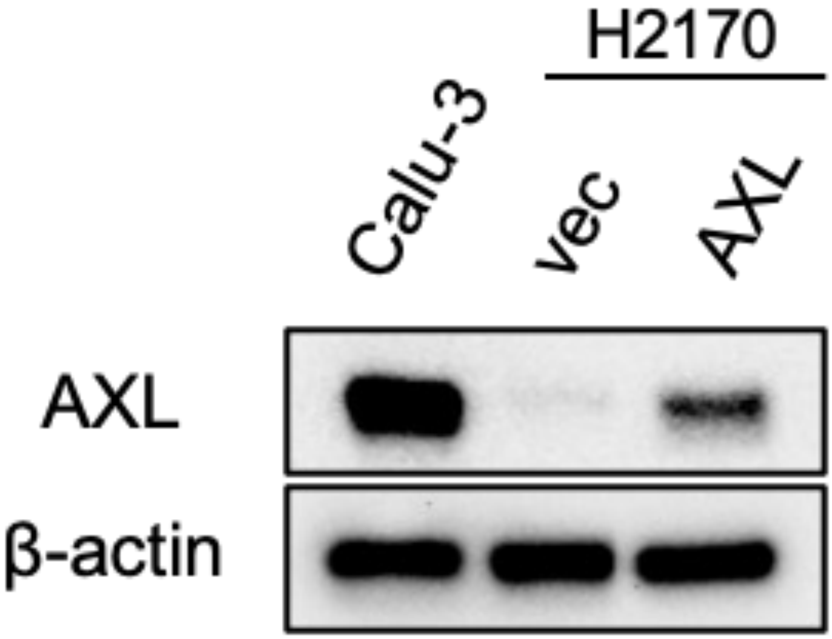

Supplementary Figure 21. H2170 cells transfected with human AXL.
The AXL protein was detected in the harvested tumors by western blotting.

Supplementary Figure 22. Uncropped blots for main figures. Blots shown in the main article are depicted by boxed regions in each of associated uncropped scans.

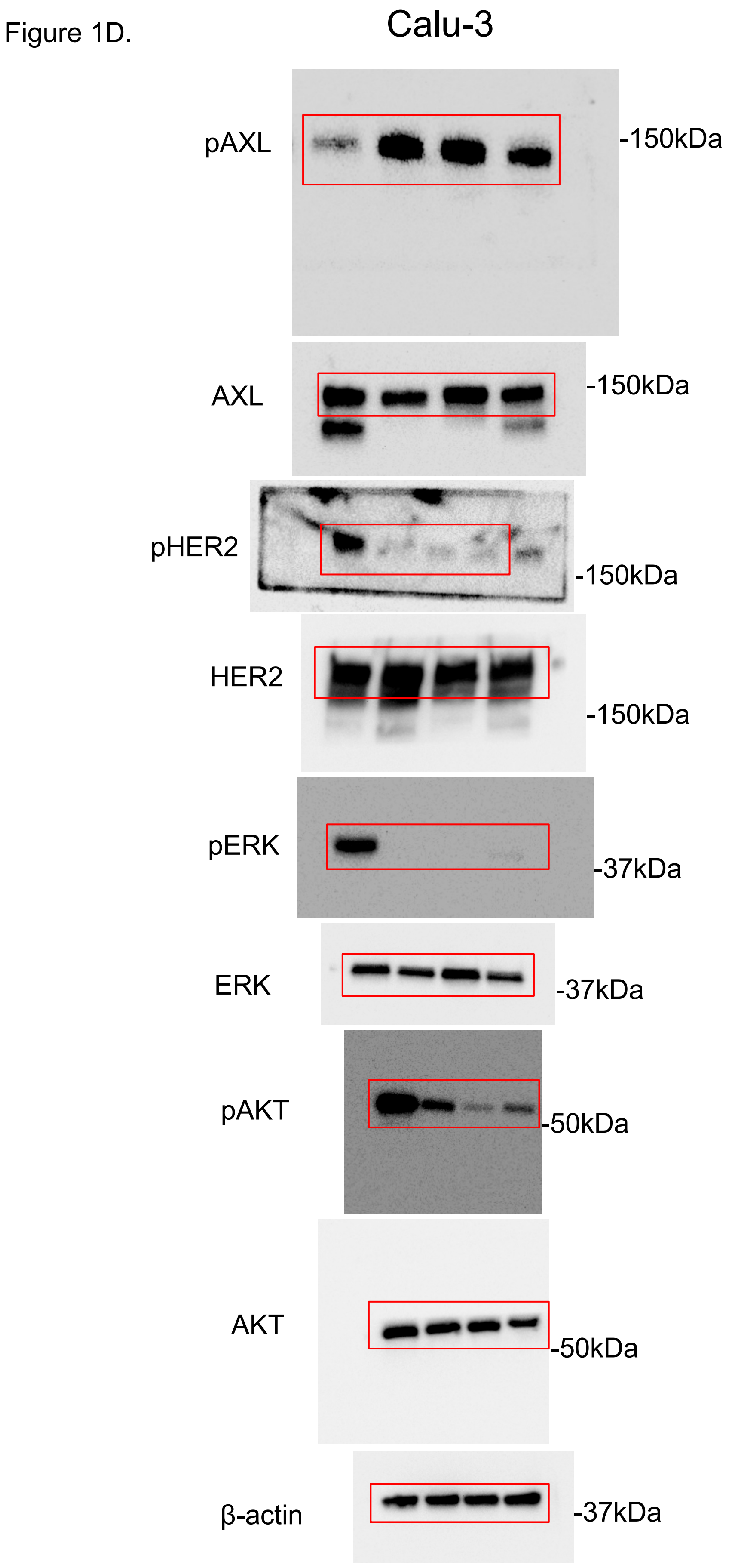

Figure 1D.

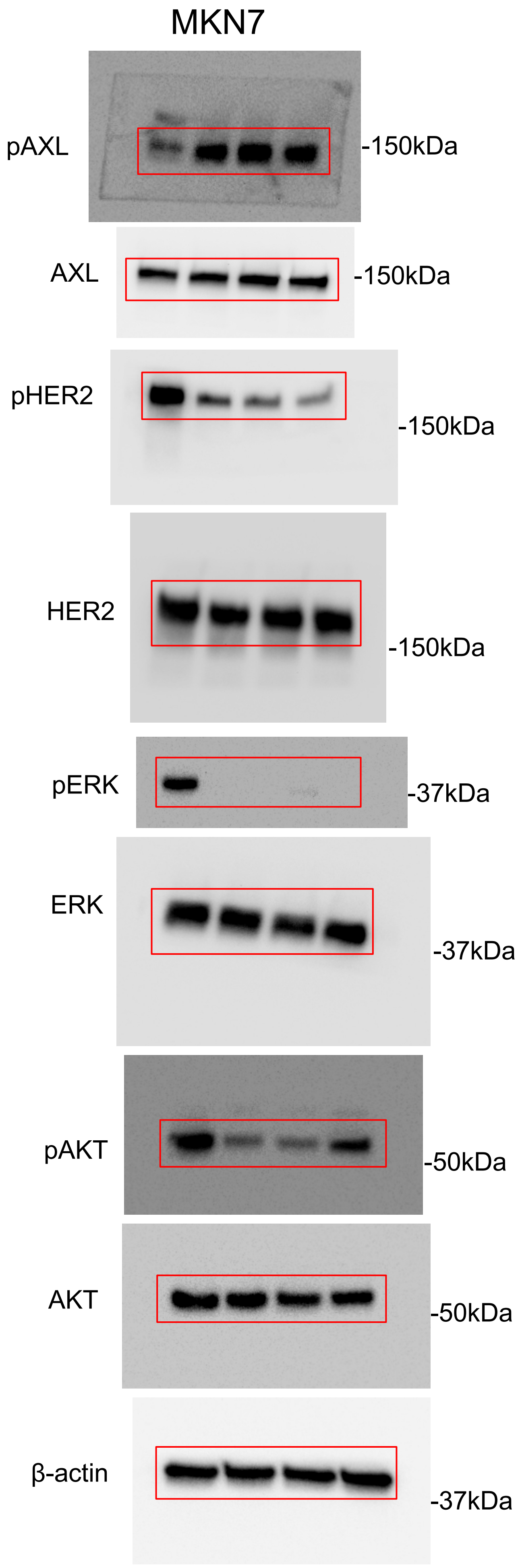
MKN7
pAXL
-150kDa
AXL
-150kDa
pHER2
-150kDa
HER2
-150kDa
pERK
-37kDa
ERK
-37kDa
pAKT
-50kDa
AKT
-50kDa
β-actin
-37kDa

Figure 1F.

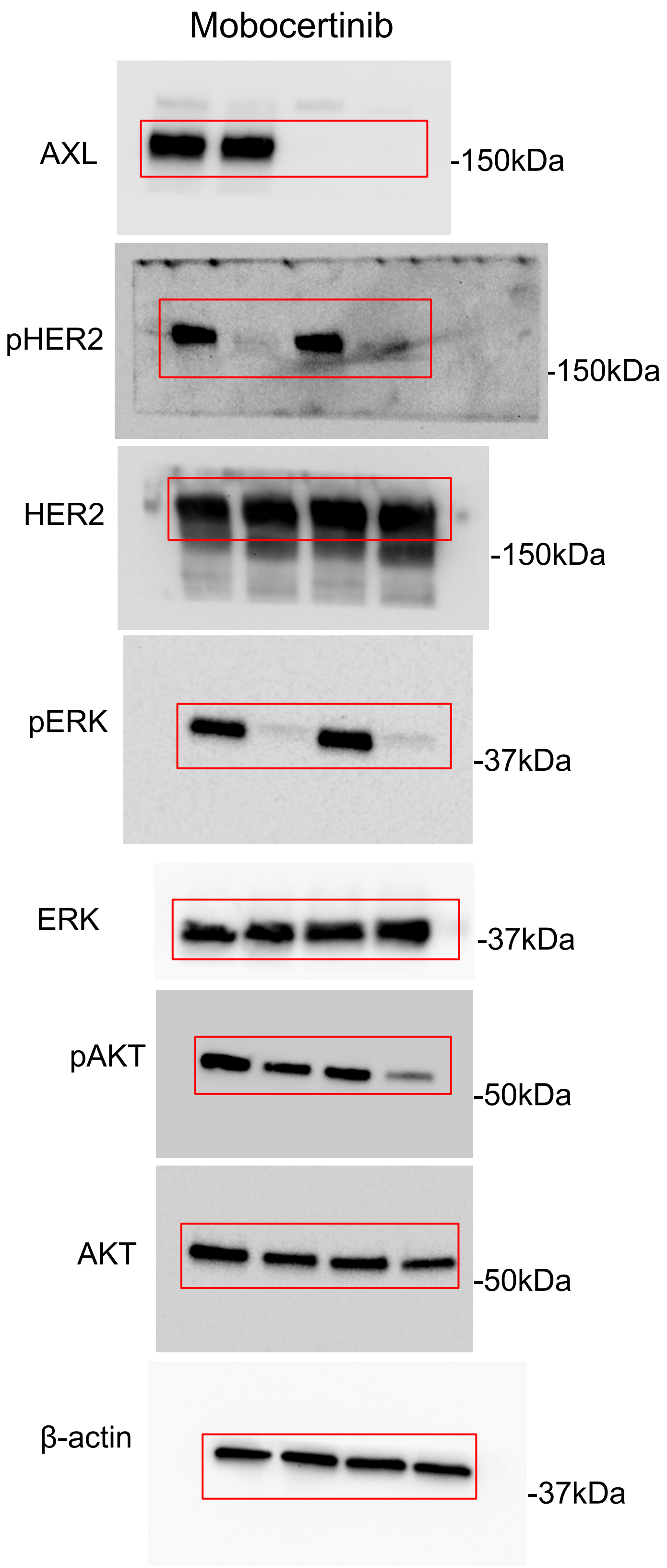

Mobocertinib
AXL
-150kDa
pHER2
-150kDa
HER2
-150kDa
pERK
-37kDa
ERK
-37kDa
pAKT
-50kDa
AKT
-50kDa
β-actin
-37kDa

Figure 1F.

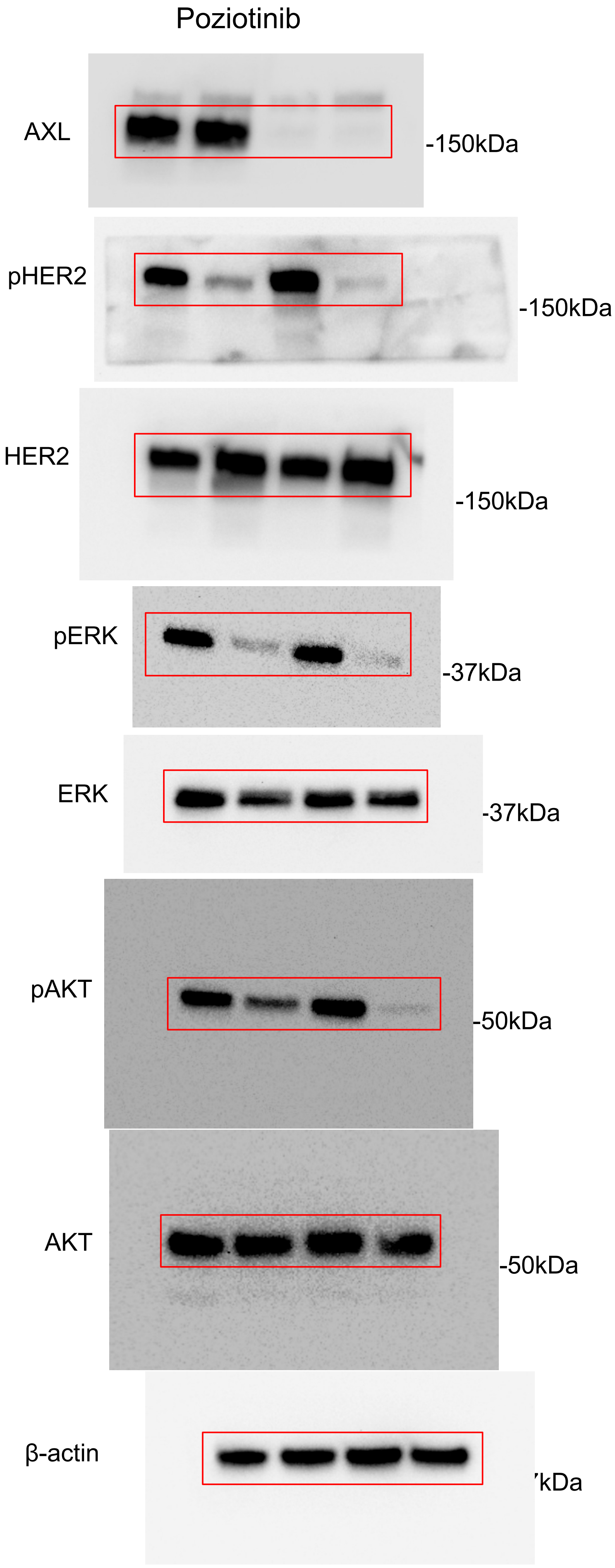
Poziotinib
AXL
-150kDa
pHER2
-150kDa
HER2
-150kDa
pERK
-37kDa
ERK
-37kDa
pAKT
-50kDa
AKT
-50kDa
β-actin

Figure 1F.

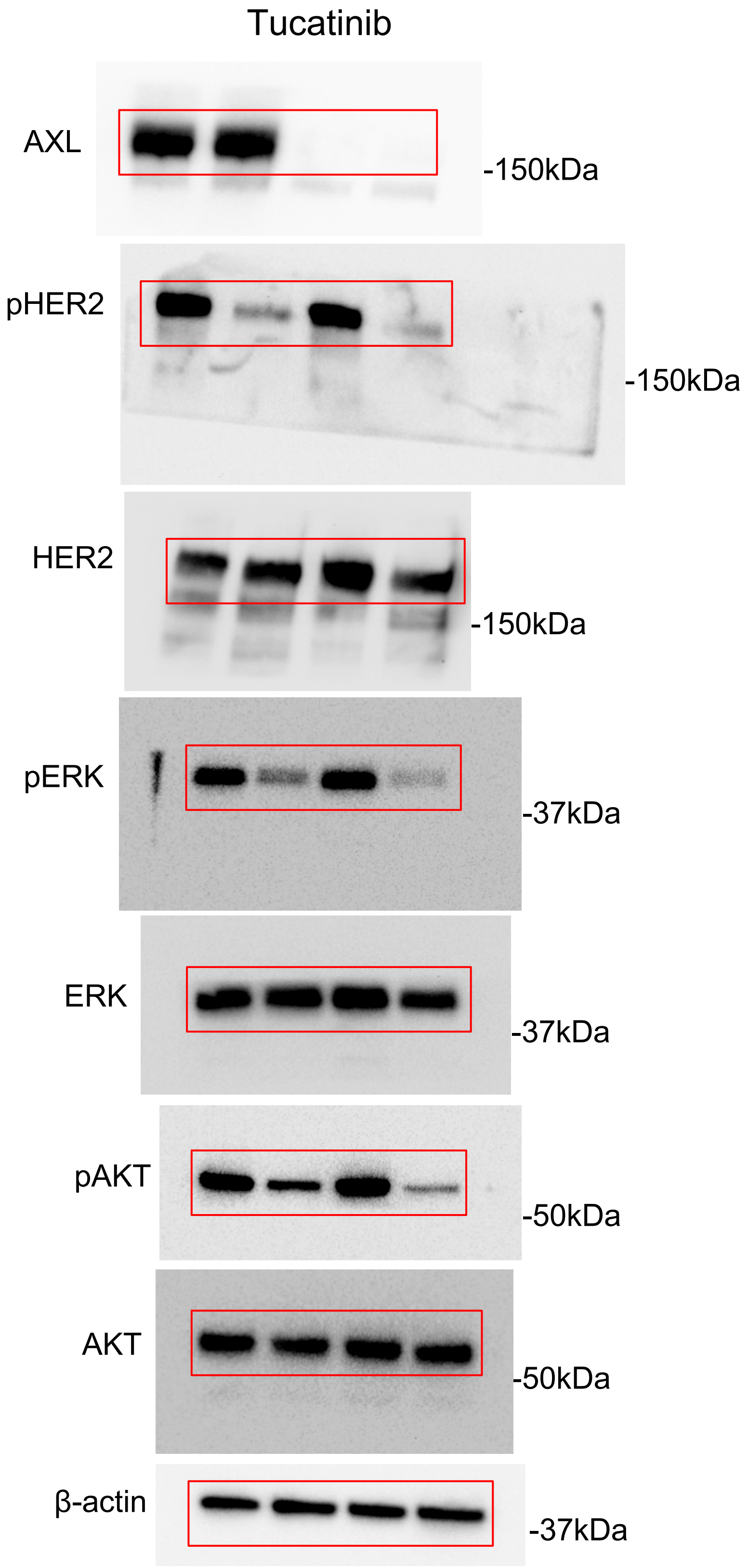

Tucatinib
AXL
-150kDa
pHER2
-150kDa
HER2
-150kDa
pERK
-37kDa
ERK
-37kDa
pAKT
-50kDa
AKT
-50kDa
β-actin
-37kDa

Figure 1G.

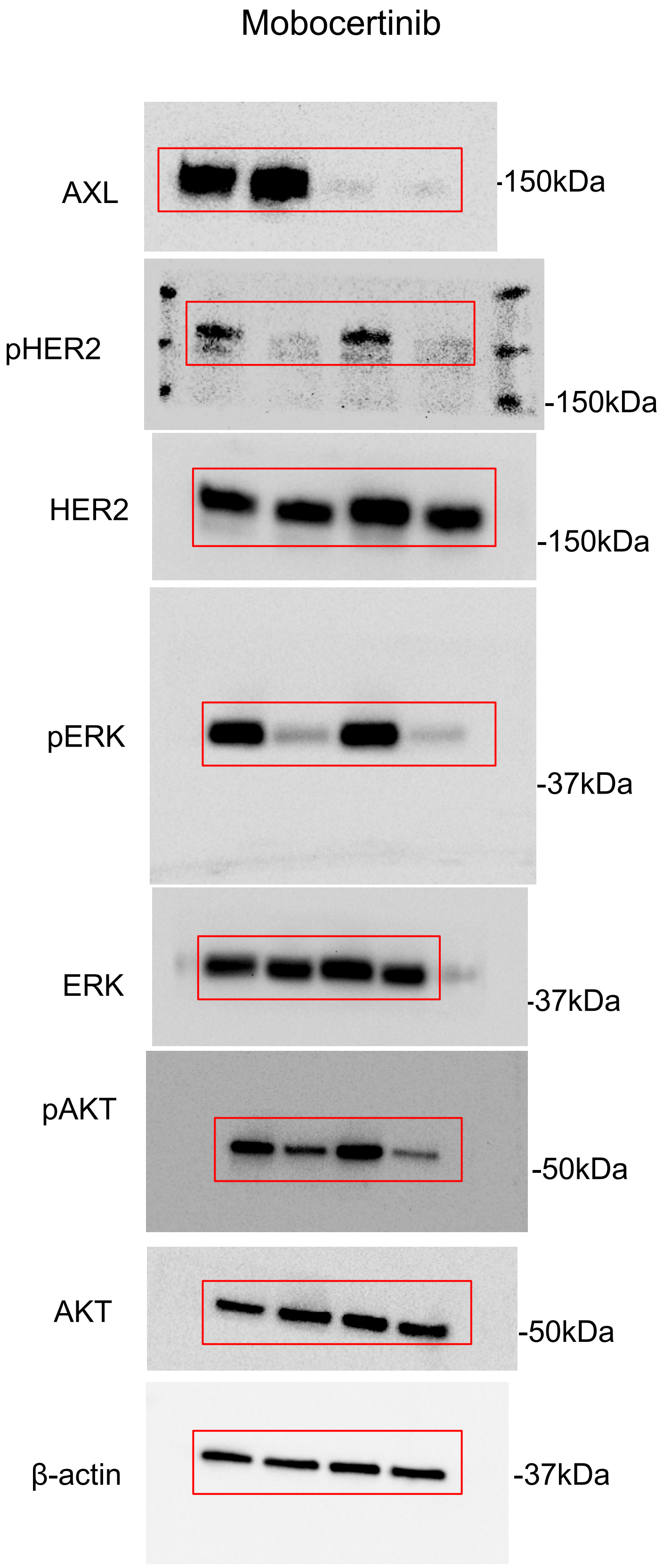
Mobocertinib
AXL
-150kDa
pHER2
-150kDa
HER2
-150kDa
pERK
-37kDa
ERK
-37kDa
pAKT
-50kDa
AKT
-50kDa
β-actin
-37kDa

Figure 1G.

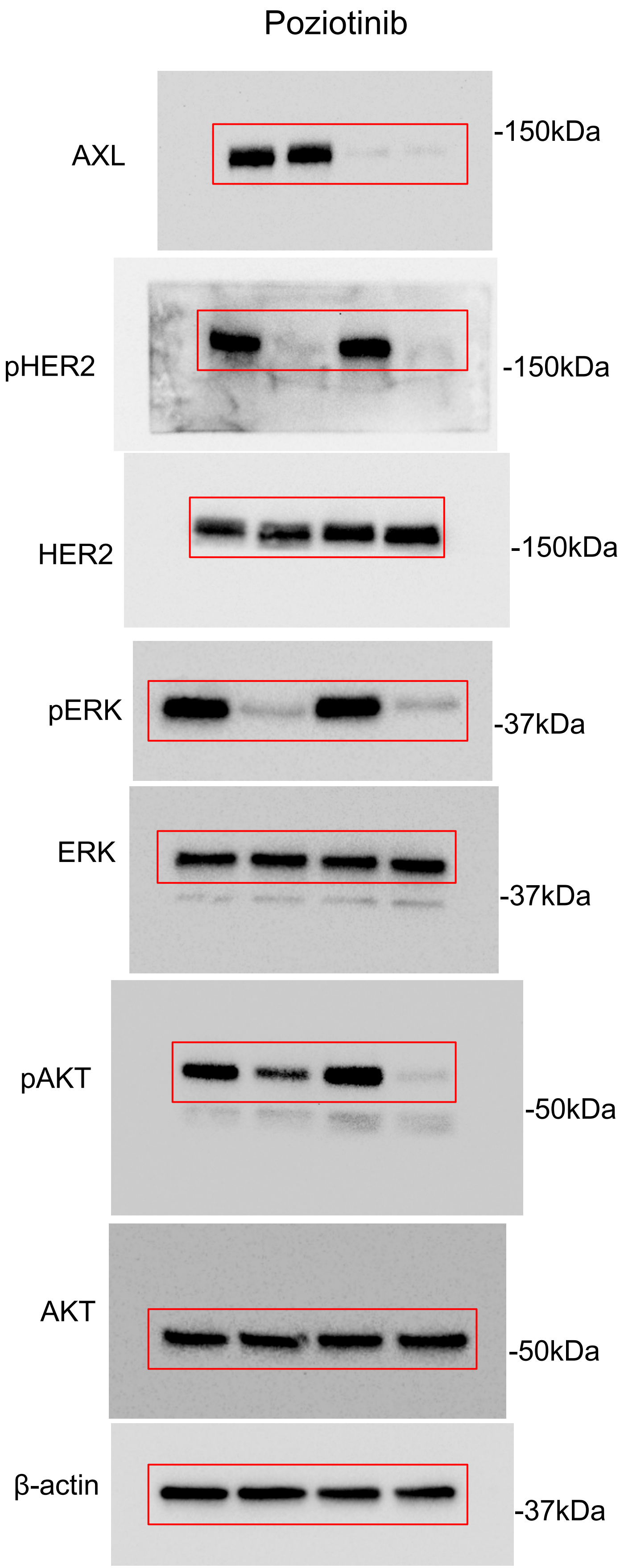
Poziotinib
AXL
-150kDa
pHER2
-150kDa
HER2
-150kDa
pERK
-37kDa
ERK
-37kDa
pAKT
-50kDa
AKT
-50kDa
β-actin
-37kDa

Figure 1G.

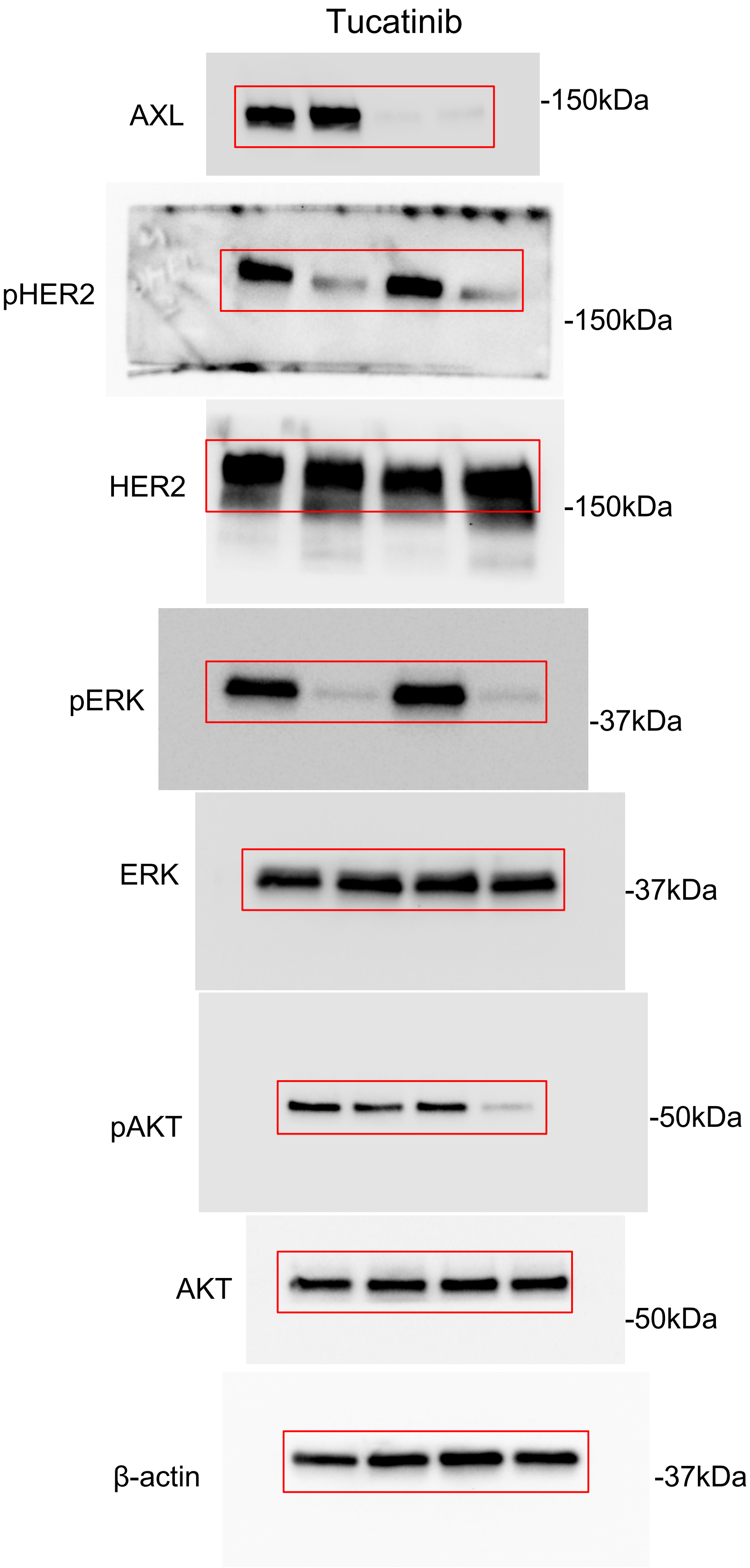
Tucatinib
AXL
-150kDa
pHER2
-150kDa
HER2
-150kDa
pERK
-37kDa
ERK
-37kDa
pAKT
-50kDa
AKT
-50kDa
β-actin
-37kDa

Figure 2C.

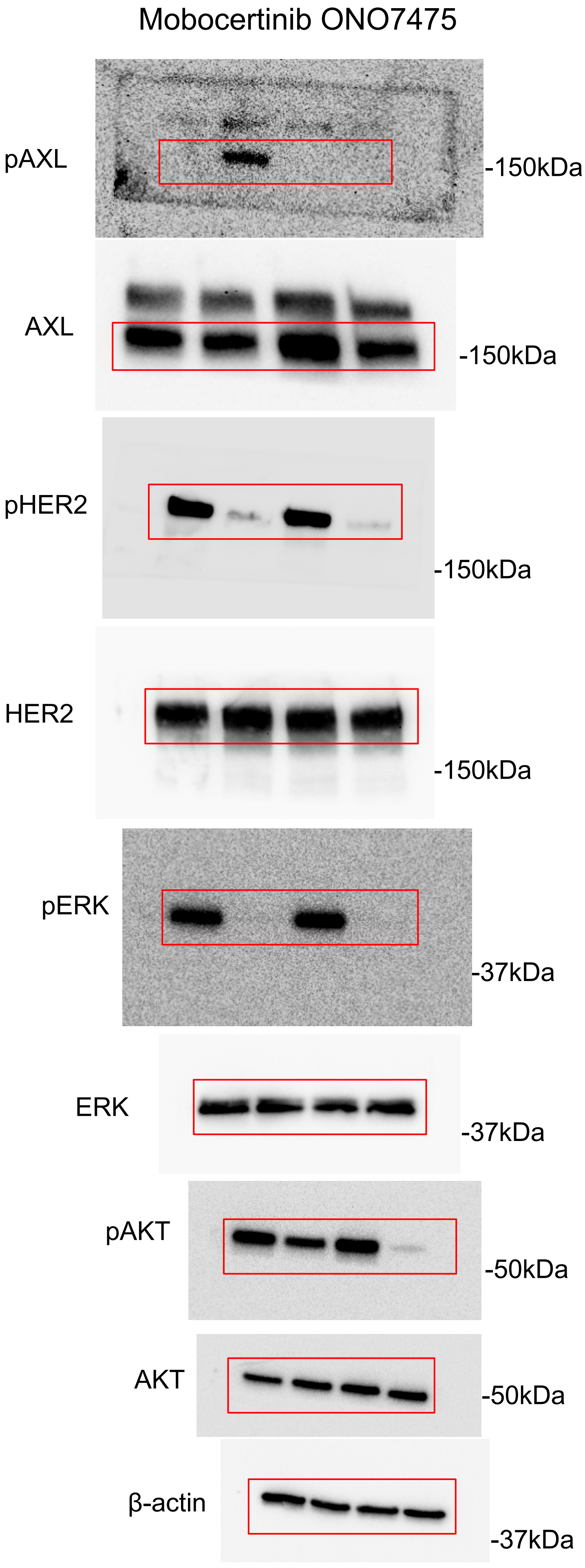
Mobocertinib ONO7475
pAXL
-150kDa
AXL
-150kDa
pHER2
-150kDa
HER2
-150kDa
pERK
-37kDa
ERK
-37kDa
pAKT
-50kDa
AKT
-50kDa
β-actin
-37kDa

Figure 2C.

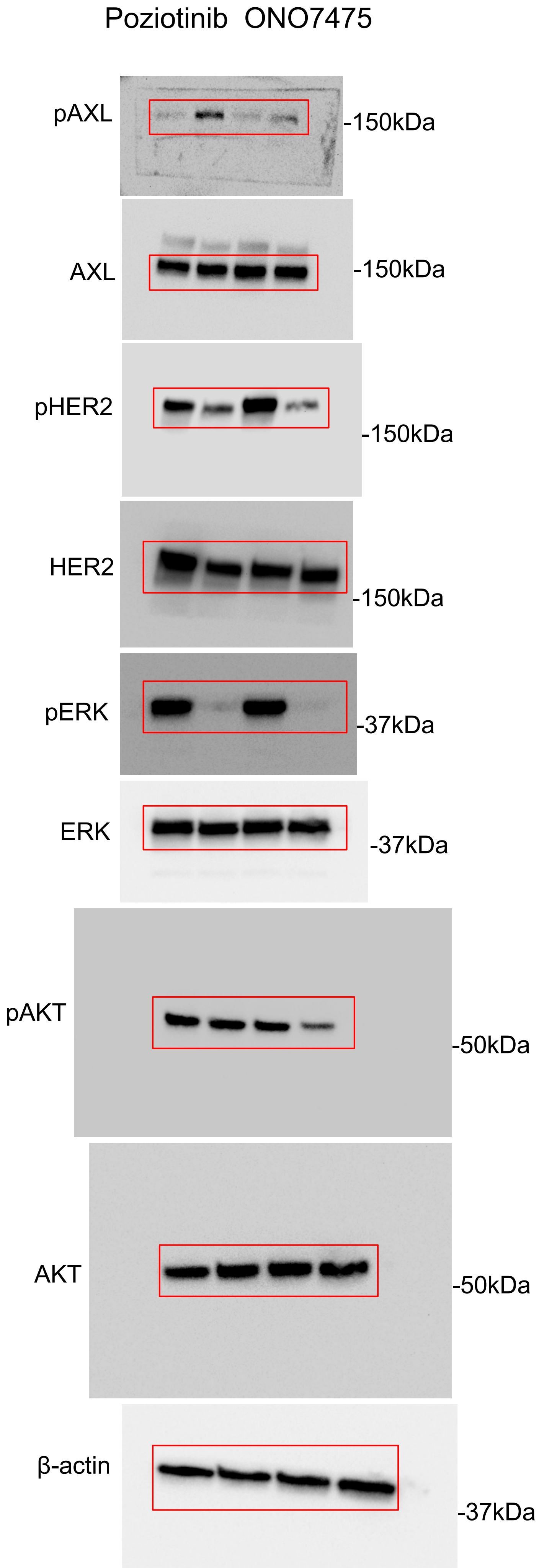
Poziotinib ONO7475
pAXL
-150kDa
AXL
-150kDa
pHER2
-150kDa
HER2
-150kDa
pERK
-37kDa
ERK
-37kDa
pAKT
-50kDa
AKT
-50kDa
β-actin
-37kDa

Figure 2C.

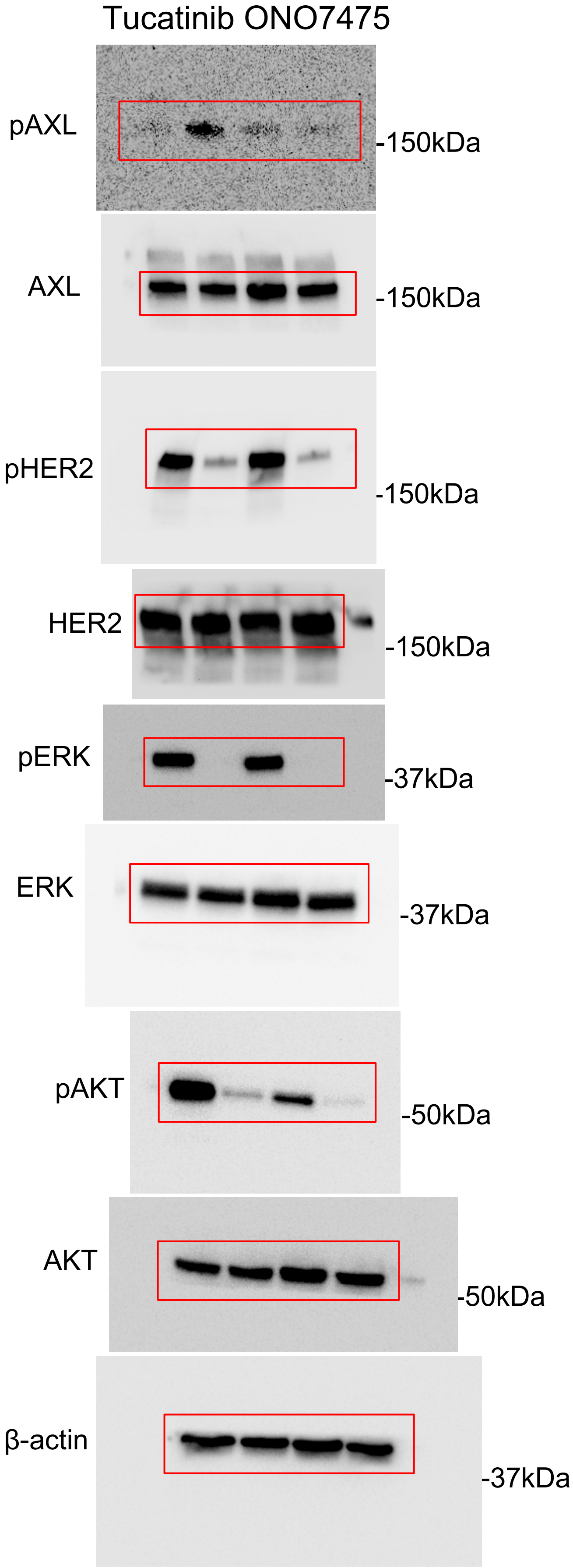
Tucatinib ONO7475
pAXL
-150kDa
AXL
-150kDa
pHER2
-150kDa
HER2
-150kDa
pERK
-37kDa
ERK
-37kDa
pAKT
-50kDa
AKT
-50kDa
β-actin
-37kDa

Figure 2D.

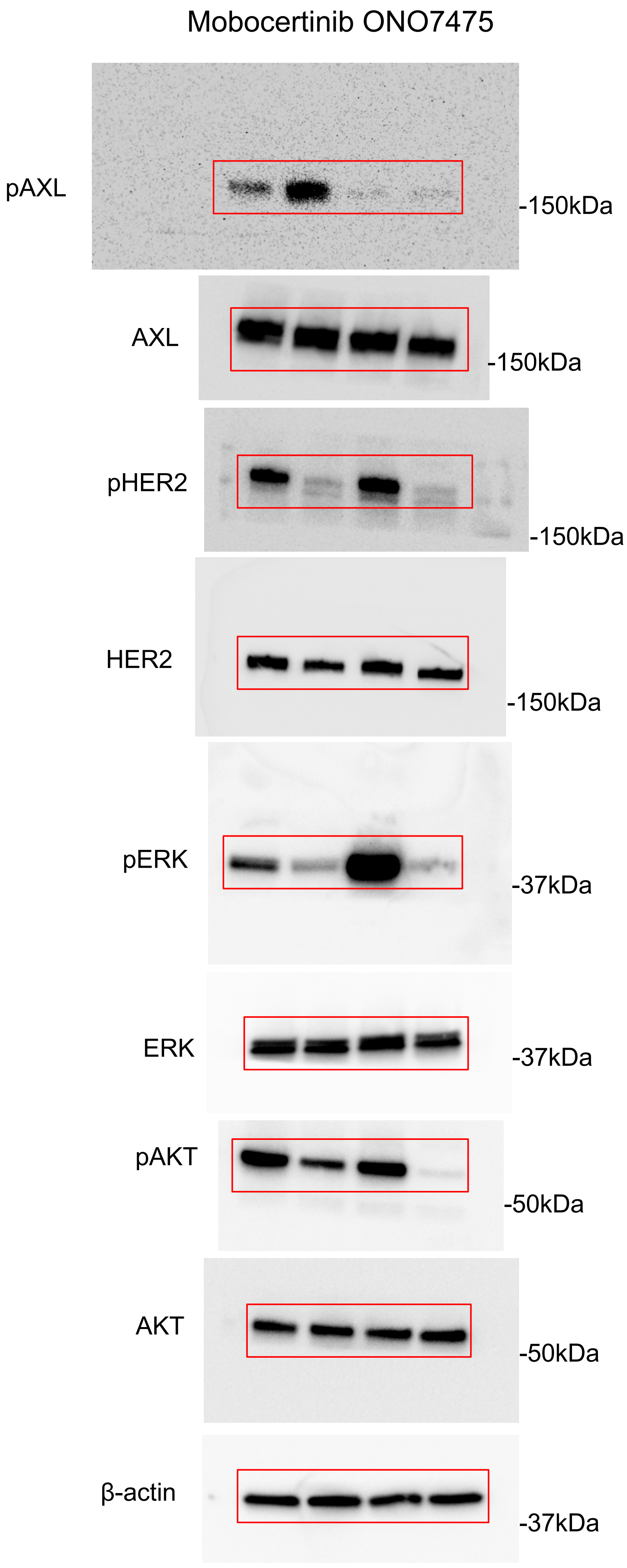
Mobocertinib ONO7475
pAXL
-150kDa
AXL
-150kDa
pHER2
-150kDa
HER2
-150kDa
pERK
-37kDa
ERK
-37kDa
pAKT
-50kDa
AKT
-50kDa
β-actin
-37kDa

Figure 2D.

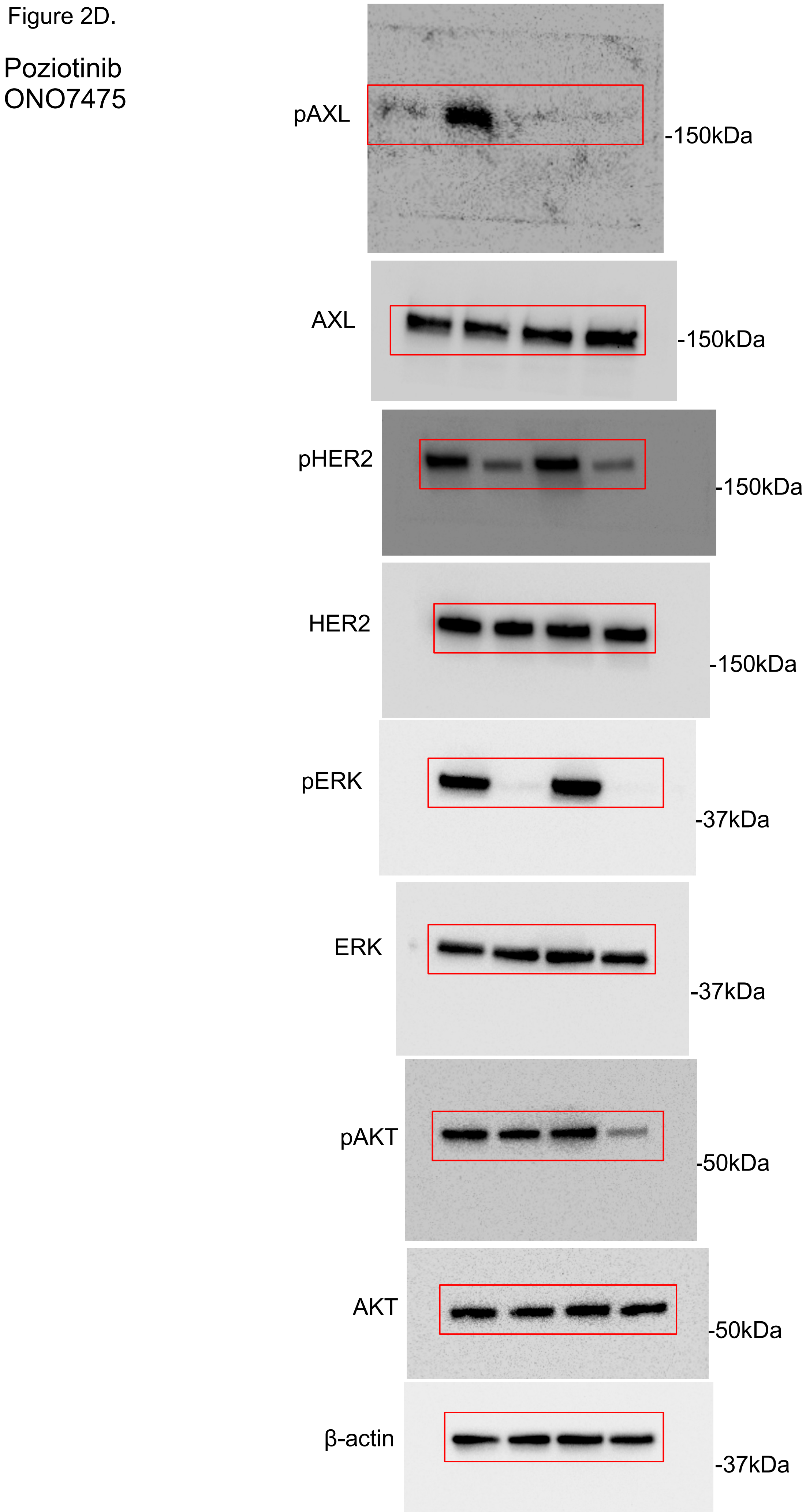
Poziotinib
ONO7475
pAXL
-150kDa
AXL
-150kDa
pHER2
-150kDa
HER2
-150kDa
pERK
-37kDa
ERK
-37kDa
pAKT
-50kDa
AKT
-50kDa
β-actin
-37kDa

Figure 2D.

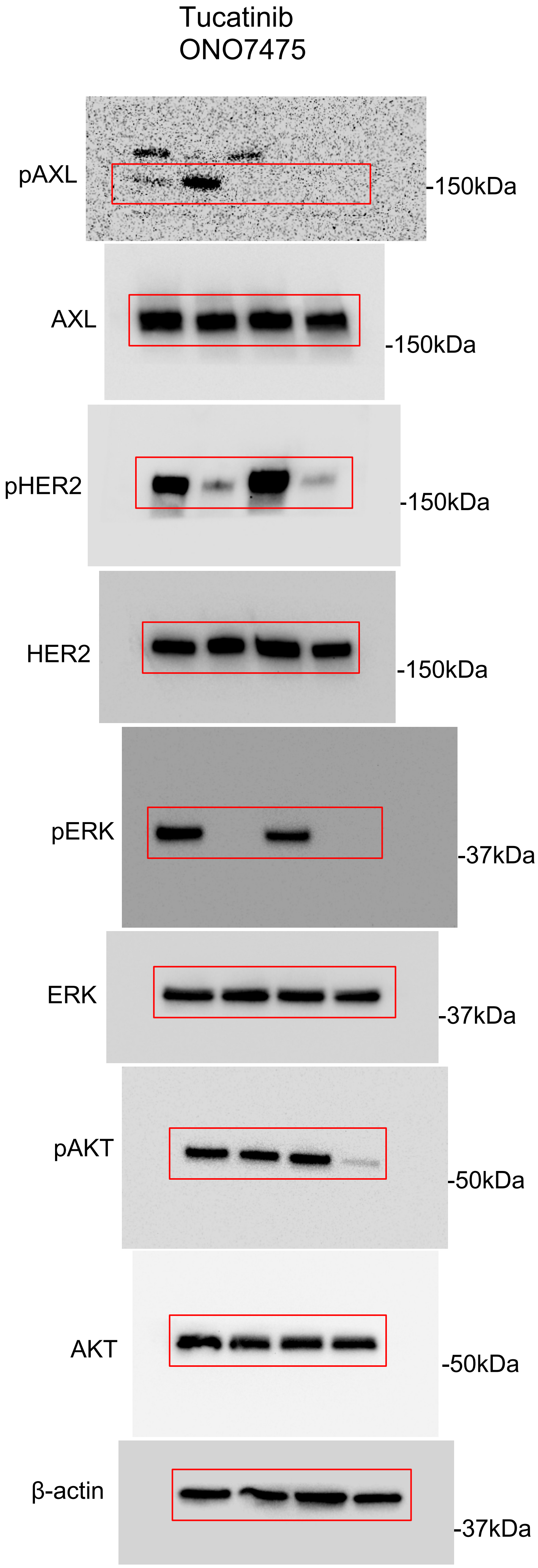
Tucatinib
ONO7475
pAXL
-150kDa
AXL
-150kDa
pHER2
-150kDa
HER2
-150kDa
pERK
-37kDa
ERK
-37kDa
pAKT
-50kDa
AKT
-50kDa
β-actin
-37kDa

Figure 3C.

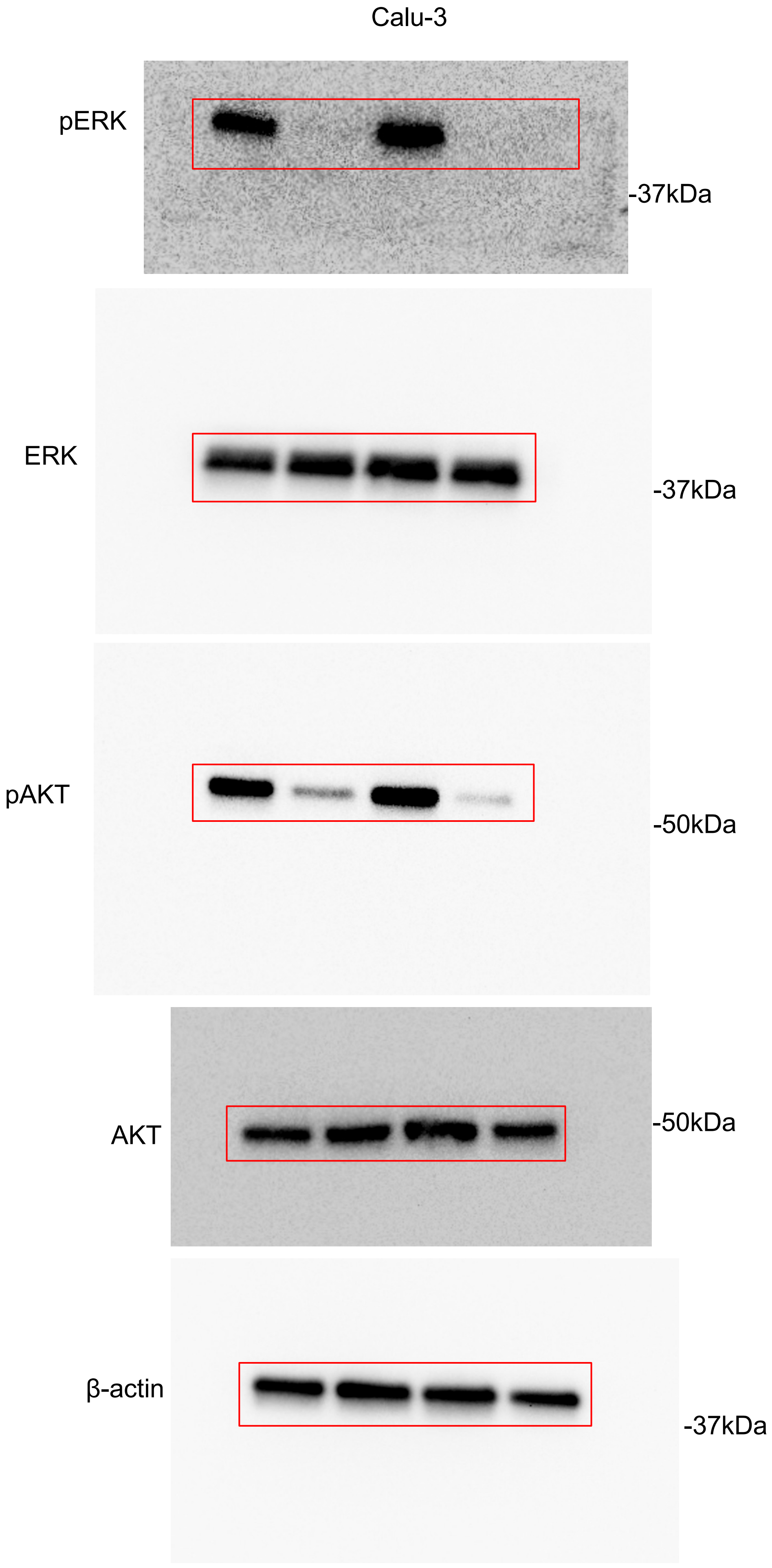
Calu-3
pERK
-37kDa
ERK
-37kDa
pAKT
-50kDa
AKT
-50kDa
β-actin
-37kDa

Figure 3C.

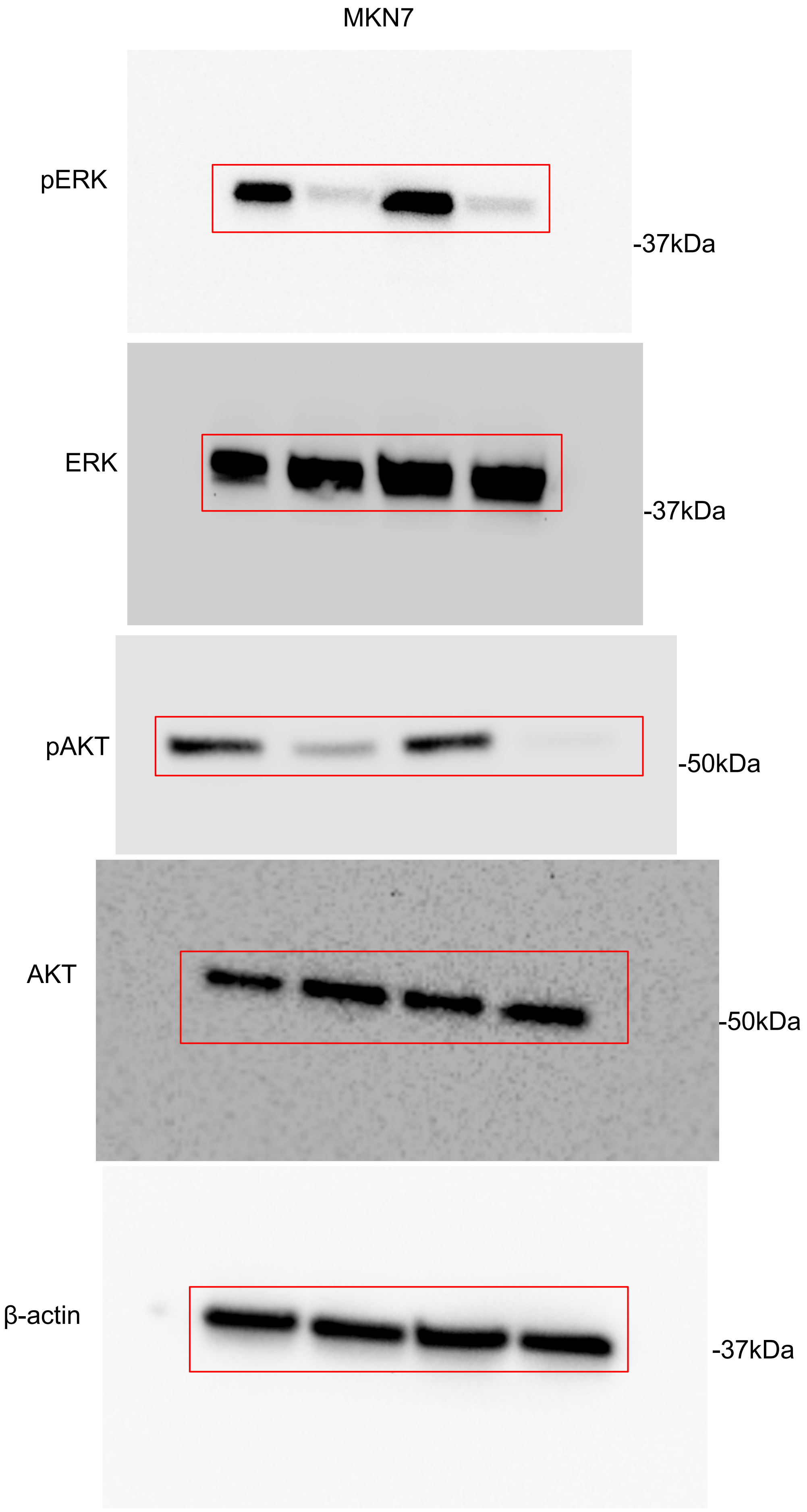

Figure 3E.

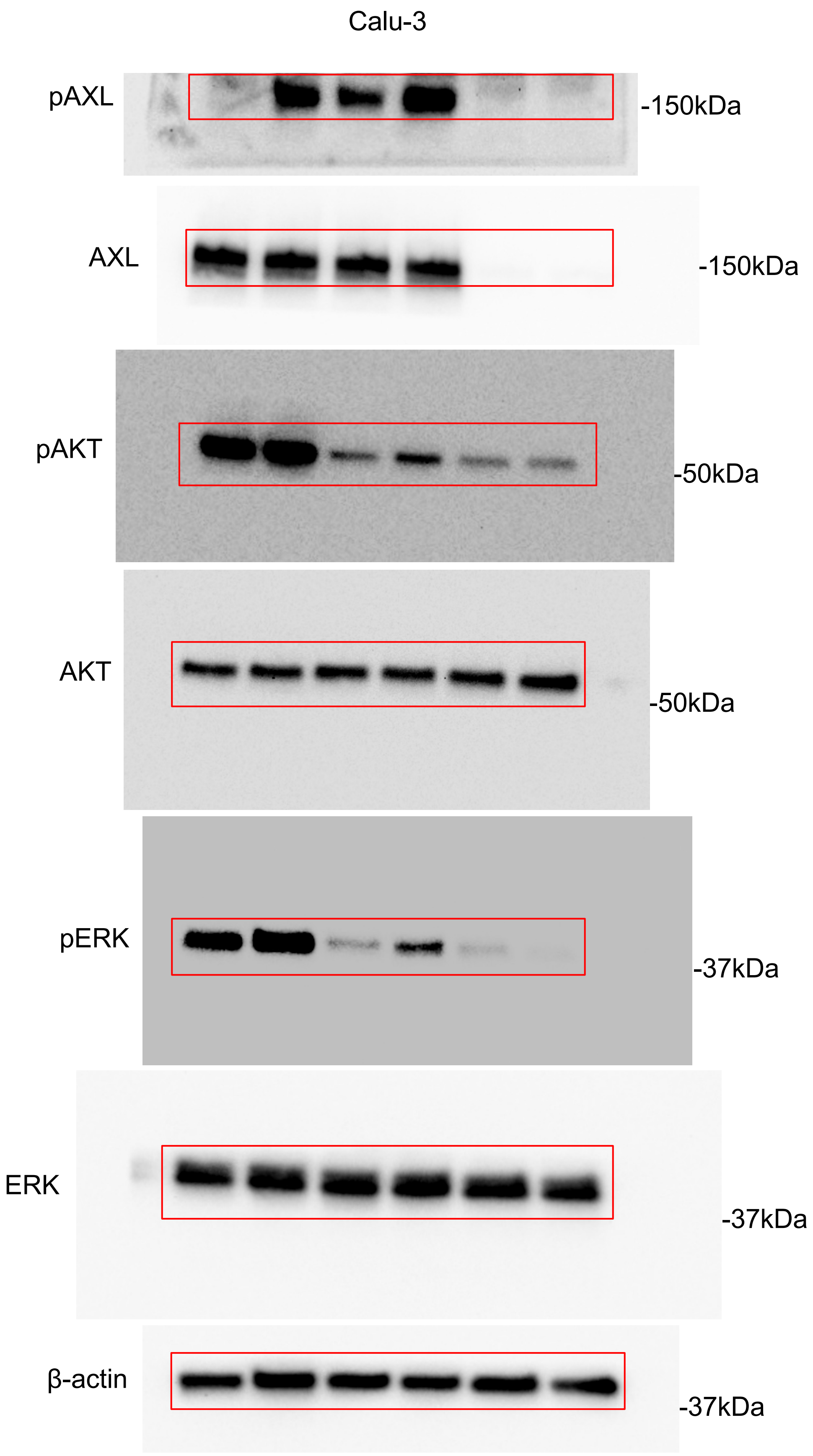
Calu-3
pAXL
-150kDa
AXL
-150kDa
pAKT
-50kDa
AKT
-50kDa
pERK
-37kDa
ERK
-37kDa
β-actin
-37kDa

Figure 3E.

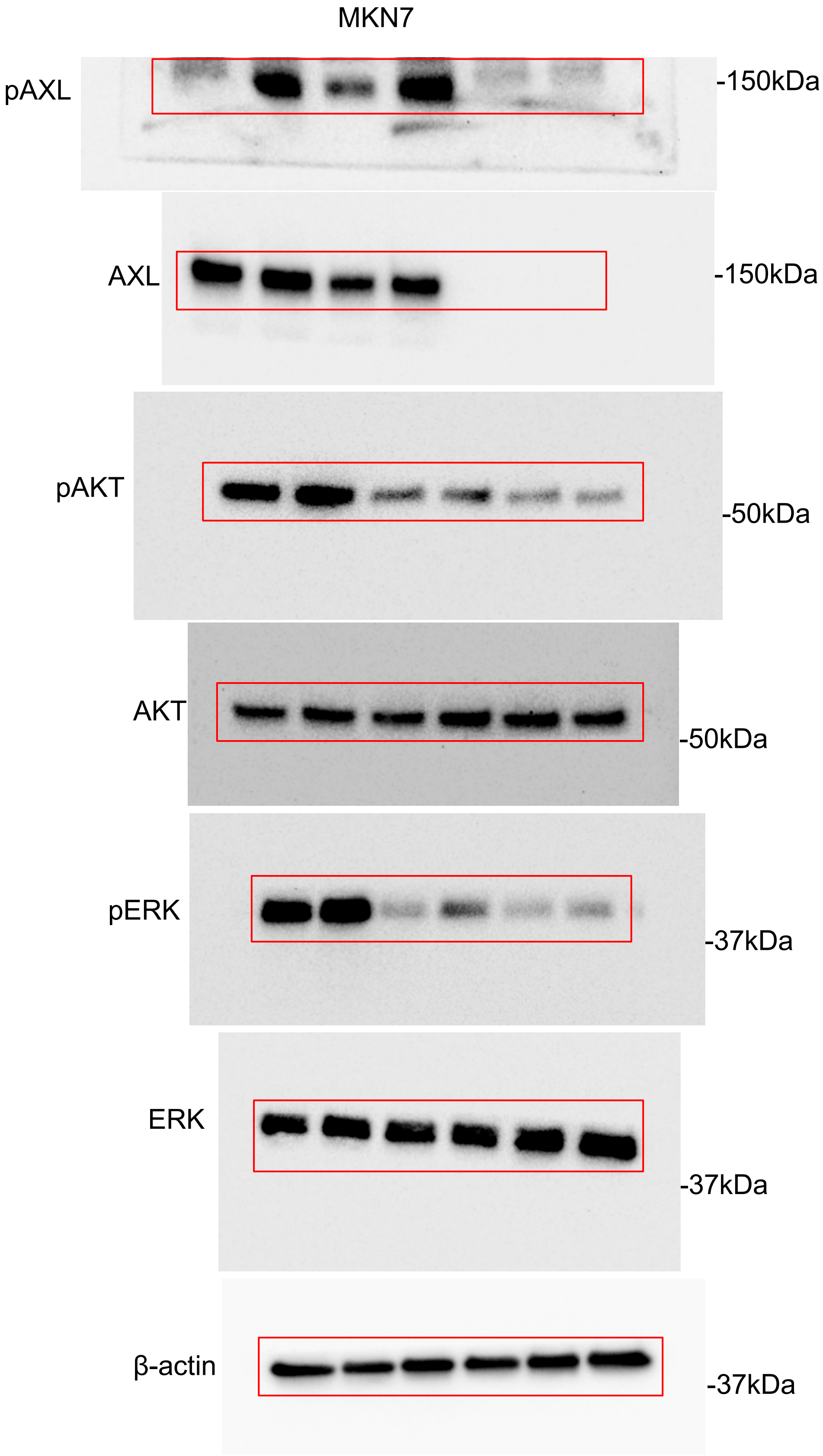
MKN7
pAXL
-150kDa
AXL
-150kDa
pAKT
-50kDa
AKT
-50kDa
pERK
-37kDa
ERK
-37kDa
β-actin
-37kDa

Figure 3F.

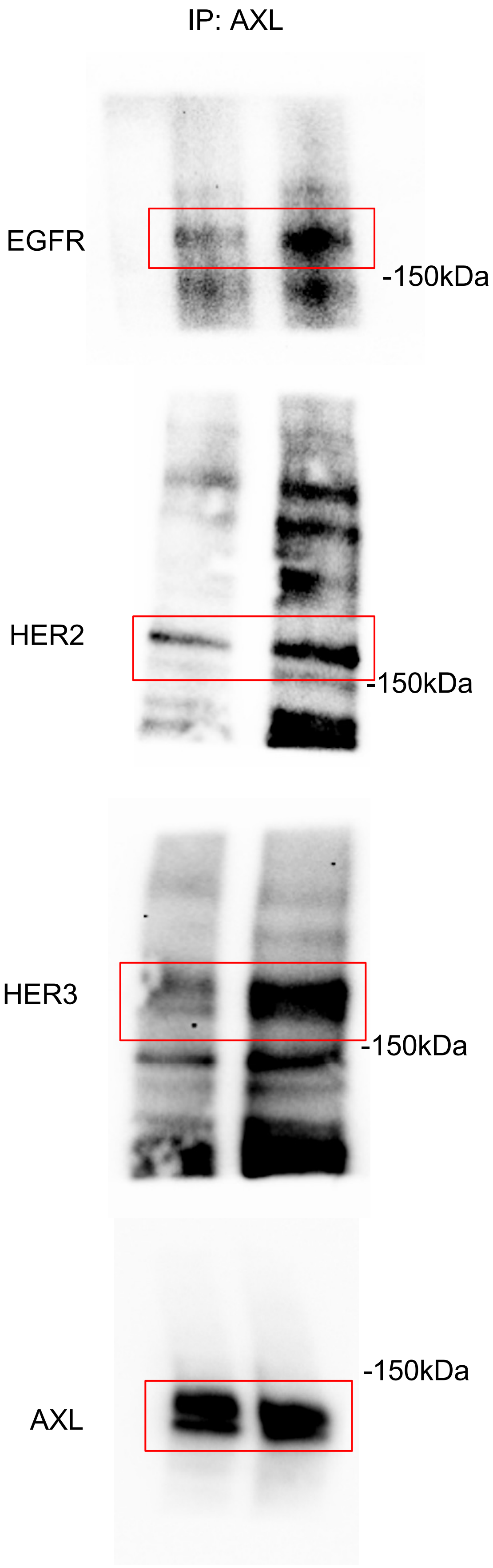

Figure 3F.

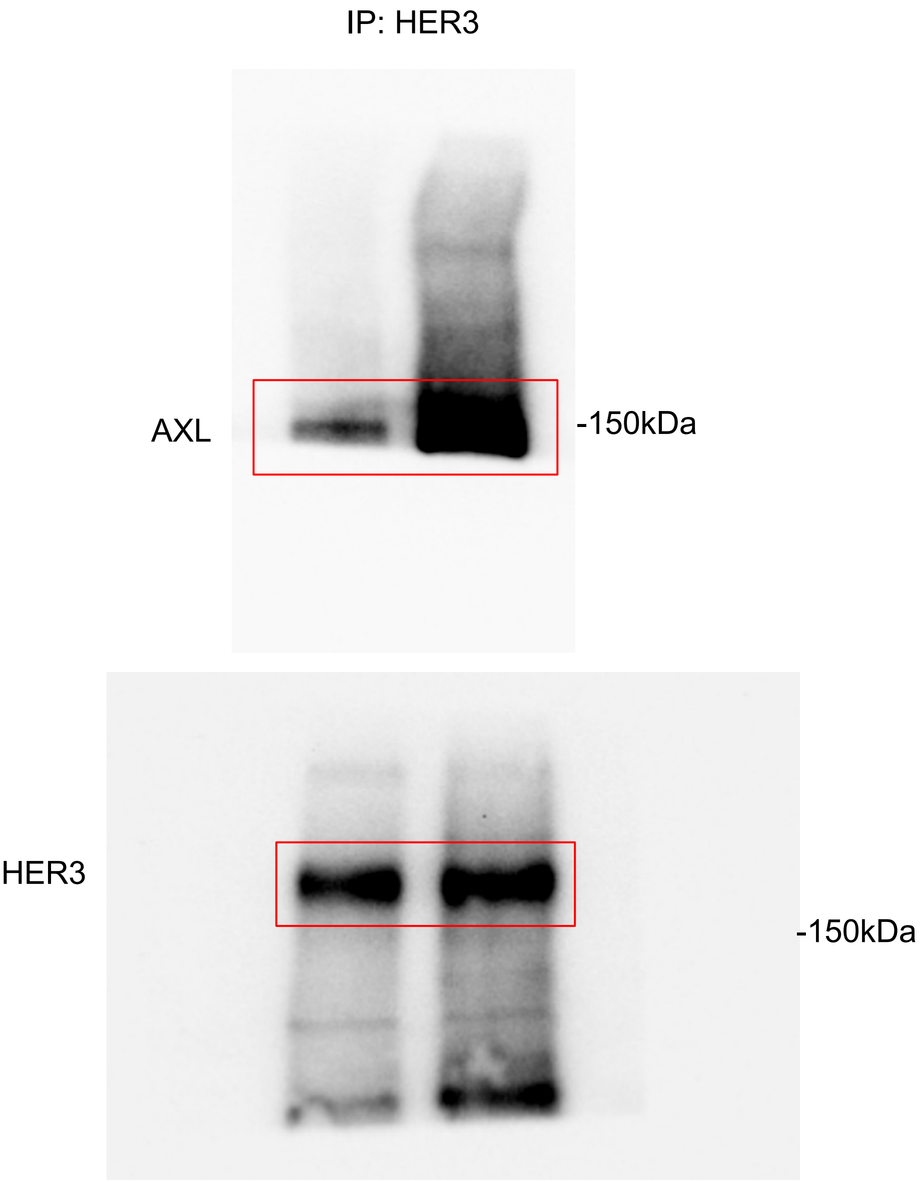

Figure 3F.

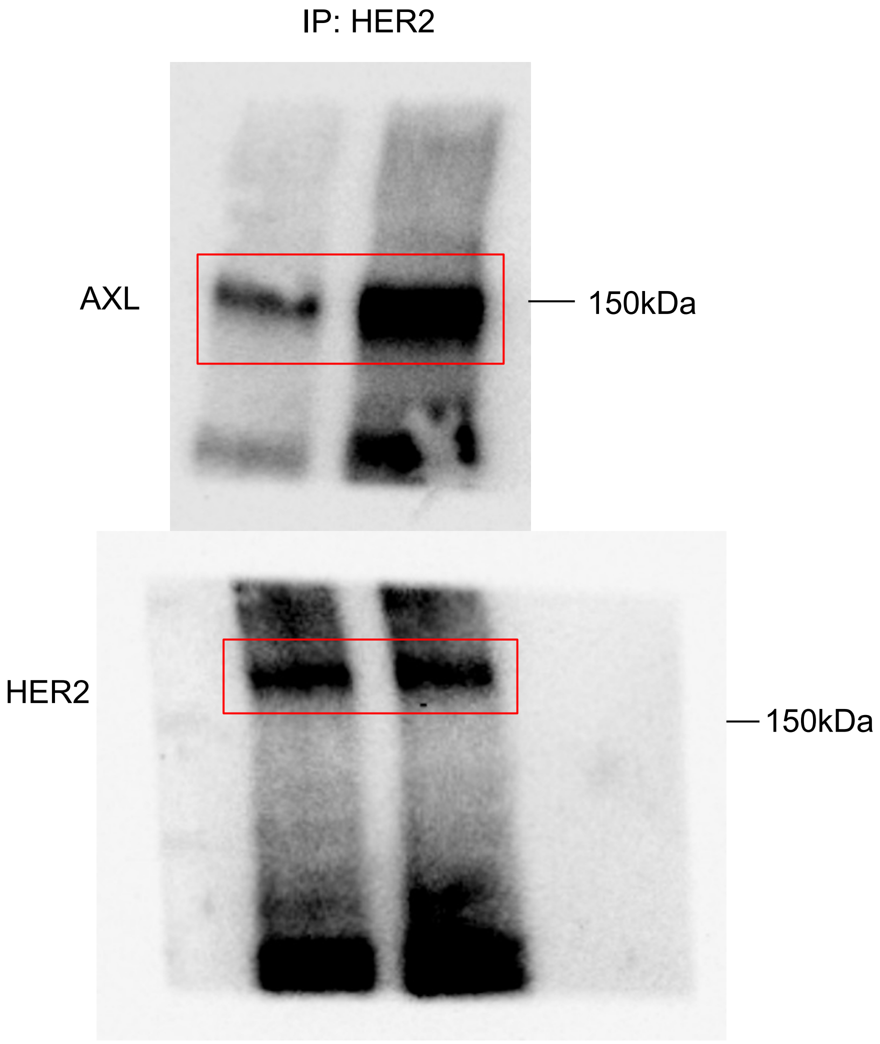

Figure 3F.

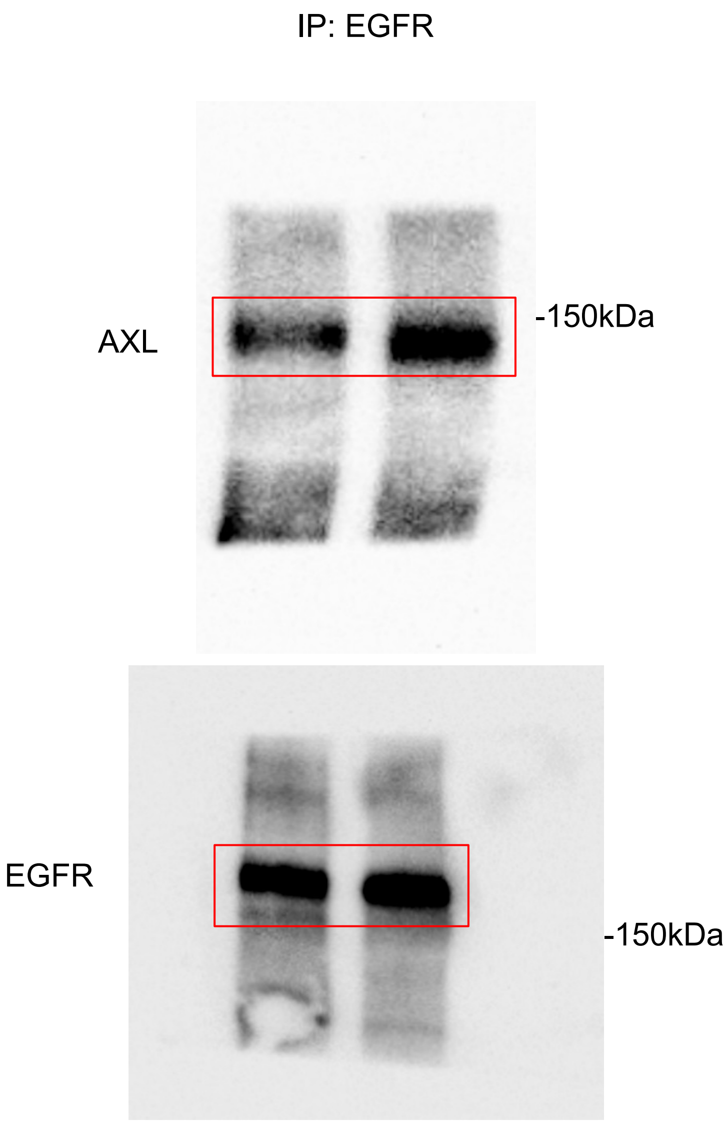

Figure 4B.

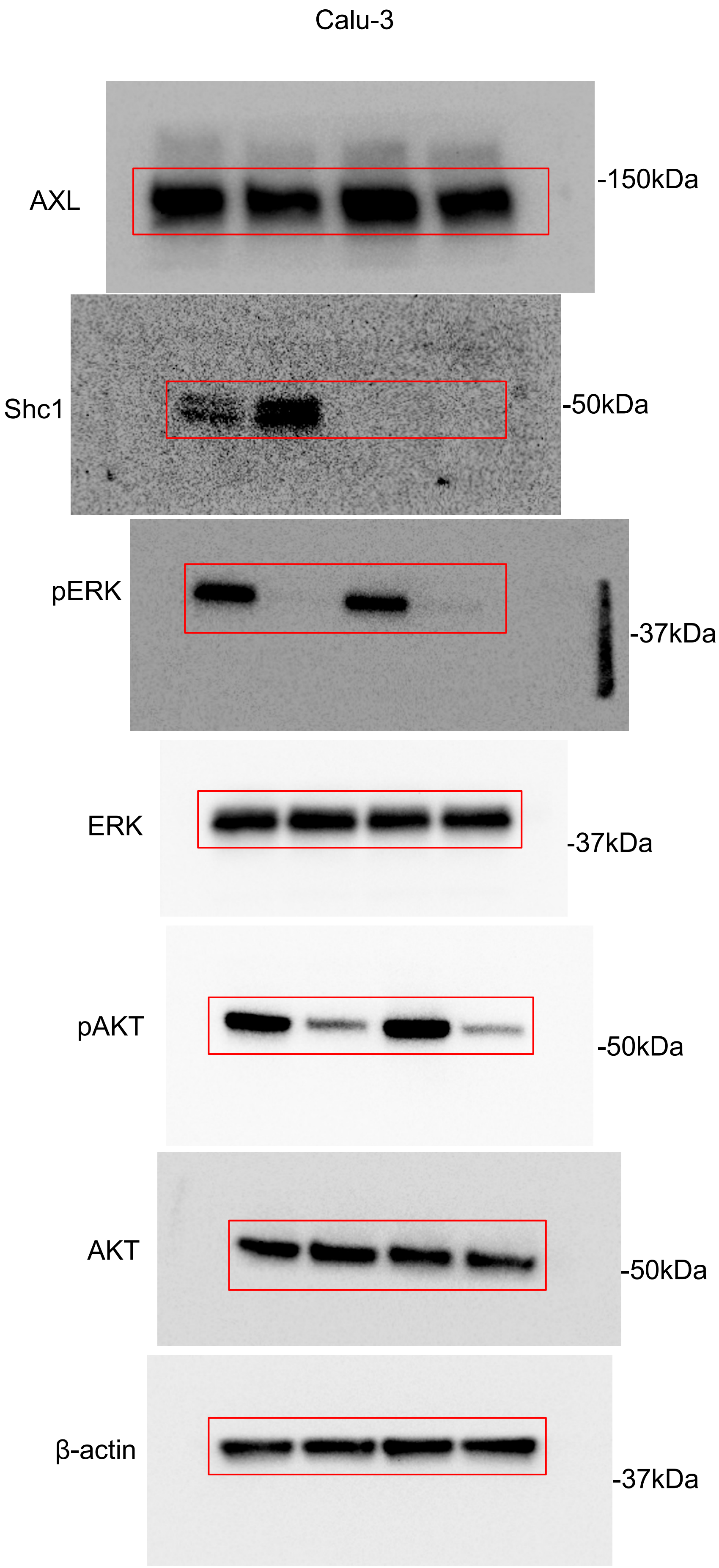

Calu-3
AXL
-150kDa
Shc1
-50kDa
pERK
-37kDa
ERK
-37kDa
pAKT
-50kDa
AKT
-50kDa
β-actin
-37kDa

Figure 4B.

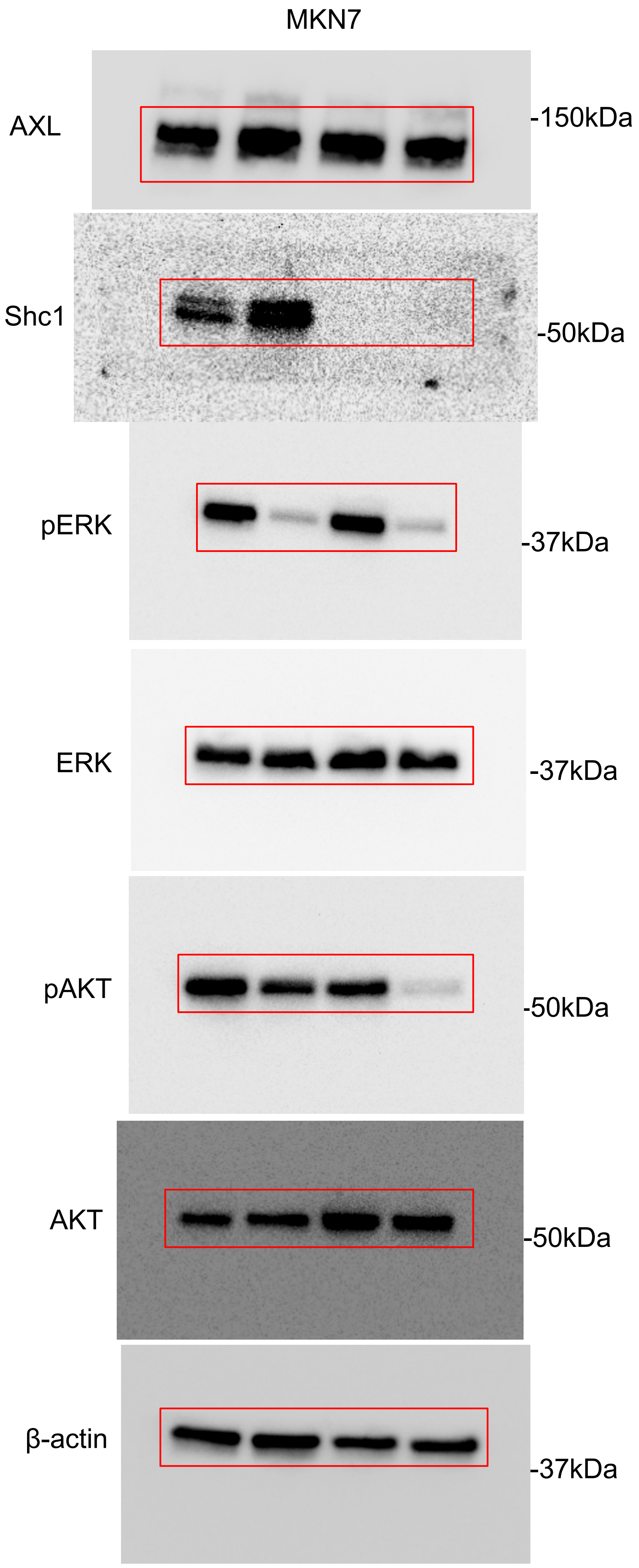
MKN7
AXL
-150kDa
Shc1
-50kDa
pERK
-37kDa
ERK
-37kDa
pAKT
-50kDa
AKT
-50kDa
β-actin
-37kDa

Figure 4C.

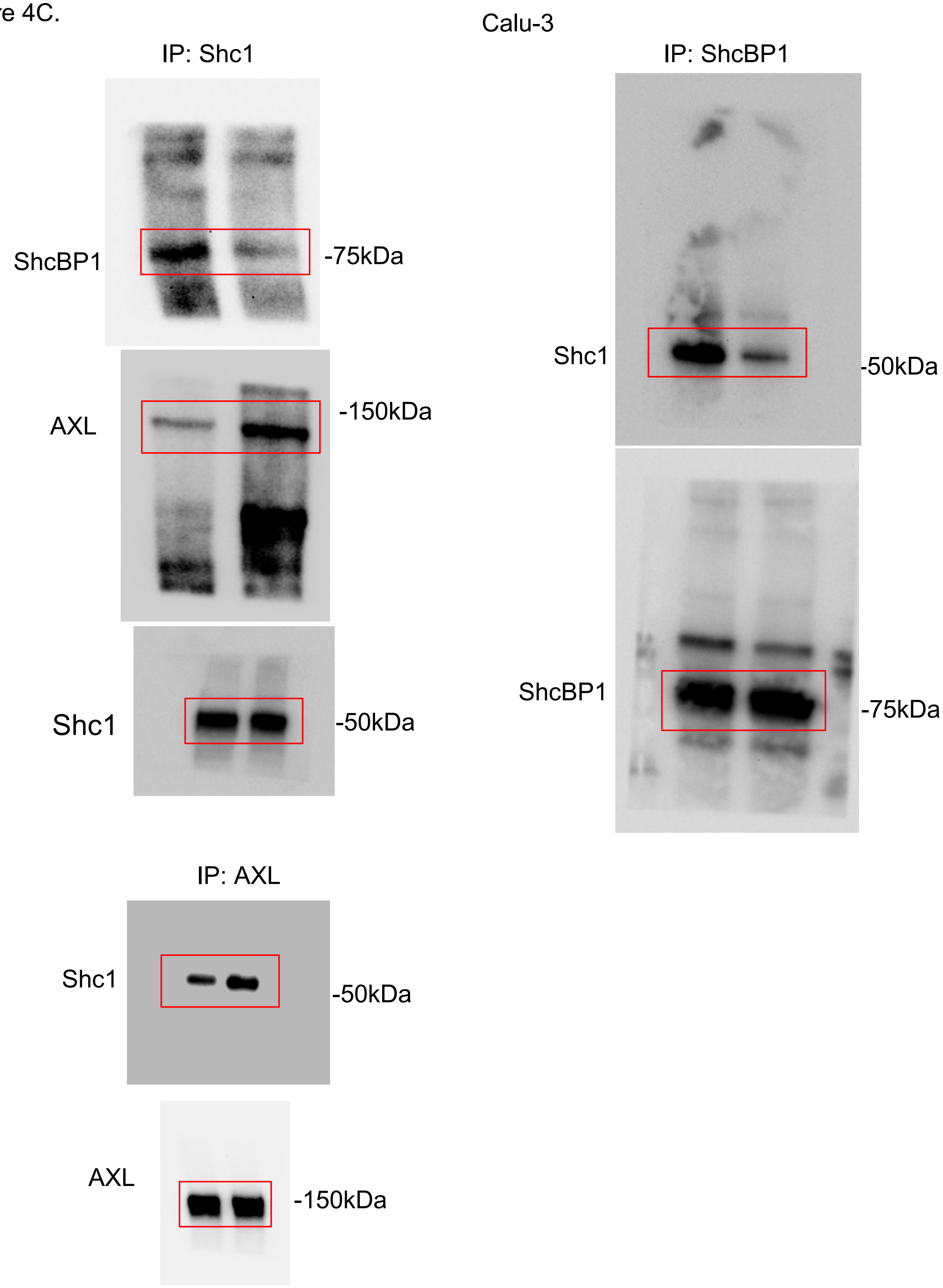
Calu-3
IP: Shc1
ShcBP1
-75kDa
AXL
-150kDa
Shc1
-50kDa
IP: ShcBP1
Shc1
-50kDa
ShcBP1
-75kDa
IP: AXL
Shc1
-50kDa
AXL
-150kDa

Figure 4C.

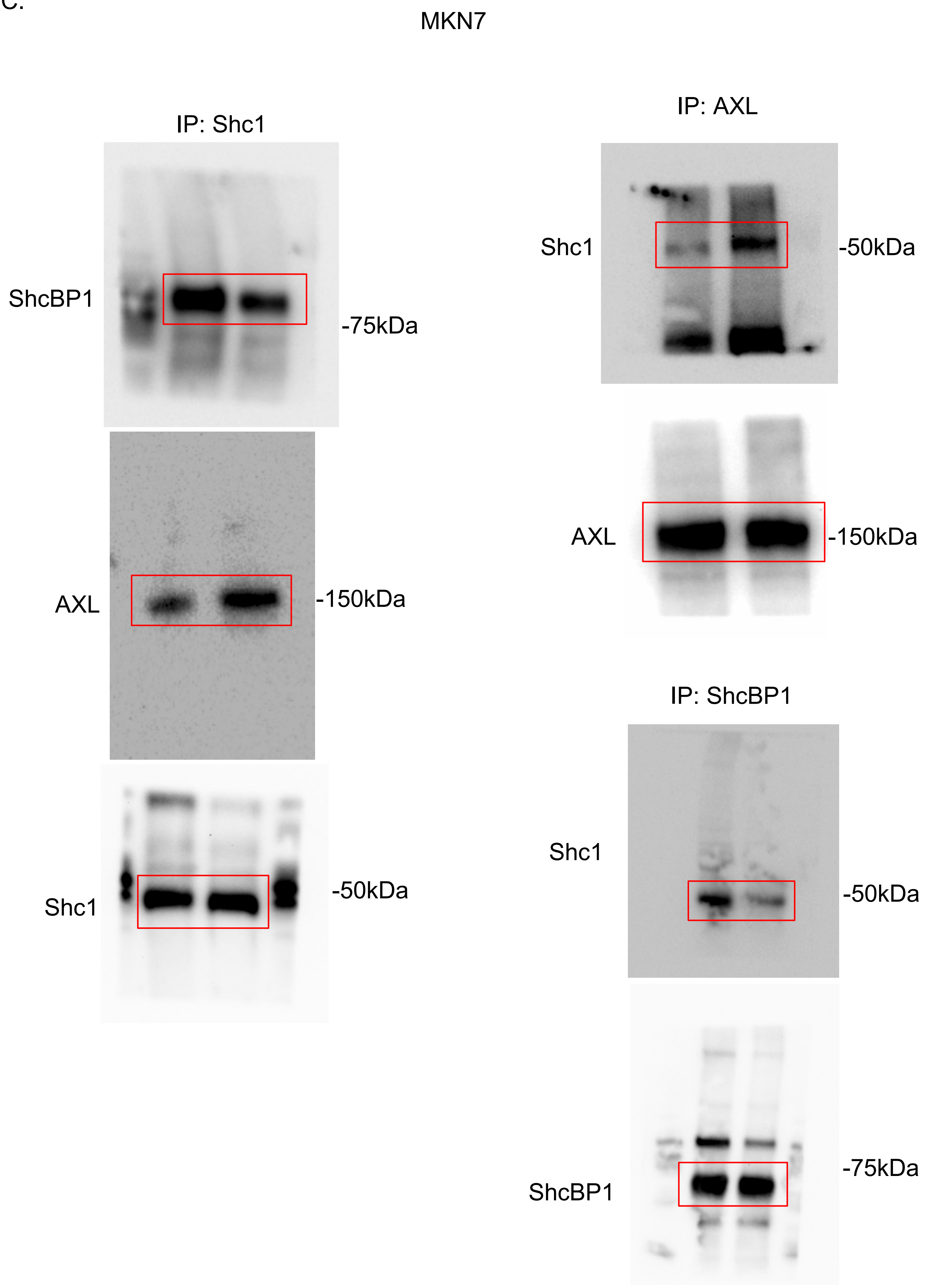
MKN7
IP: Shc1
ShcBP1
-75kDa
AXL
-150kDa
Shc1
-50kDa
IP: AXL
Shc1
-50kDa
AXL
-150kDa
IP: ShcBP1
Shc1
-50kDa
ShcBP1
-75kDa

Figure 4D.

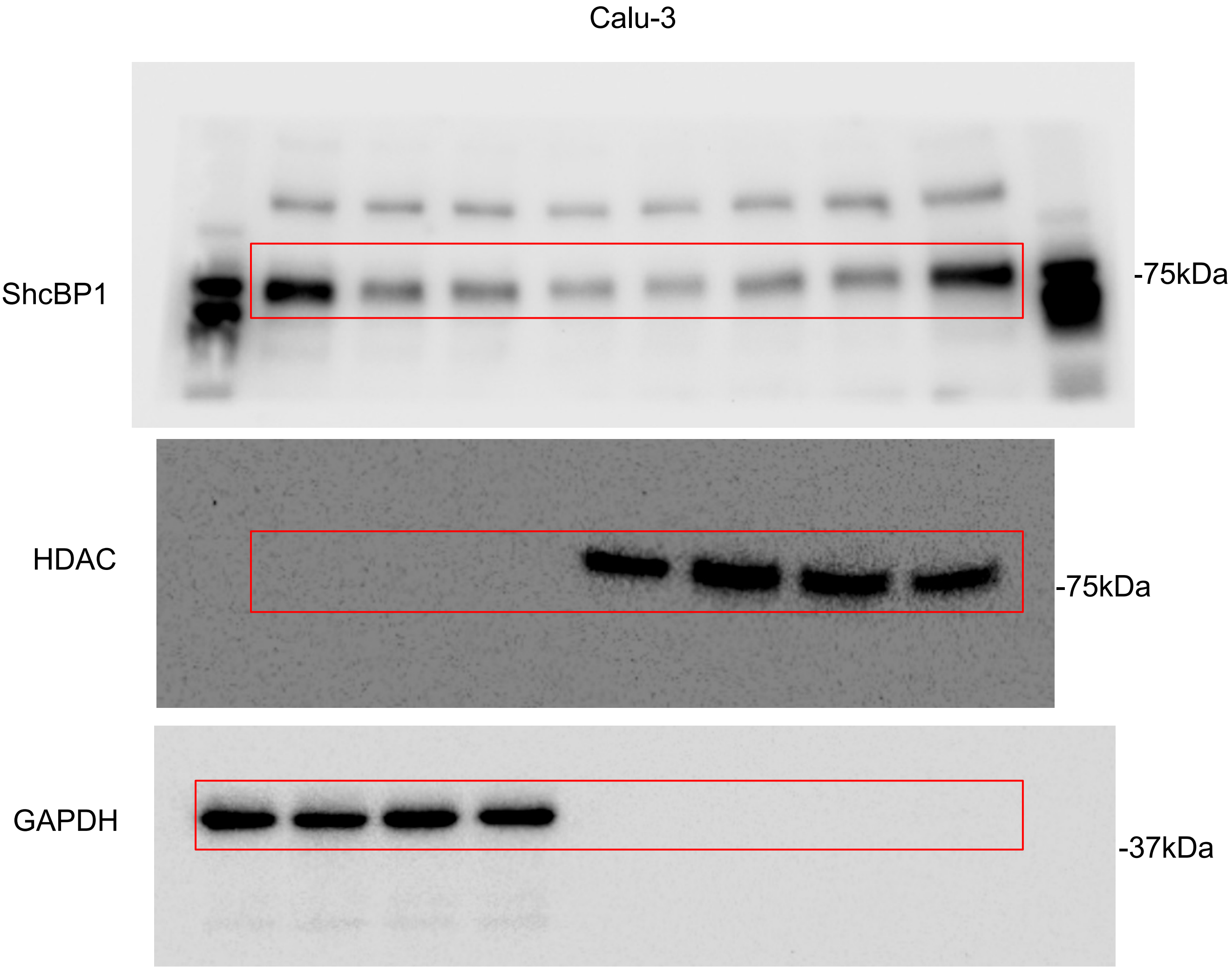

Figure 4D.

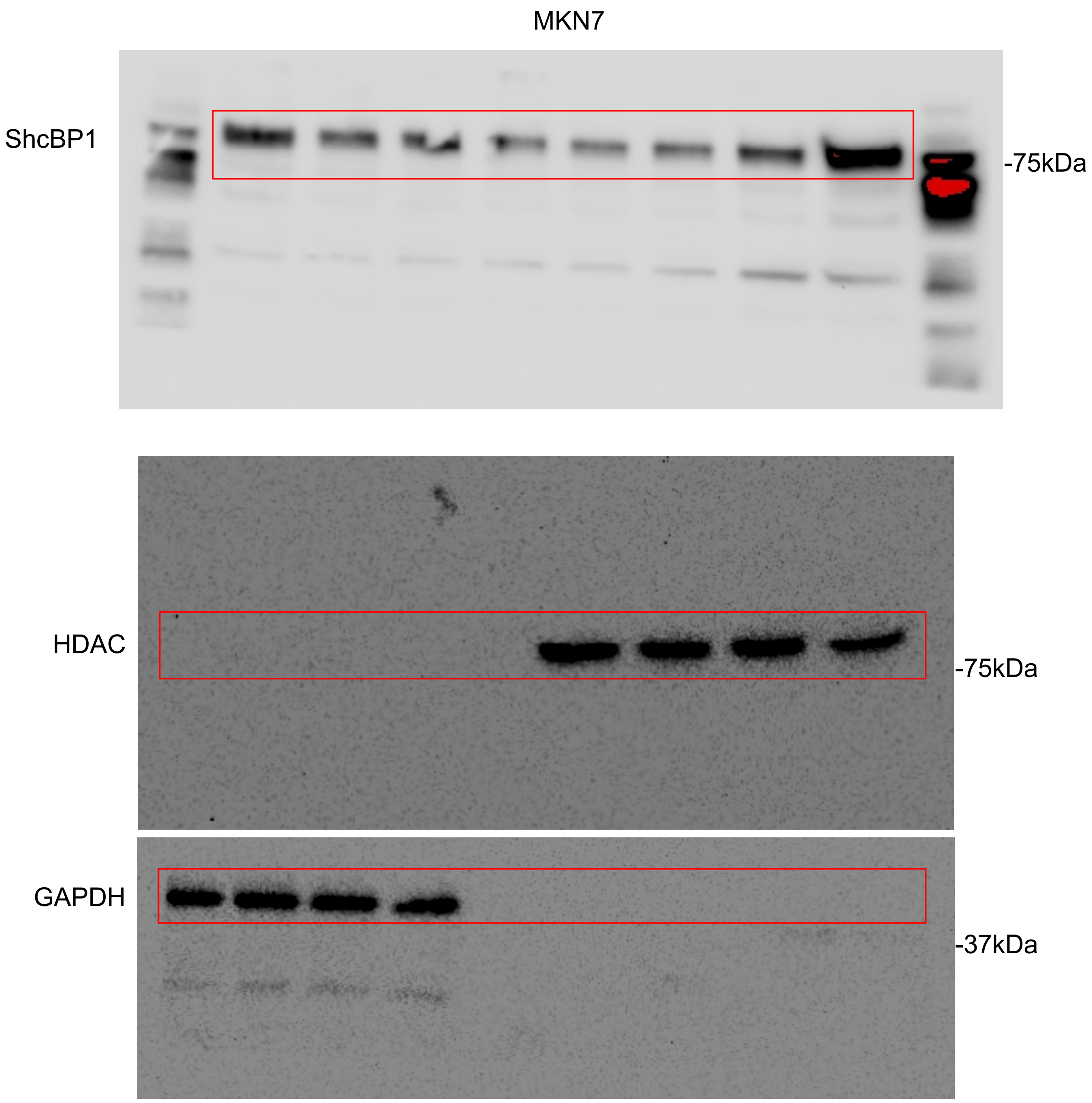

Figure 5D.

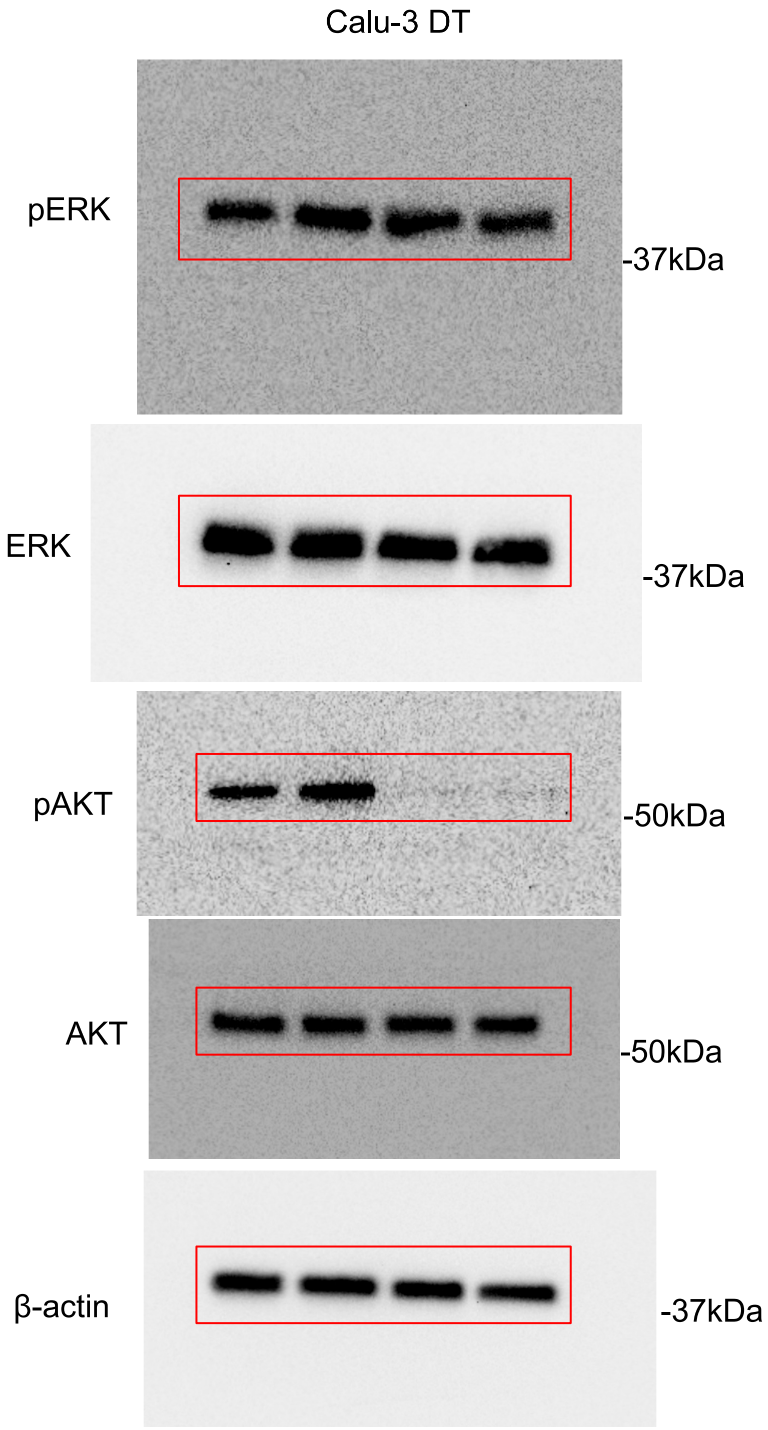
Calu-3 DT
pERK
-37kDa
ERK
-37kDa
pAKT
-50kDa
AKT
-50kDa
β-actin
-37kDa

Figure 5D.

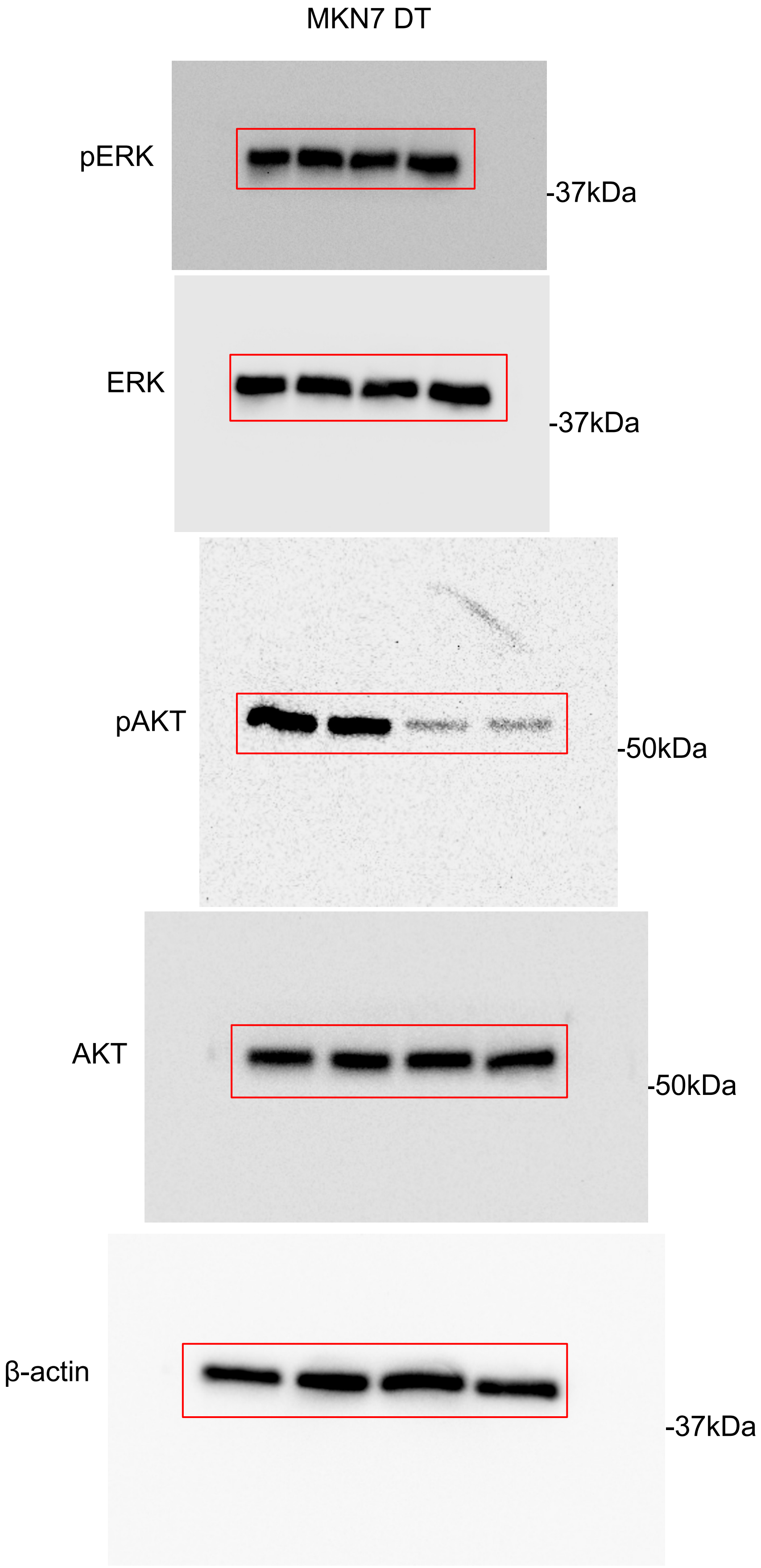
MKN7 DT
pERK
-37kDa
ERK
-37kDa
pAKT
-50kDa
AKT
-50kDa
β-actin
-37kDa

Figure 6B.

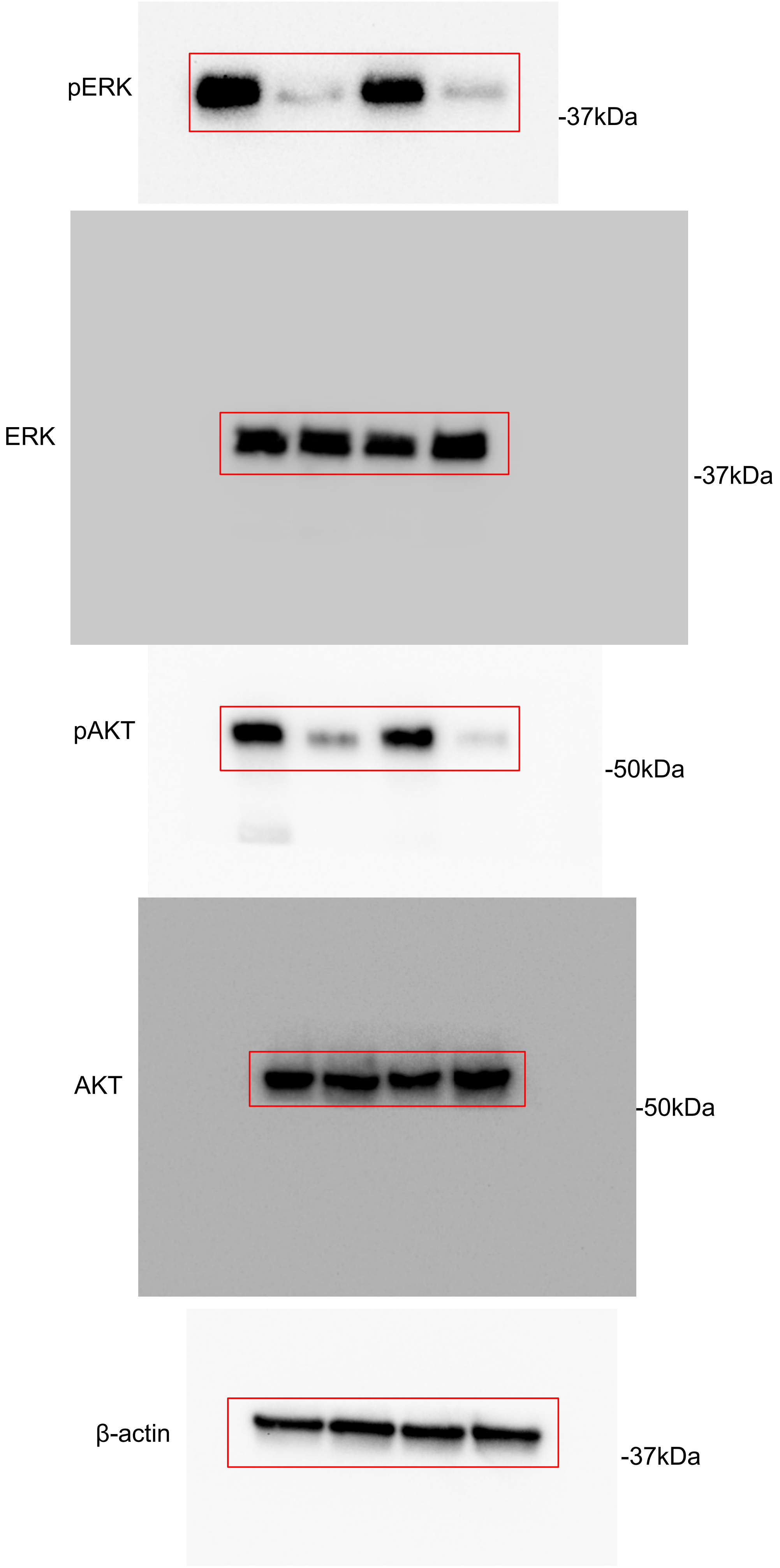

Supplementary Figure 4.

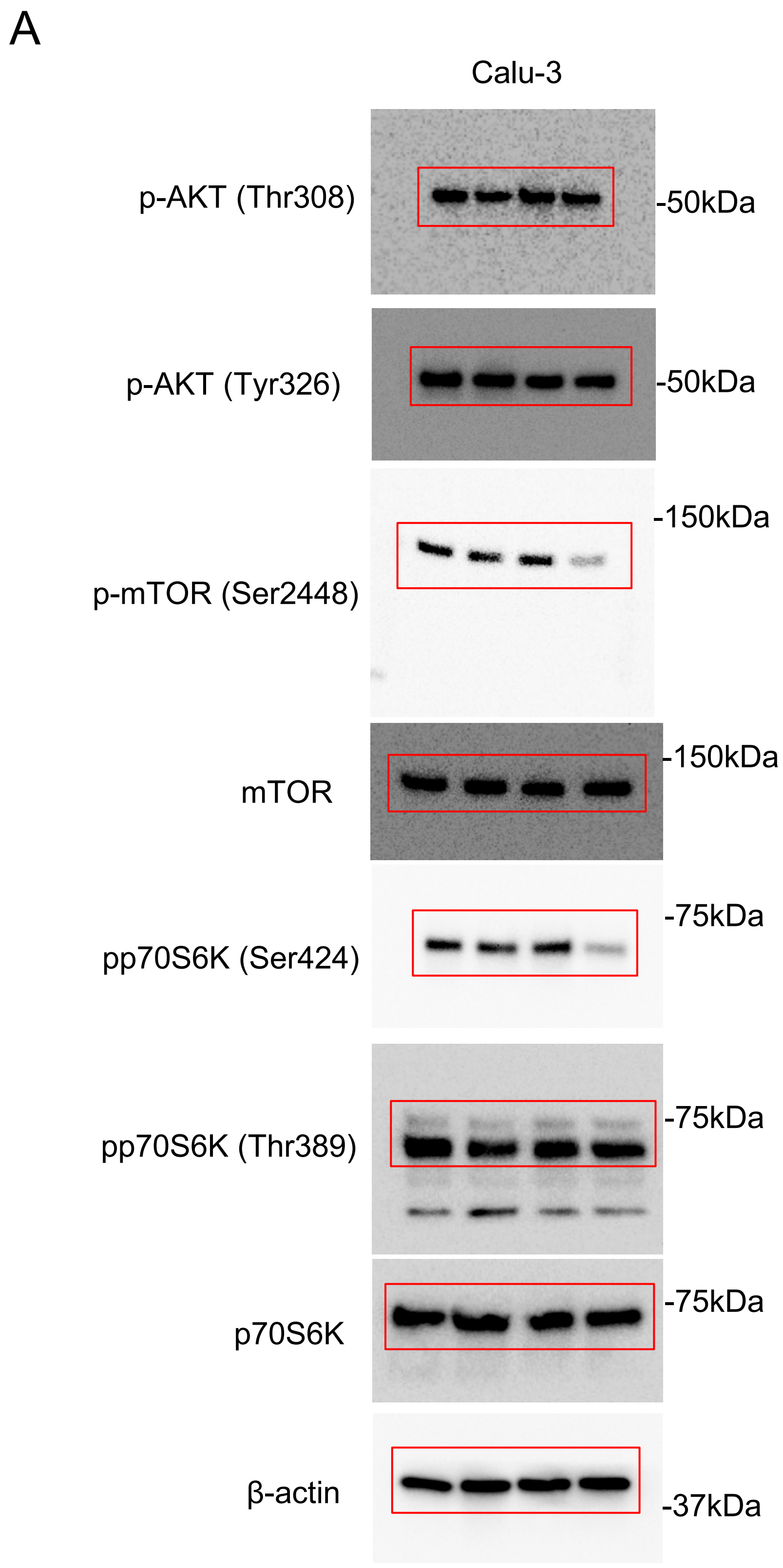

Supplementary Figure 4.

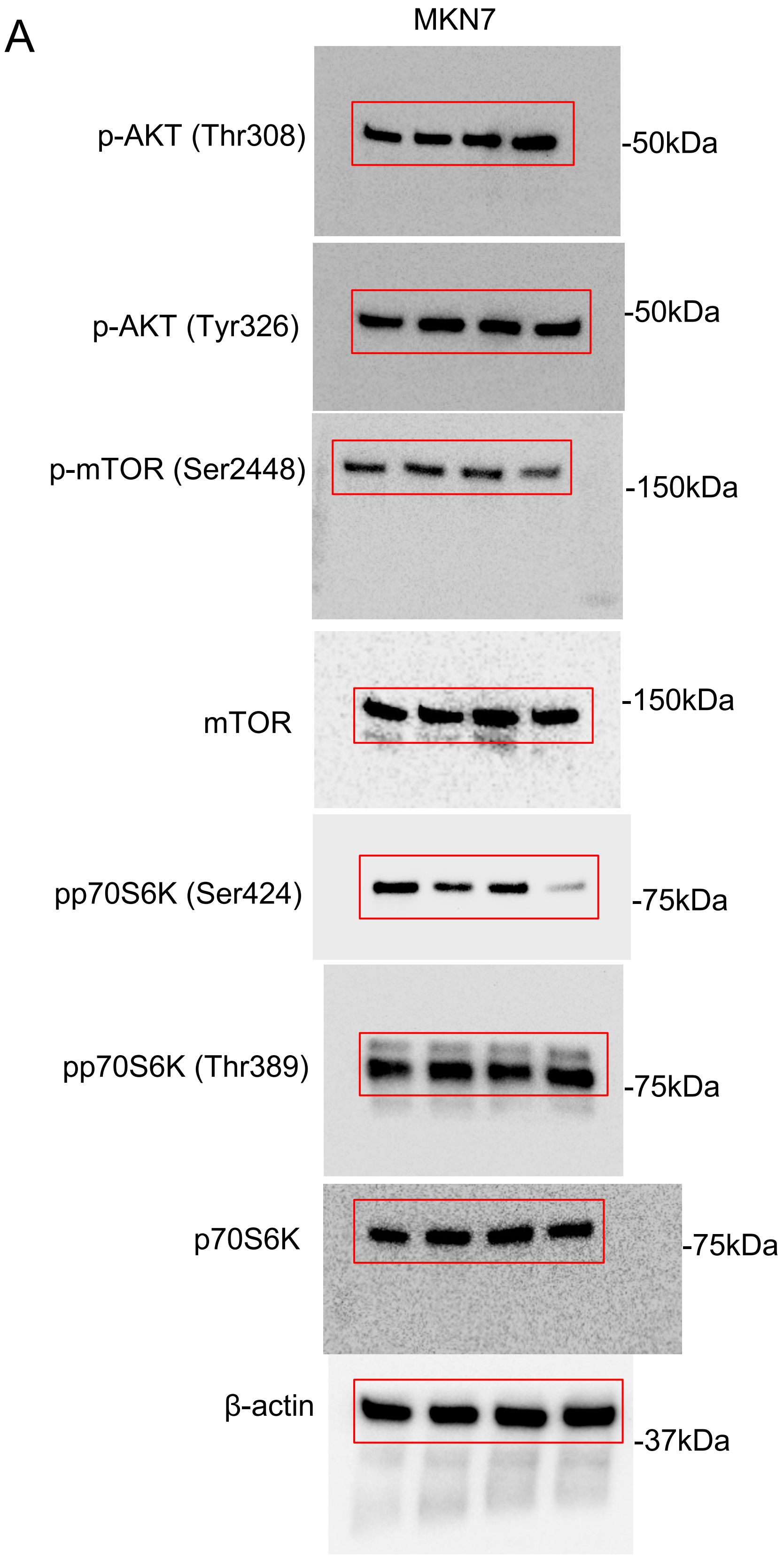

Supplementary Figure 4.

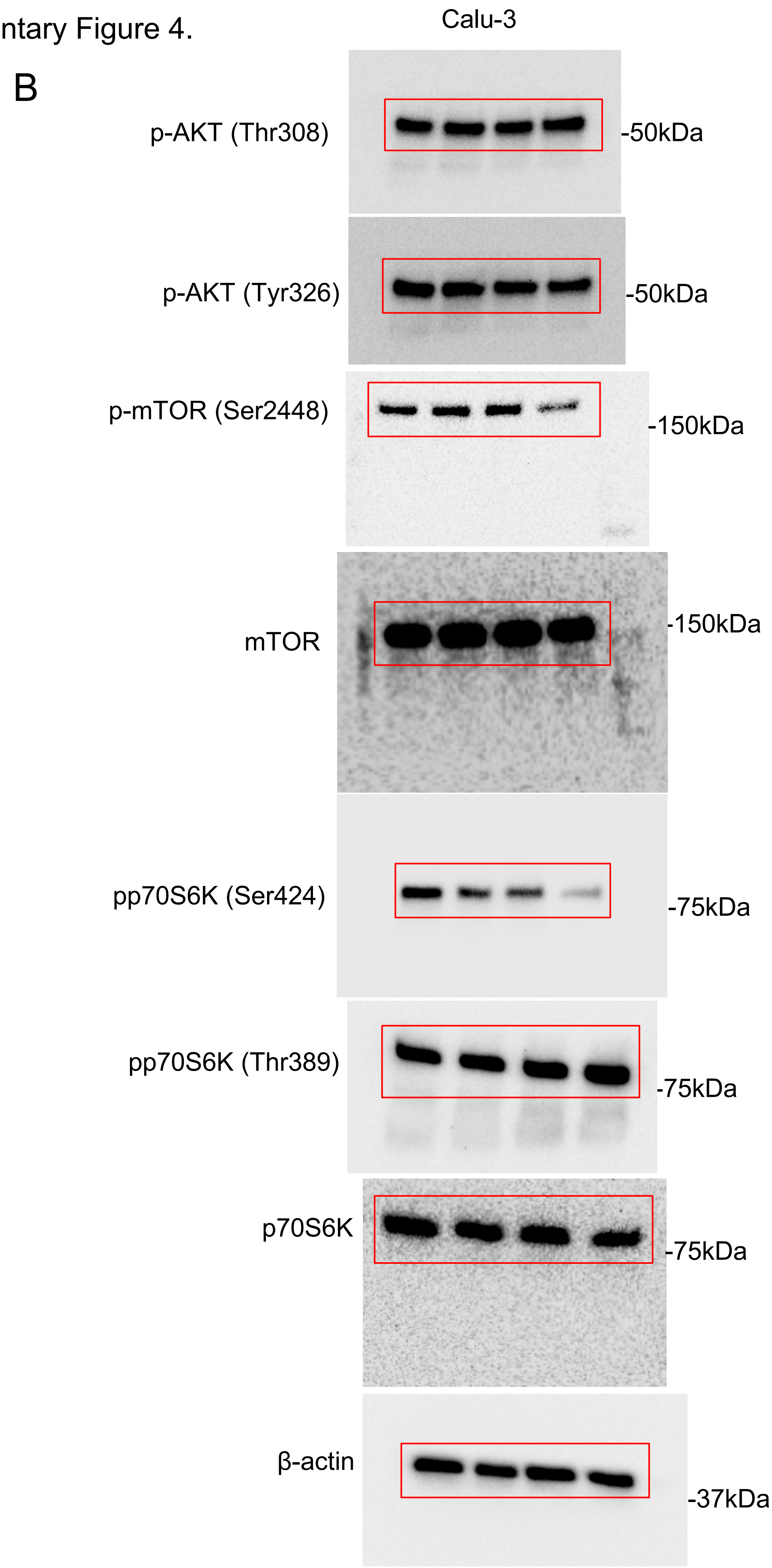

Supplementary Figure 4.

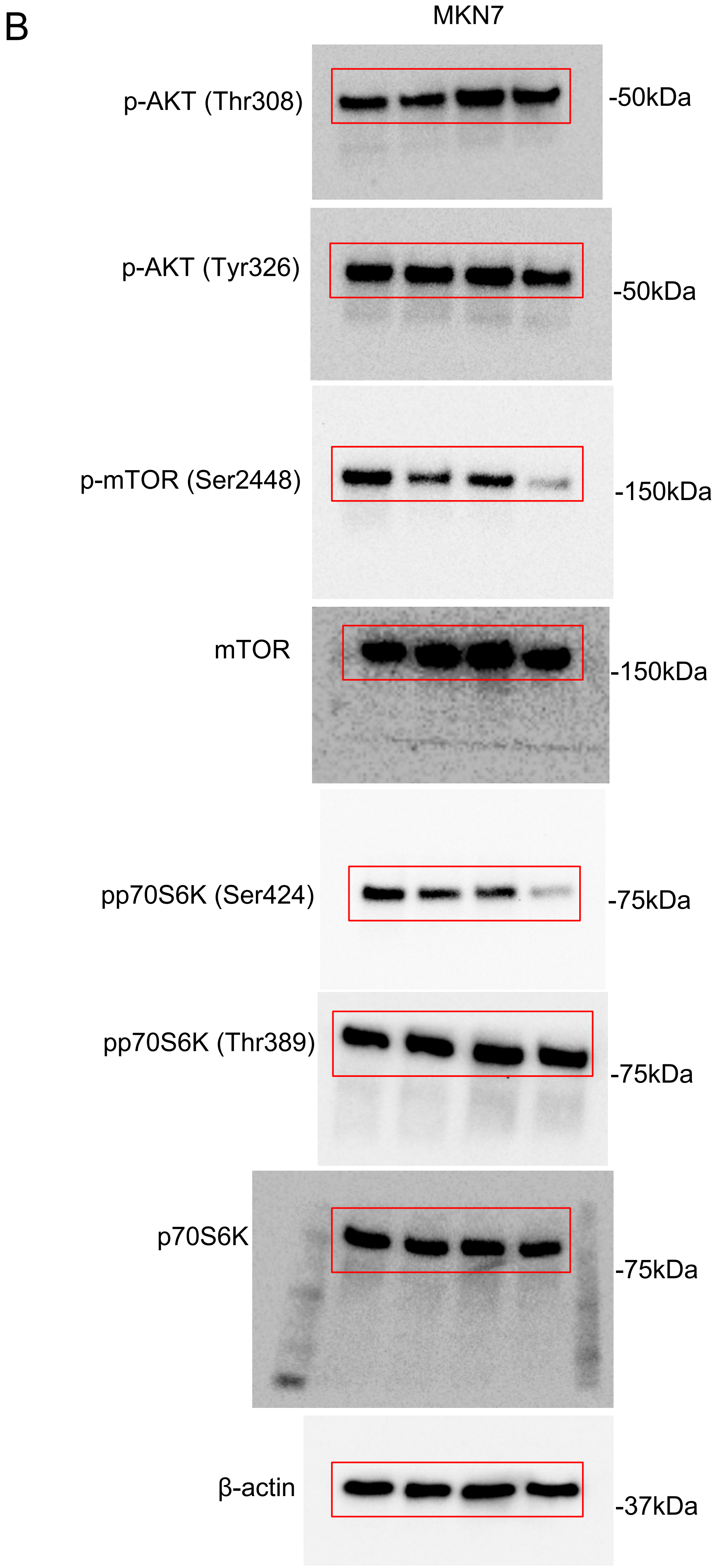

Supplementary Figure 13.

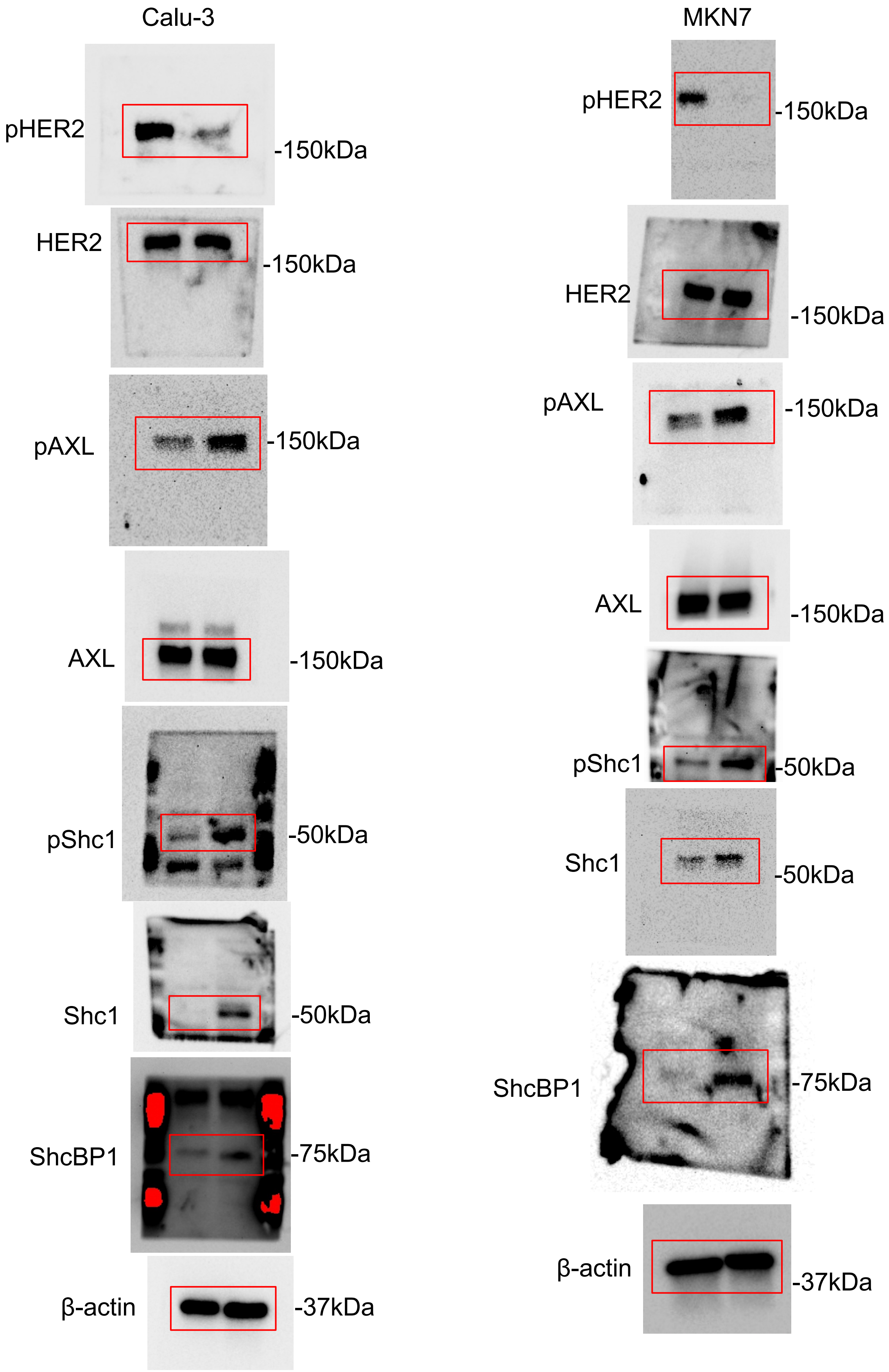

Supplementary Figure 14.

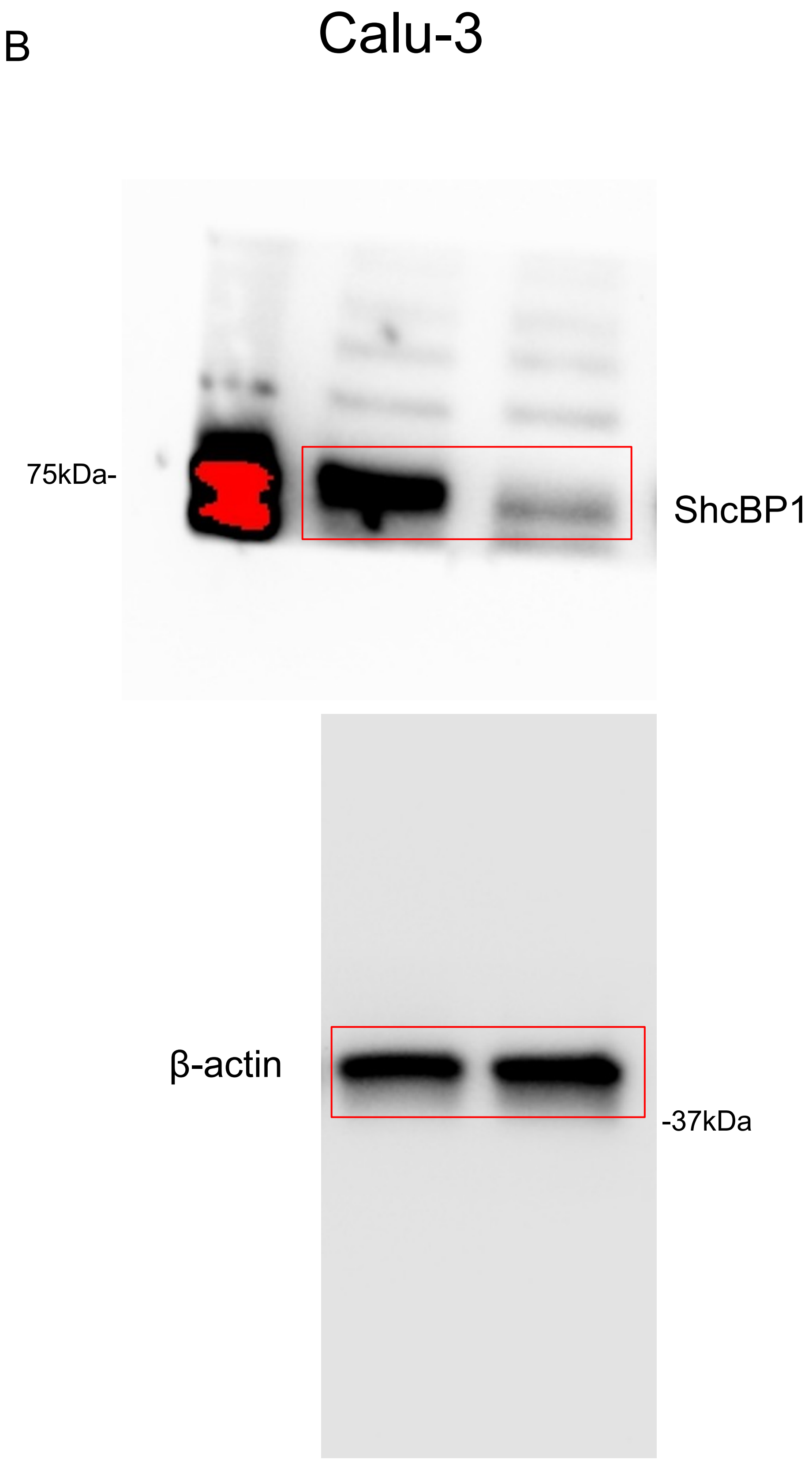

Supplementary Figure 14.

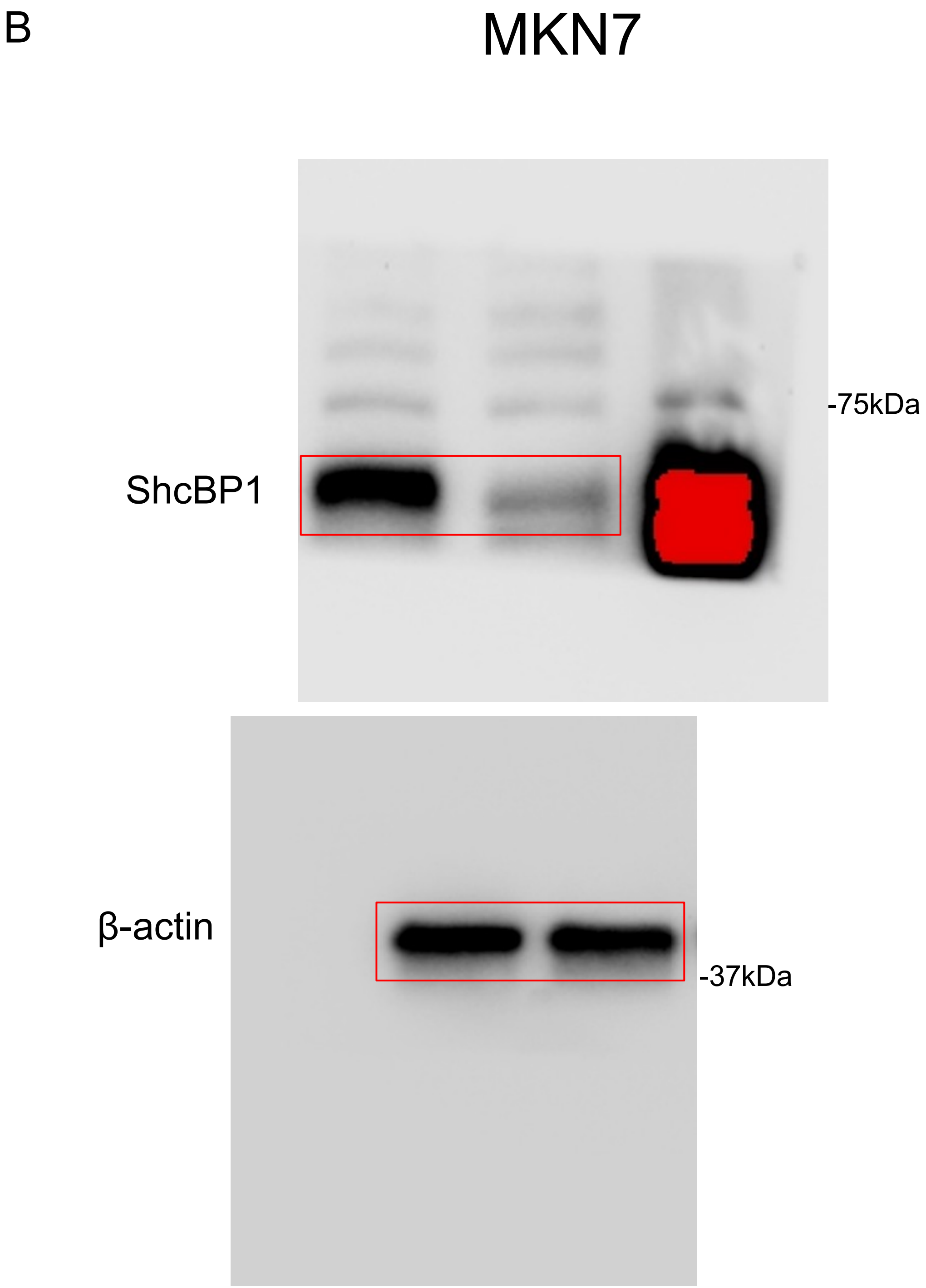

Supplementary Figure 17.

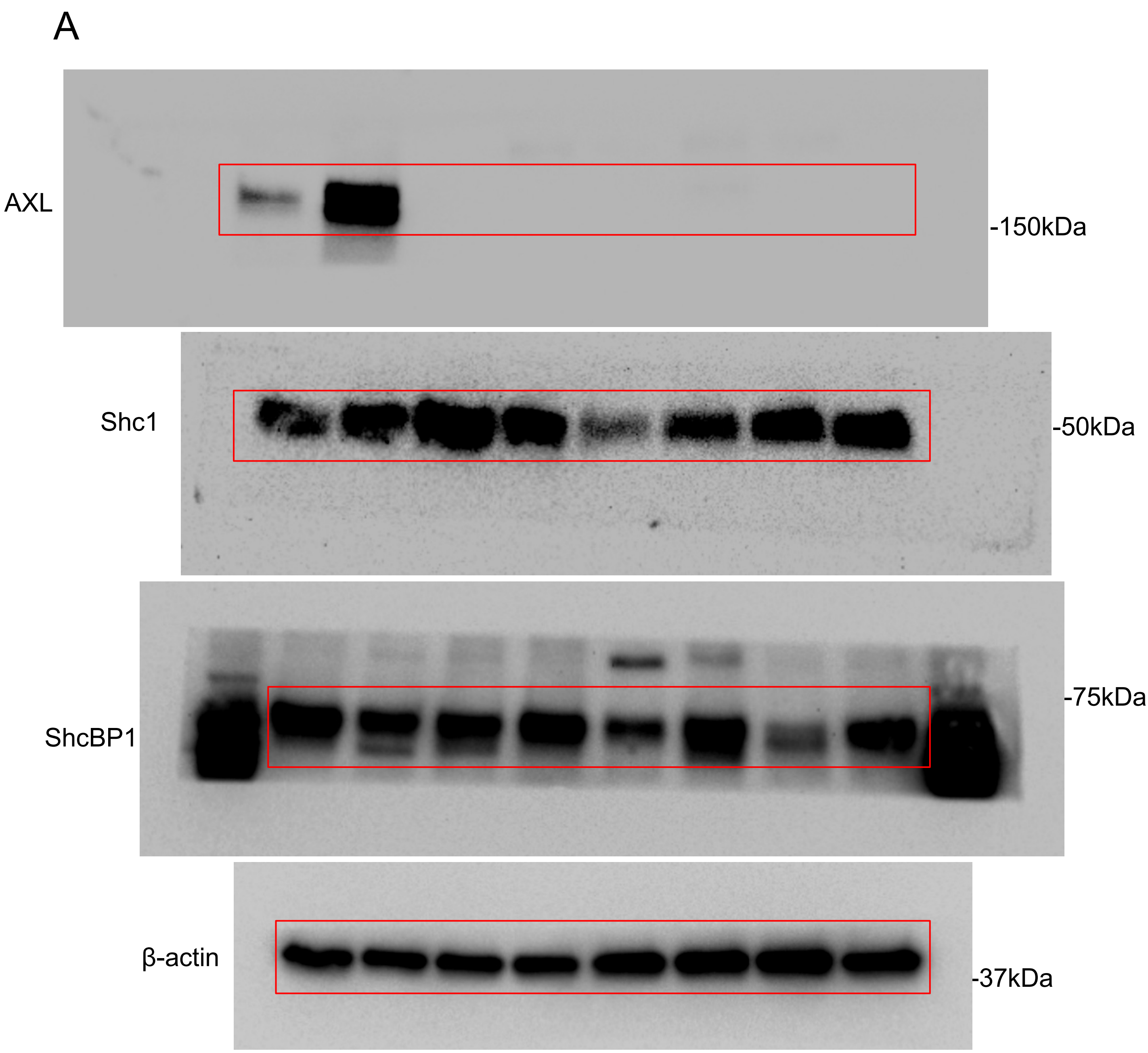

Supplementary Figure 18.

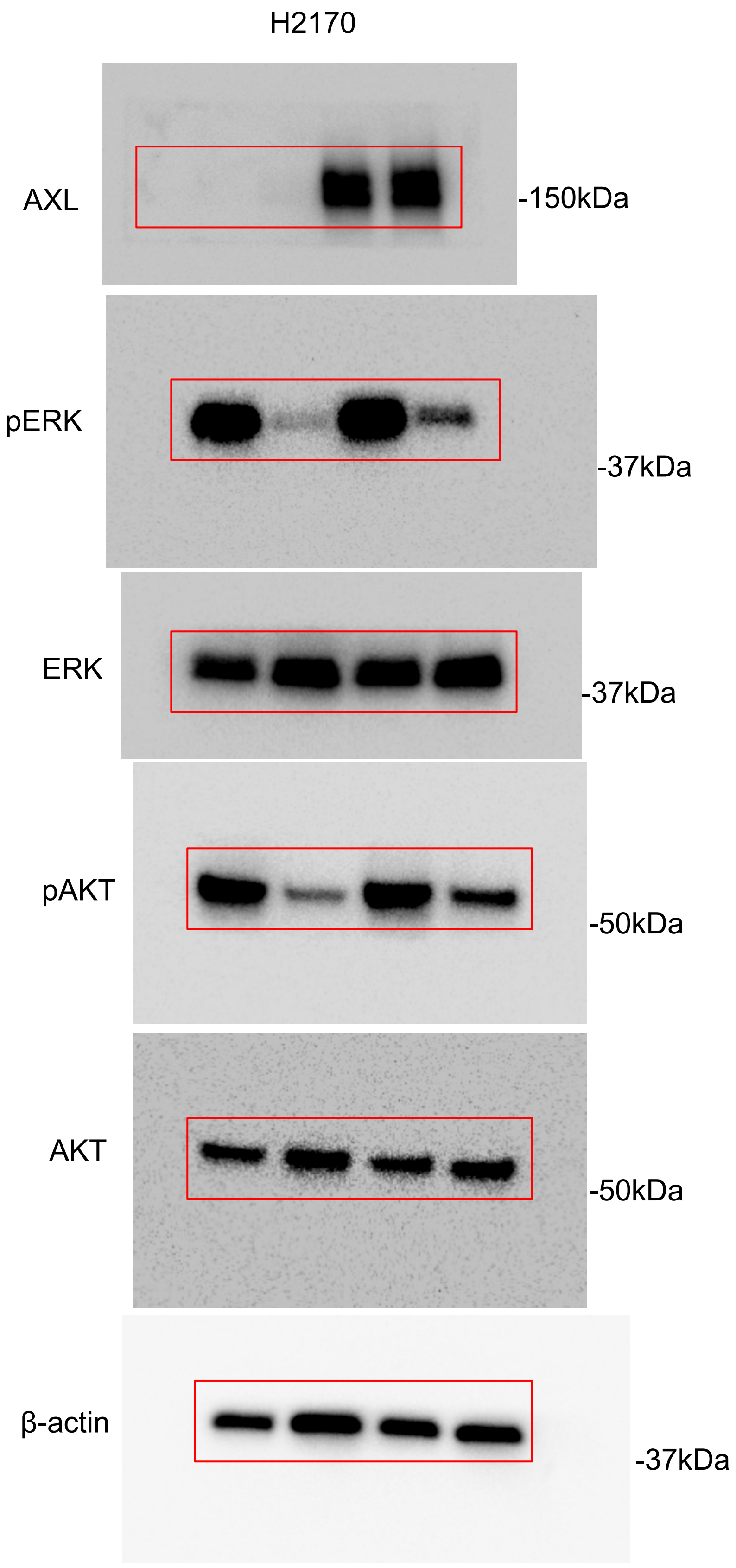

Supplementary Figure 18.

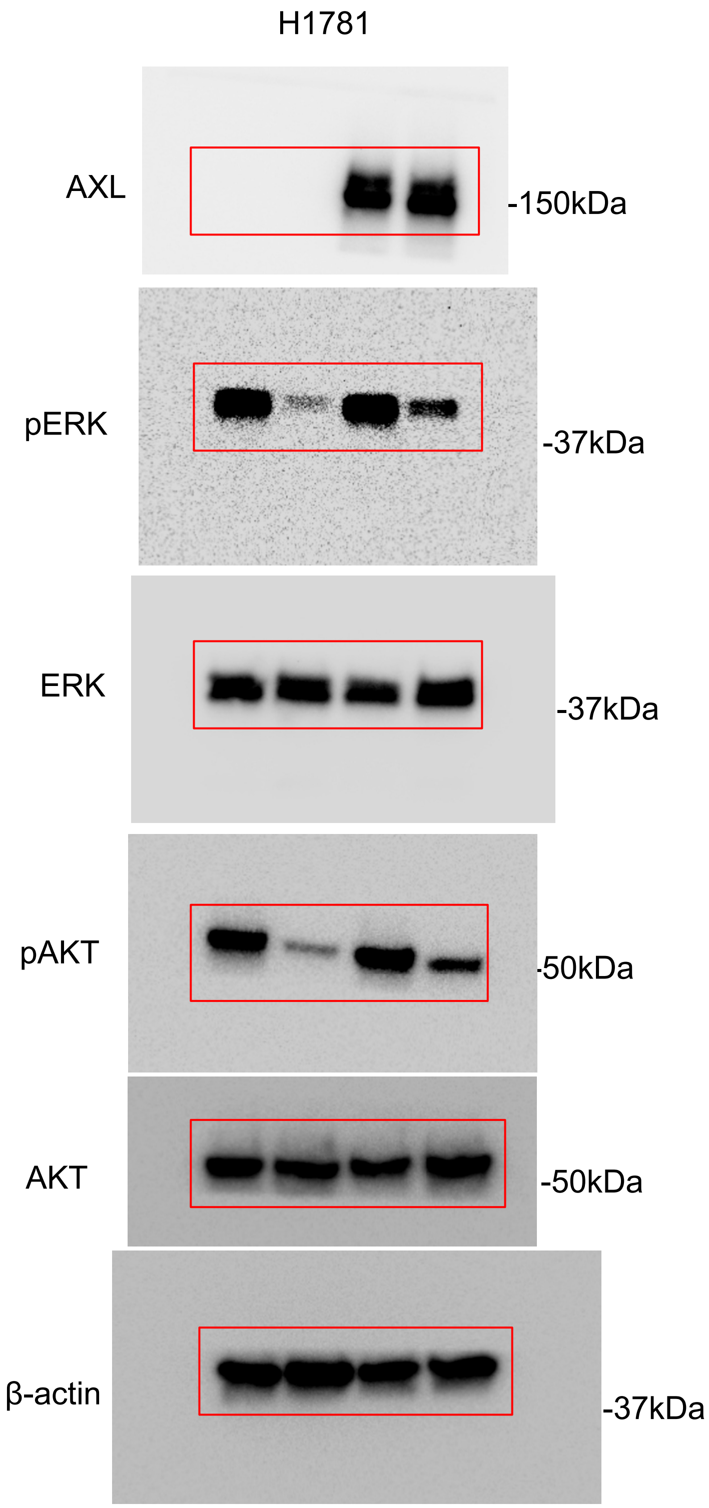

Supplementary Figure 21.

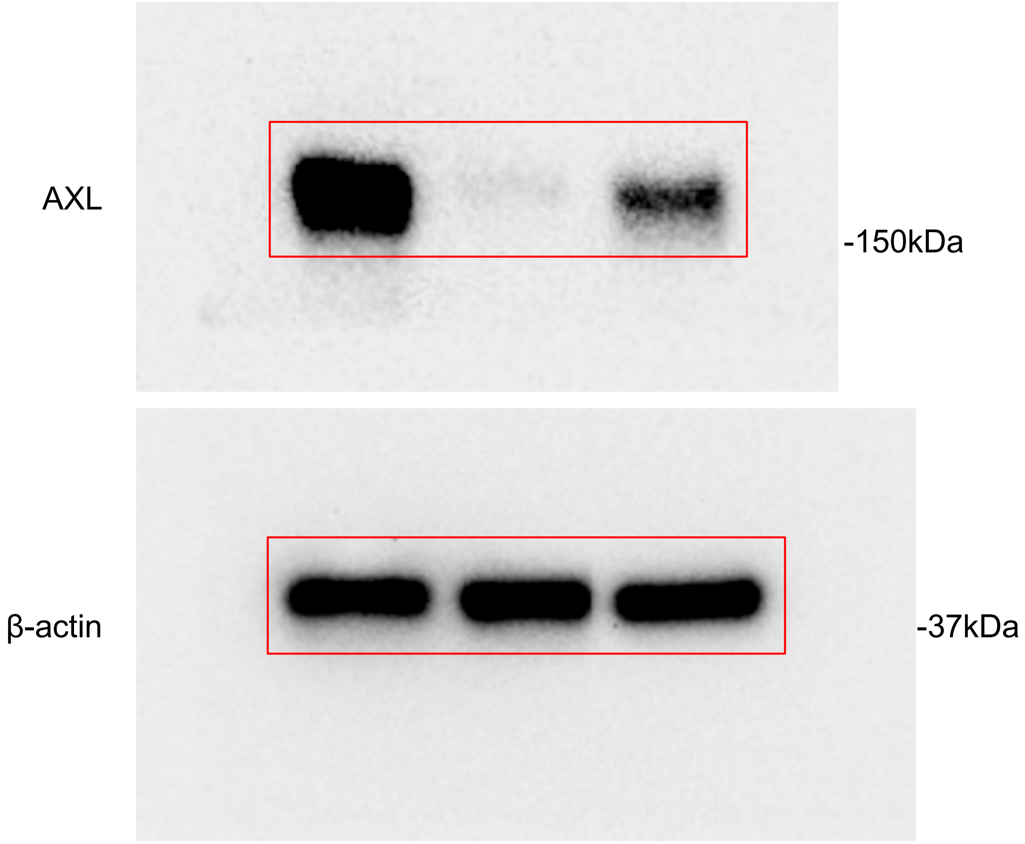

Supplement: Supplementary file 1 — 41698_2026_1385_MOESM1_ESM [file 41698_2026_1385_MOESM1_ESM.pdf]
